# Supplementary material for: Conserved functional antagonism of CELF and MBNL proteins controls stem cell-specific alternative splicing in planarians
Source: eLife. 2016 Aug 9;5:e16797. doi: 10.7554/eLife.16797 (PMC4978528; doi:10.7554/eLife.16797)
Supplement: Figure 3—source data 2. — (A) Comparison between S. mediterranea and D. japonica AS. (B) Comparison between planarian and human stem cell regulated AS (Refers to Figure 3—source data 1) DOI: http://dx.doi.org/10.7554/eLife.16797.013 [file elife-16797-fig3-data2.docx]

**Supplementary File 1: conservation of stem cell-differential AS events**

**A) Comparison between *S. mediterranea* and *D. japonica* AS**

**
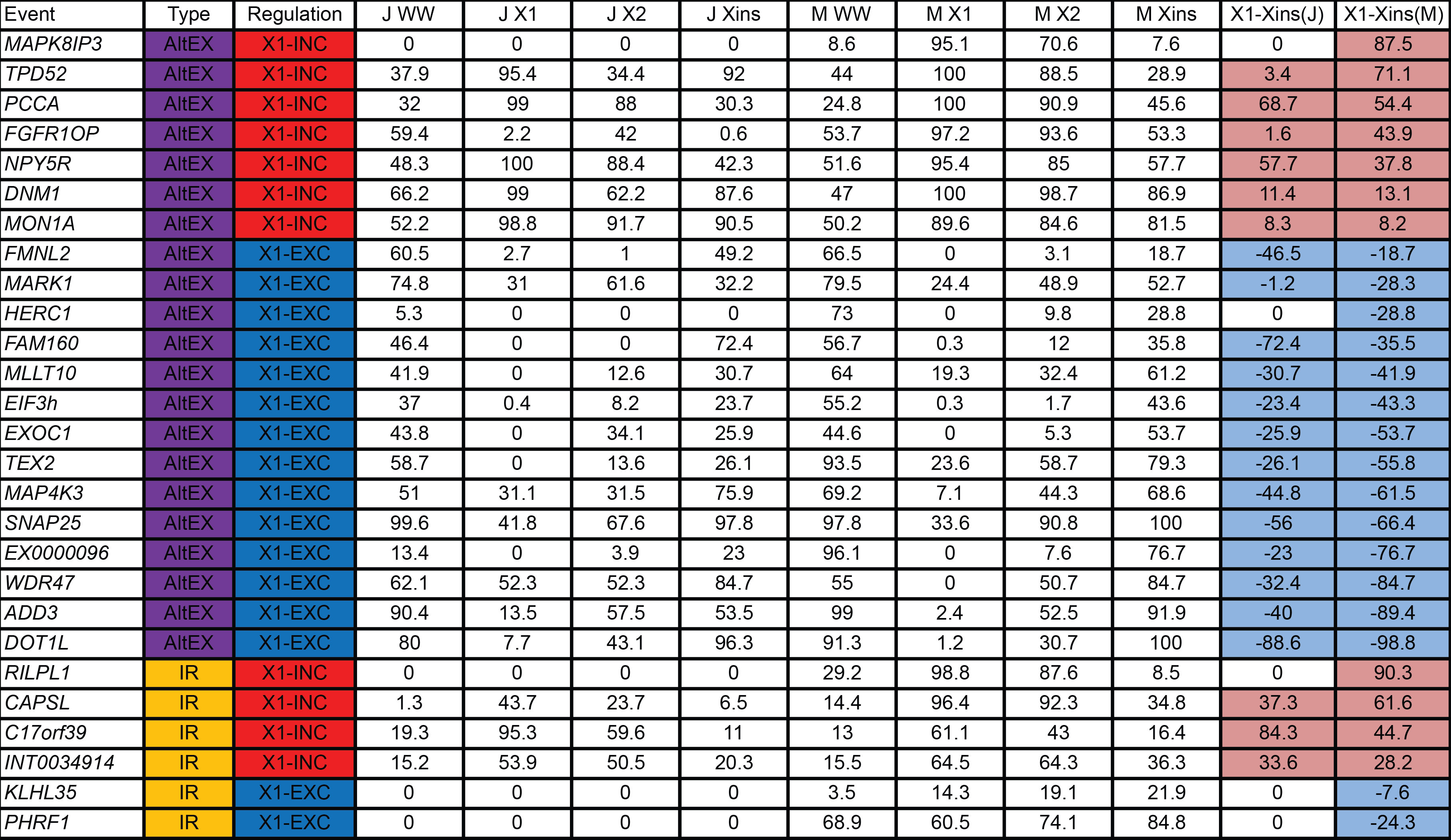
**

**Table 1 – Quantification by RT-PCR of inclusion levels for 27 neoblast-differential cassette exons (AltEX) and intron retention events (IR) in *S. mediterranea* (M) and *D. japonica* (J).**

**B) Comparison between planarian and human stem cell regulated AS (Refers to Figure 3-Source data 1)**

**General legend:**

- Sme/HsaEX#######: planarian or human exon Event_ID.

- A diagram with the position of relevant exons and predicted protein domains for all orthologous members is included at the beginning of each orthologous group. Exons are highlighted as red (neoblast-included) or blue (neoblast-excluded) bars above each protein. Domains are highlighted as colored blocks. Predictions of intrinsically disordered regions are shown as grey bars under each protein.

- For each group, protein sequences are also included in FASTA format. Protein domains are highlighted with specific font colors. Exons of interest correspond to regions with highlighted background.

- Protein headings include intron positions and phase.

e.g. >protein1 12.1 103.2

Intron in phase 1 in position 12 and in phase 2 in position 103.

- These intron positions are represented in the global intron/exon alignment, when necessary.

**ORTHOLOGOUS GROUP #1: *Adducin (ADD)***


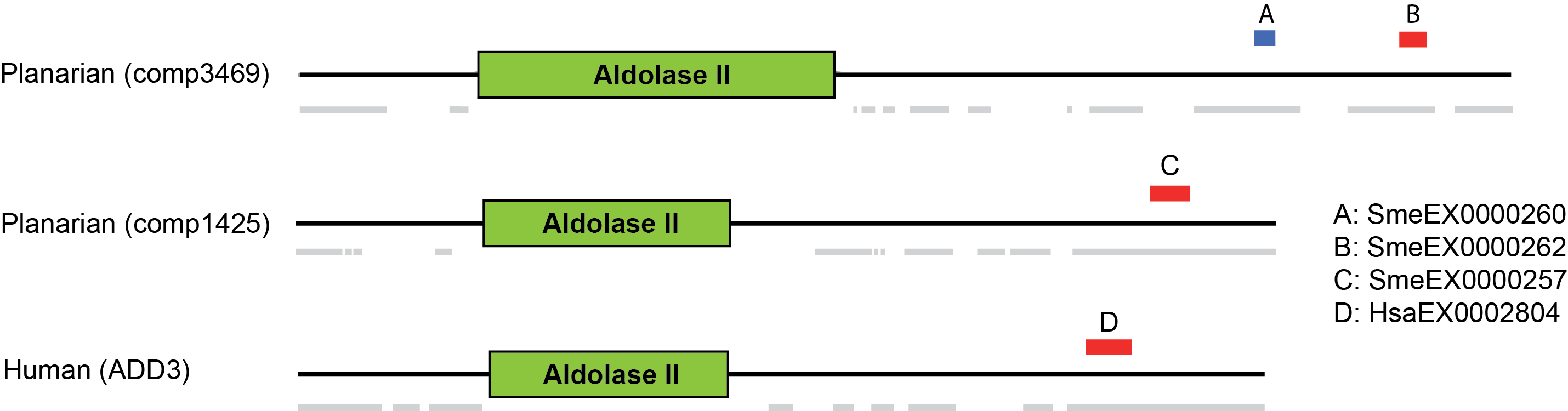


**- AS Exon key:**

SmeEX0000257

SmeEX0000262

SmeEX0000260

HsaEX0002804

HsaEX0002792

**- Protein Domain key:**

**Aldolase_II super family**

**- Planarian proteins:**

>comp3469_c0_seq1 555.0 625.0 702.1 711.1 806.1 824.1

MSTALFTDLSSYFNNIDPEDPVYQRELLRPAEIKEDVKLMEQRKRVSNIMRSKLFKKDLENIFLKYQFDDDPGISTSNLSLADISEQLLLSAIPKNKVANFSQLTNRKGLISINDLNSSISSLYGK**TERLSRCKLASIFRLINMYGWNSNFNSVMTNRPFSDKDQFLINPFGLLFHEITASSLISVDYKGNILDFGSTVLEANPLAWMIHSAIYSCQHSIRAILELKTPAALAISSLEHGLLPICREAILLGSTVILDNLPGFVSESKSISLIMTDLNNYLTSVIVPNSKILIVPNCGFLICGEHIEEAWHLLMNCQIACETQMKLGNLTIDDLYIPDKNCQADLFHQWRSTDLGGVTKIEIDSVPYENSEHVPQAQWRIGELEFEALMRHMDNNGYRT**GYMYRQENIYQSEKPLKPVVDKVKYEVEIPPSSISTIDQIKSADNADENRANKSFMRVKTEKWFNTPNTYKLIEDNDGIVDNNLSFNFPSVQSDRKSARWASAKAPTLSSSIPQHPVTAGAYVAIGHHHSSKSDSNKDKNISKINPKELLENQKRVKNKYYYDT*NTAGPISCLL*DSNISREEARMIKNESLKKHLNPSSVSANDQVVTSASKGIIQRGHKALVYQDLFKSGNPFDVTADELAAYHNEVDRKLHPERYKSVEIPQKDKYKEENPEIKITISQNESSLPSPENEVGDTSSRRDTGLTSDSDLPEFPNQKESSSNCVIIPTNSVECHPFFQSDFDEDFNNLKLIDSACNSTPLNEKCDGGDVNIKESPVVPSNLSGDNTKLKDEEDALKSATMKESKRKSFRSFSFKRLGKSKSLHESADILENKSATLAAPGYGTITSETSGVDISNDELDKDNKSKKKKRKFKLPSFSKKKKDKSKQ

>comp1425_c0_seq1 17.1 110.1 194.1 235.0 496.0 627.1 650.1 685.0

MPSVEGKKSDENYDLYDTENEEYQRQLLRPAEIKEDVKLMEQRQRVSRILHSEAFHRELNKILDQQFDEGATCGGLAGSTSESNAAAALQEISNFLHPQARLGCSVFNRGATMPIIPICDIKITDK**DVSKPEKLLRCKLASVYRLIDFHGWASGMMLHASVRINRESNEFLTNCHGLMNHEITASNLVKVNLKGEVLNAHSRIDLASWVLHSSIHQSDPRFKCVIHVRTPAVVAVSCMRSGLIKINEEAVKLGNVPIYEWGSASVNVGPGERLSVEQSTLTEAIGHRKCILVRNQGLLARGKTVEEAWFYAGLAVHACETQVRLASSVGSVNLLLANEEEREPEIEDQTIELQFESLMRTLD**AAGHRTGYLYRHAPIVVDRKRRLHEQQQQQEDGHMADMEDDDEDVEIPPSSTSFATNDEPAKPRSTQTQWLLHMPNDYVREEITENGTRKAVSWRPVSAESKQTSGLVETCTSPNQFAPQGRDKHELKHQQQKVKGKYFRDETSAGHQSRVLRNLDQTNEVEVVPEEDSVGRVVLVGAVSRGIIDKKHRHDPSVYQSCFSPNPFDRVTDDDLKQYKRKVLGLPSDETEVSKKEISSPEHSVAEPSVSSPPIDKPAPVVPVSWVEEKKEEIIVPAPIQQEMHAEPAPAATQQQQTLTVAAAKGETIRSDTSGADLSHDDTEREGHKSGSGAKKKKKKLKEMFSFRKSKDKSK

**- Human proteins:**

>ADD3_ENSP00000348381 66.0 112.1 163.0 190.0 240.0 288.0 321.0 382.0 468.0 508.0 537.0 578.1 610.1

MSSDASQGVITTPPPPSMPHKERYFDRINENDPEYIRERNMSPDLRQDFNMMEQRKRVTQILQSPAFREDLECLIQEQMKKGHNPTGLLALQQIADYIMANSFSGFSSPPLSLGMVTPINDLPGADTSSYVKGEKL**TRCKLASLYRLVDLFGWAHLANTYISVRISKEQDHIIIIPRGLSFSEATASNLVKVNIIGEVVDQGSTNLKIDHTGFSPHAAIYSTRPDVKCVIHIHTLATAAVSSMKCGILPISQESLLLGDVAYYDYQGSLEEQEERIQLQKVLGPSCKVLVLRNHGVVALGETLEEAFHYIFNVQLACEIQVQALAGAGGVDNLHVLDFQKYKAFTYTVAASGGGGVNMGSHQKWKVGEIEFEGLMRTLDNLGYRT**GYAYRHPLIREKPRHKSDVEIPATVTAFSFEDDTVPLSPLKYMAQRQQREKTRWLNSPNTYMKVNVPEESRNGETSPRTKITWMKAEDSSKVSGGTPIKIEDPNQFVPLNTNPNEVLEKRNKIREQNRYDLKTAGPQSQLLAGIVVDKPPSTMQFEDDDHGPPAPPNPFSHLTEGELEEYKRTIERKQQGLEDAEQELLSDDASSVSQIQSQTQSPQNVPEKLEENHELFSKSFISMEVPVMVVNGKDDMHDVEDELAKRVSRLSTSTTIENIEITIKSPEKIEEVLSPEGSPSKSPSKKKKKFRTPSFLKKNKKKEKVEA*

Sme1: comp3469_c0_seq1

Sme2: comp1425_c0_seq1

Hsa1: ADD3_ENSP00000348381

Sme1 ------------MSTALFTDLSSYFNNID * PEDPVYQRELLRPAEIKEDVKLMEQRKRVSNIMRSKL * FKKDLENIFLKYQFDDDP---GISTSNLSLADISEQLLLSAIPKNKVANFS * QLTNR- * --KGL Sme1

Sme2 ------------MPSVEGKKSDENYDLYD 1 TENEEYQRQLLRPAEIKEDVKLMEQRQRVSRILHSEA * FHRELN-KILDQQFDEGATCGGLAGSTSESNAAAALQEISNFLHPQARLGC * SVFNRG 1 ATMPI Sme2

Hsa1 MSSDASQGVITTPPPPSMPHKERYFDRIN * ENDPEYIRERNMSPDLRQDFNMMEQRKRVTQILQSPA 0 FREDLECLIQEQMKKGHN-----PTGLLALQQIADYIMANSFSGFSSPPLS 1 LGMVT- * ----- Hsa1

Sme1 ISINDLNSSISSLYGKTERLSRCKLASIFRLINMYGWNSNFNSVMTN * RPFSDKDQFLINPFGLLFHEITASSLI * SVDYKG * NILDFGSTVLEANPLAWMIHSAIYSCQHSIRAILELKTPAALAI * SSLE Sme1

Sme2 IPICDIKITDKDVS-KPEKLLRCKLASVYRLIDFHGWASGMMLHASV * RINRESNEFLTNCHGLMNHEITASNLV * KVNLKG 1 EVLNAHSRIDLAS---WVLHSSIHQSDPRFKCVIHVRTPAVVAV 0 SCMR Sme2

Hsa1 -PINDLPGADTSSYVKGEKLTRCKLASLYRLVDLFGWAHLANTYISV 0 RISKEQDHIIIIPRGLSFSEATASNLV 0 KVNIIG * EVVDQGSTNLKIDHTGFSPHAAIYSTRPDVKCVIHIHTLATAAV 0 SSMK Hsa1

Sme1 HGLLPICREAILLGSTVILDNLPGFVSESKSISLIMTDLNNYLTSVIVPNSKI * LIVPNCGFLICGEHIEEAWHLLMNCQIACETQM * KLGNLTIDDLYIPDKNCQADLFHQWRSTDLGGVTKIEIDSVPYENSEH Sme1

Sme2 SGLIKINEEAVKLGNVPIYEWGSASVNVGPGERLSVEQST---LTEAIGHRKC * ILVRNQGLLARGKTVEEAWFYAGLAVHACETQV * RLASSVG-----------------------------SVNLLLANEEER Sme2

Hsa1 CGILPISQESLLLGDVAYYDYQGSLEEQEERIQLQKVLGP---------SCKV 0 LVLRNHGVVALGETLEEAFHYIFNVQLACEIQV 0 QALAGAG-----------GVDNLHVLDFQKYKAFTYTVAASGGGGVNM Hsa1

Sme1 VPQAQWRIGELEFEALMRHMDNNG * YRTGYMYRQENIYQSEKPLKPVVDKVKYEVEIPPSSISTIDQIKSADNADENRANKSFMRVKTEKWFNTPNTYKLIEDNDGIVDNNLSFNFPSVQSDRKSARW * ASAKAPT Sme1

Sme2 EPEIEDQTIELQFESLMRTLDAAG * HRTGYLYRHAPIVVDRK-------RRLHEQQQQQEDGHMADMEDDDEDVEIPPSSTSFATNDEPAKPRSTQTQWLLHMPNDYVREEITENG-----TRKAVSW * RPVSAES Sme2

Hsa1 GSHQKWKVGEIEFEGLMRTLDNLG 0 YRTGYAYRHPLIREKPR--------HKSDVEIP-ATVTAFSFEDDTVPLSPLKYMAQRQQREKTRWLNSPNTYMKVNVPEESRNGET--------SPRTKITW 0 MKAEDSS Hsa1

Sme1 LSSSIPQHPVTAGAYVAIGHHHSSKSDSNKDKNISKINPKELLENQKRV 0 KNKYYYDTNTAGPISCLLDSNISREEARM * IKNESLKKHLNPSSVSANDQVVTSASKGIIQRG--HKALVYQD 0 LFKSGNPFDV Sme1

Sme2 KQTSGLVETCTS---------PNQFAPQGRDK-------HELKHQQQKV 0 KGKYFRDETSAGHQSRVLRNLDQTNEVEV * VPEE---------DSVGRVVLVGAVSRGIIDKKHRHDPSVYQS * CFSPNPFDRV Sme2

Hsa1 KVSGGTPIKIED---------PNQFVPLNTNP-------NEVLEKRNKI 0 REQNRYDLKTAGPQSQLLAGIVVDKPPST 0 MQFE---------------------------------DDDHGP * PAPPNPFSHL Hsa1

Sme1 TADELAAYHNEVDRKLHPERY * KSVEIPQKDKYKEENPEIKITISQNESSLPSPENEVGDTSSRRDTG 1 LTSDSDLPE 1 FPNQKESSSNCVIIPTNSVE * C * HPFFQSDFDEDFNNLKLIDSACNSTPLN Sme1

Sme2 TDDDLKQYK----RKVLGLPS * DETEVSKKEISSPEHSVAEPSVSSPPIDKPAP-------------- * --------- * ------------VVPVSWVE * E 1 K--------------------------- Sme2

Hsa1 TEGELEEYKRTIERKQQGLED 1 AEQELLSDDASSVSQIQSQTQSPQN--------------------- * --------- * -------------VPEKLEE 1 N * HELFSKSFIS------------------ Hsa1

Sme1 EKCDGGDVNIKESPVVPSNLSGDNTKLKDEEDALKSATMKESKRKS 1 FRSFSFKRLGKSKSLHES 1 AD * ILENKSATLAAPGYGTITSETSGVDISNDELDKDN * K-----SKKKKRKFKLPSFSKKKKDKS Sme1

Sme2 ----------KEEIIVPAPIQQEMHAEP------------------ * ----------------AP * AA 1 TQQQQTLTVAAAKGETIRSDTSGADLSHDDTEREG 0 HKSGSGAKKKKKKLKEMFSFRKSKDKS Sme2

Hsa1 ----------MEVPVMVVNGKDDMHDVED----------------- * ---------------ELA * KR * VSRLSTSTTIENIEITIKSPEKIEEVLSPEGSPSK * S-----PSKKKKKFRTPSFLKKNKKKE Hsa1

**ORTHOLOGOUS GROUP #2: *Echinoderm microtubule associated protein like(EML)***


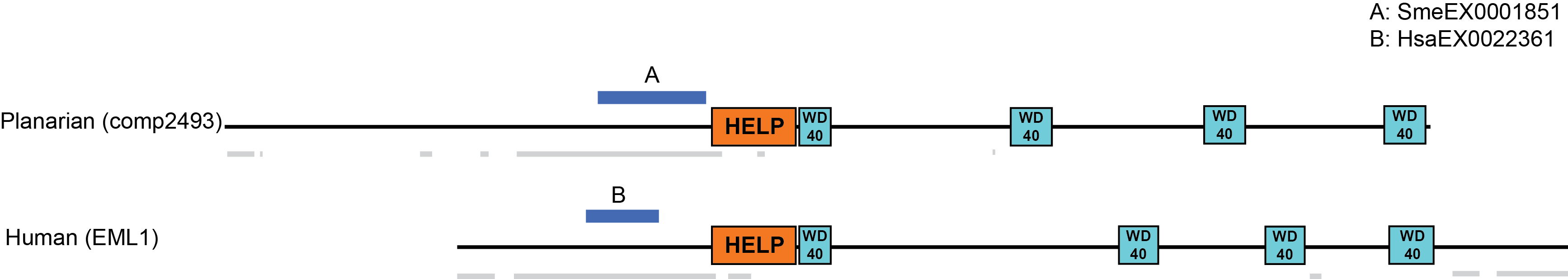


**- AS Exon key:**

SmeEX0001851

HsaEX0022361

Orthologous conserved constitutive exon

**- Protein Domain key:**

**TNG2 (broken in Sme; not detected in Hsa)**

**HELP**

**- Planarian proteins:**

>c2493_c0_seq1 160.1 264.1 332.2 421.2 430.1 472.2 522.2 547.0 581.0 613.0 658.0 691.1 774.0 887.1 957.0 1025.0

MSKNLMVISQKVFSPASFIARKFNKKKHRKHQLGVDLEVVQEKSQLSGYTSTDADANSLADADFSWRAEHGDHSPTVESVVDCPLIDELEASTQHVVDFSFPGSLNRETTLLGSSA**DMMEVQPETNSHNDIIDPFIKGVTVKAVECHVALRASNDLLTKNQDQELTETYALREKVSELEKIVSQQADEIACL*KSAMADNLRR*LKLLEDSRSKKNKTVNSMLTISIDENDNDPLLSPNSYSNVTNYSLSKSMVHLSTQASPRSSVSNSVALLNTNSLVPNSRRSTGQRTKRYPDVTPVRTTNSIGRFSSGKHSSARQSANSS**TPSINHIGLNKMSKSVTKSFTIPLSATKTKLTLPKSIDKKKLKKSQLMSNPWRPAGTPNPEFPVVEKLEPVKHNKEYLKVKAQSPPPVSSVSYRYRLNTGIKETIYNKDE**GSLKIYIKGRPIILSVPTYAQPIDLELLAPAAHETL*KLEWVYGYRG*RDSRNNVYFLPTGEIVYFIAAVVILYNME**EQTQRHYLGHTDDVKSIAIHPDKITIATGQVAGHSKNQGKPHIRIWSSVNLNTLHVVGLGDFERSICCLSFSKADGGMLICAVDEANEHVLSIWQWEKERRIVEAKSSTEPILSVDFHPLNNKSIITCGKNHLTFWNFEDNQLSKKQAVYEKNEKPKFILCQAFLDNGELVTGDSSGNIILWNMETHKVEKIVTTAHEGPIFSLLLTKNSTLLSGGGKDRKIKEWSSLLACIGCEKTLPETHGAIRMLSQGPGGMILVGSTKSSILQFHLQSNSQSMEFRSIVDGHVEEMWGLATHPKQHRFVTVGYDKLLCYWDSLTRTLIWRKEFTENLHSVCFHPLFEVIIIGCCAGRWMVIDTITREMVGVHTDGNEKIECVQFSTDGRSLAIGSRDNSIYVYNVSDNGYKYSRNGKCVGHSSFITHIDWSTNGQFLRSNSGDYEVLYWNSSTCRQVQSNDQIRDIDWATVNCTIDWSTAGIWSEGMDGTDINAVDKGHHLPVLCSADDFGRVNLYSYPFPNSKVPHHQFRGHSSHVTNVKFLFDDSRVISSGGKDSSIMQWEVL*

**- Human proteins:**

>EML4_ENSP00000320663 9.1 70.1 113.2 171.2 214.2 223.1 264.2 314.2 338.0 375.0 407.0 452.0 497.1 548.0 590.0 634.0 656.2 686.1 719.0 748.1 781.1 825.0

MDGFAGSLDDSISAASTSDVQDRLSALESRVQQQEDEITVLKAALADVLRRLAISEDHVASVKKSVSSKGQPSPRAVIPMSCITNGSGANRKPSHTSAVSIAGKETLSSAAKSGTEKKKEKPQGQREKKEESHSNDQSPQIRASPSPQPSSQPLQIHRQTPESKNATPTKSIKRPSPAEKSHNSWENSDDSRNKLSKIPSTPKLIPKVTKTADKHKDVIINQE**GEYIKMFMRGRPITMFIPSDVDNYDDIRTELPPEKLKLEWAYGYRGKDCRANVYLLPTGKIVYFIASVVVLFNYE**ERTQRHYLGHTDCVKCLAIHPDKIRIATGQIAGVDKDGRPLQPHVRVWDSVTLSTLQIIGLGTFERGVGCLDFSKADSGVHLCIIDDSNEHMLTVWDWQKKAKGAEIKTTNEVVLAVEFHPTDANTIITCGKSHIFFWTWSGNSLTRKQGIFGKYEKPKFVQCLAFLGNGDVLTGDSGGVMLIWSKTTVEPTPGKGPKGVYQISKQIKAHDGSVFTLCQMRNGMLLTGGGKDRKIILWDHDLNPEREIEVPDQYGTIRAVAEGKADQFLVGTSRNFILRGTFNDGFQIEVQGHTDELWGLATHPFKDLLLTCAQDRQVCLWNSMEHRLEWTRLVDEPGHCADFHPSGTVVAIGTHSGRWFVLDAETRDLVSIHTDGNEQLSVMRYSIDGTFLAVGSHDNFIYLYVVSENGRKYSRYGRCTGHSSYITHLDWSPDNKYIMSNSGDYEILYWDIPNGCKLIRNRSDCKDIDWTTYTCVLGFQVFGVWPEGSDGTDINALVRSHNRKVIAVADDFCKVHLFQYPCSKAKAPSHKYSAHSSHVTNVSFTHNDSHLISTGGKDMSIIQWKLVEKLSLPQNETVADTTLTKAPVSSTESVIQSNTPTPPPSQPLNETAEEESRISSSPTLLENSLEQTVEPSEDHSEEESEEGSGDLGEPLYEEPCNEISKEQAKATLLEDQQDPSPSS*

Sme1I: c2493_c0_seq1 (inclusion form)

Sme1E: c2493_c0_seq1 (exclusion form)

Hsa1I: EML4_ENSP00000320663 (inclusion form)

Hsa1E: EML4_ENSP00000385059 (exclusion form)

Sme1I GVTVKAVECHVALRASNDLLTKN 1 QDQELTETYALREKVSELEKIVSQQADEIACLKSAMADNLRRLKLLEDSRSKKNKTVNSML * TISIDENDNDPLLSPNSYSNVTNYSLSKSMVHLSTQASPRSSV 1 SNSV Sme1I

Sme1E GVTVKAVECHVALRASNDLLTKN 1 QDQELTETYALREKVSELEKIVSQQADEIACLKSAMADNLRRLKLLEDSRSKKNKTVNSML * TISIDENDNDPLLSPNSYSNVTNYSLSKSMVHLSTQASPRSSV 1 SNSV Sme1E

Hsa1I ----------------------- * -------TSDVQDRLSALESRVQQQEDEITVLKAALADVLRRLAISEDHVASVKKSVSSKG 1 QPSP------------------RAVIPMSCITNGSGANRKPSH * TSAV Hsa1I

Hsa1E ----------------------- * -------TSDVQDRLSALESRVQQQEDEITVLKAALADVLRRLAISEDHVASVKKSVSSKG 1 QPSP------------------RAVIPMSCITNGSGANRKPSH * TSAV Hsa1E

Sme1I ALLNTNSLVPNSRR * STGQRTKRYPDVTPVRTTNSIGRFSSGKHSSARQSANSSTPSINHIGLNK 2 MSKSVTKS * FTIPLSATKTKLTLPKSIDKKKLKKSQLMSNPWRPAGTPNPEFPVVEKLEPVKHNKEYL Sme1I

Sme1E ALLNTNSLVPNSRR * STGQRTKRYPDVTPVRTTNSIGRFSSGKHSSARQSANSSTPSINHIGLNK 2 MSKSVTKS * FTIPLSATKTKLTLPKSIDKKKLKKSQLMSNPWRPAGTPNPEFPVVEKLEPVKHNKEYL Sme1E

Hsa1I SIAGKETLSSAAKS 2 GTEKKKEKPQGQREKKEESHSNDQSPQIRASPSPQPSSQPLQIHRQTPES * KNATPTKS 2 IKRPSPAEKSHNSWENSDDSR-NKLSKIPSTP--------------------------- Hsa1I

Hsa1E SIAGKETLSSAAKS 2 -------------------------------------------------- * -------- * IKRPSPAEKSHNSWENSDDSR-NKLSKIPSTP--------------------------- Hsa1E

Sme1I KVKAQSPPPVSSVSYRYRLNTG 2 IKETIYNKD 1 **EGSLKIYIKGRPIILSVPTYAQPIDLELLAPAAHETLKLEWV 2 YGYRGRDSRNNVYFLPTGEIVYFIAAVVILYNMEEQTQRHYLGHTDDVKS 2 IA**  Sme1I

Sme1E KVKAQSPPPVSSVSYRYRLNTG 2 IKETIYNKD 1 **EGSLKIYIKGRPIILSVPTYAQPIDLELLAPAAHETLKLEWV 2 YGYRGRDSRNNVYFLPTGEIVYFIAAVVILYNMEEQTQRHYLGHTDDVKS 2 IA**  Sme1E

Hsa1I ----------KLIPKVTKTADK 2 HKDVIINQE 1 **GEYIKMFMRGRPITMFIPSDVDNYD-DIRTELPPEKLKLEWA 2 YGYRGKDCRANVYLLPTGKIVYFIASVVVLFNYEERTQRHYLGHTDCVKC 2 LA**  Hsa1I

Hsa1E ----------KLIPKVTKTADK 2 HKDVIINQE 1 **GEYIKMFMRGRPITMFIPSDVDNYD-DIRTELPPEKLKLEWA 2 YGYRGKDCRANVYLLPTGKIVYFIASVVVLFNYEERTQRHYLGHTDCVKC 2 LA**  Hsa1E

Sme1I IHPDKITIATGQVAGHSKNQGK * - * ------------------P 0 HIRIWSSVNLNTLHVVGLGDFERSICCLSFSKAD 0 GGMLICAVDEANEHVLSIWQWEKERRIVEAKS 0 STEPILSVDFHPLNNKS Sme1I

Sme1E IHPDKITIATGQVAGHSKNQGK * H 0 KSTRFKYRSDLSSSIPEWP 0 HIRIWSSVNLNTLHVVGLGDFERSICCLSFSKAD 0 GGMLICAVDEANEHVLSIWQWEKERRIVEAKS 0 STEPILSVDFHPLNNKS Sme1E

Hsa1I IHPDKIRIATGQIAGVDKDGRP 0 - * ----------------LQP * HVRVWDSVTLSTLQIIGLGTFERGVGCLDFSKAD 0 SGVHLCIIDDSNEHMLTVWDWQKKAKGAEIKT 0 TNEVVLAVEFHPTDANT Hsa1I

Hsa1E IHPDKIRIATGQIAGVDKDGRP 0 - * ----------------LQP * HVRVWDSVTLSTLQIIGLGTFERGVGCLDFSKAD 0 SGVHLCIIDDSNEHMLTVWDWQKKAKGAEIKT 0 TNEVVLAVEFHPTDANT Hsa1E

Sme1I IITCGKNHLTFWNFEDNQLSKKQAVYEK 0 NEKPKFILCQAFLDNGELVTGDSSGNIILWNME 1 THKVEK------ * -----IVTTAHEGPIFSLLLTKNSTLLSGGGKDRKIKEWSSLLACIGCEKTL * PET Sme1I

Sme1E IITCGKNHLTFWNFEDNQLSKKQAVYEK 0 NEKPKFILCQAFLDNGELVTGDSSGNIILWNME 1 THKVEK------ * -----IVTTAHEGPIFSLLLTKNSTLLSGGGKDRKIKEWSSLLACIGCEKTL * PET Sme1E

Hsa1I IITCGKSHIFFWTWSGNSLTRKQGIFGK 0 YEKPKFVQCLAFLGNGDVLTGDSGGVMLIWSKT * TVEPTPGKGPKG 1 VYQISKQIKAHDGSVFTLCQMRNGMLLTGGGKDRKIILWDHDLNPE-REIEV 0 PDQ Hsa1I

Hsa1E IITCGKSHIFFWTWSGNSLTRKQGIFGK 0 YEKPKFVQCLAFLGNGDVLTGDSGGVMLIWSKT * TVEPTPGKGPKG 1 VYQISKQIKAHDGSVFTLCQMRNGMLLTGGGKDRKIILWDHDLNPE-REIEV 0 PDQ Hsa1E

Sme1I HGAIRMLSQGPGGMILVGSTKSSILQF 0 HLQSNSQSMEFRSIVDG * HVEEMWGLATHPKQHRFVTVGYDKLLCYWDSLTRTLIWRKEFTE * NLHSVCFHPLFEVIIIGCCAGR * WMVIDTITREMVGVHTDG Sme1I

Sme1E HGAIRMLSQGPGGMILVGSTKSSILQF 0 HLQSNSQSMEFRSIVDG * HVEEMWGLATHPKQHRFVTVGYDKLLCYWDSLTRTLIWRKEFTE * NLHSVCFHPLFEVIIIGCCAGR * WMVIDTITREMVGVHTDG Sme1E

Hsa1I YGTIRAVAEGKADQFLVGTSRNFILRG * TFNDG-----FQIEVQG 0 HTDELWGLATHPFKDLLLTCAQDRQVCLWNSMEHRLEWTRLVDE 0 PGHCADFHPSGTVVAIGTHSGR 2 WFVLDAETRDLVSIHTDG Hsa1I

Hsa1E YGTIRAVAEGKADQFLVGTSRNFILRG * TFNDG-----FQIEVQG 0 HTDELWGLATHPFKDLLLTCAQDRQVCLWNSMEHRLEWTRLVDE 0 PGHCADFHPSGTVVAIGTHSGR 2 WFVLDAETRDLVSIHTDG Hsa1E

Sme1I NEKIECVQFSTD 1 GRSLAIGSRDNSIYVYNVSDNGYKYSRNGKCVG * HSSFITHIDWSTNGQFLRSNSGDYEVLYW * NSST-CRQV 0 QSNDQIRDIDWATVNCTIDWSTAG * IWSEGMDGTDINAVDKGH Sme1I

Sme1E NEKIECVQFSTD 1 GRSLAIGSRDNSIYVYNVSDNGYKYSRNGKCVG * HSSFITHIDWSTNGQFLRSNSGDYEVLYW * NSST-CRQV 0 QSNDQIRDIDWATVNCTIDWSTAG * IWSEGMDGTDINAVDKGH Sme1E

Hsa1I NEQLSVMRYSID 1 GTFLAVGSHDNFIYLYVVSENGRKYSRYGRCTG 0 HSSYITHLDWSPDNKYIMSNSGDYEILYW 1 DIPNGCKLI * RNRSDCKDIDWTTYTCVLGFQVFG 1 VWPEGSDGTDINALVRSH Hsa1I

Hsa1E NEQLSVMRYSID 1 GTFLAVGSHDNFIYLYVVSENGRKYSRYGRCTG 0 HSSYITHLDWSPDNKYIMSNSGDYEILYW 1 DIPNGCKLI * RNRSDCKDIDWTTYTCVLGFQVFG 1 VWPEGSDGTDINALVRSH Hsa1E

Sme1I HLPVLCSADDFGRVNLYSYPFPNSKV 0 PHHQFRGHSSHVTNVKFLFDDSRVISSGGKDSSIMQWEVL----------------------------------------------------------------------- Sme1I

Sme1E HLPVLCSADDFGRVNLYSYPFPNSKV 0 PHHQFRGHSSHVTNVKFLFDDSRVISSGGKDSSIMQWEVL----------------------------------------------------------------------- Sme1E

Hsa1I NRKVIAVADDFCKVHLFQYPCSKAKA 0 PSHKYSAHSSHVTNVSFTHNDSHLISTGGKDMSIIQWKLVEKLSLPQNETVADTTLTKAPVSSTESVIQSNTPTPPPSQPLNETAEEESRISSSPTLLENSLEQTVEPSED Hsa1I

Hsa1E NRKVIAVADDFCKVHLFQYPCSKAKA 0 PSHKYSAHSSHVTNVSFTHNDSHLISTGGKDMSIIQWKLVEKLSLPQNETVADTTLTKAPVSSTESVIQSNTPTPPPSQPLNETAEEESRISSSPTLLENSLEQTVEPSED Hsa1E

**ORTHOLOGOUS GROUP #3: *Exocyst complex component (EXOC)***


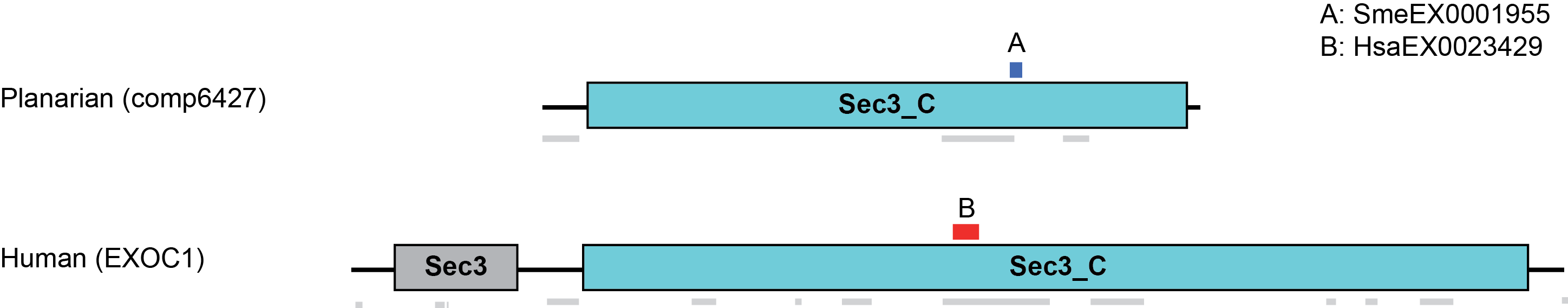


**- AS Exon key:**

SmeEX0001955

HsaEX0023429

**- Protein Domain key:**

**Sec3_C**

**Sme C1 exon**

**- Planarian proteins:**

>comp6427_c0_seq1 105.0 269.0 300.1 347.0 353.0 386.0

FETDKPDNDTECVDVNDGGRYQNITKAEEENMQKFLAESSEAVI**NAEKFQDTLTHHLLAMEGTNVHSIMSSENQMLKLMECLDQAIEQVELLENKIDIQDKILAEVEESMSCLRDRDKLIRITALNRDKLLESLESLLYRISLEDKYFVALKNLDLRNTESVAVARAAVEKLHAIFNTSPIDDLNSMRVVKEKTAQLNKAADEFARRFAAQFNNILTFQVNKLTTISTSNIEKILDLHRTNRKELISAADFMAWLKEDRPRTFTDLQKLYIEKMSTVYNHEIADMIDLIKGKLISKEKNVSYSTSKTGSHLNLKISEKIRGRQISESCTSLQRESSKMSTGGIFDIKTAGLVIFEDFLHKLKLIVLEEEEFMTRLFRLDCRRSSERNEAVSNKLEPHEPHVYLISASTTVFSQLSDHLKSLIQILEQHNSSQSLLMVVIVDRQFDQSVRSSEFLQISWGELFIYTKRSFNAYIVNIFVLIPVLH***

**- Human proteins:**

>EXOC1_ENSP00000370695 42.1 86.0 139.1 202.0 278.0 322.1 359.0 409.0 444.1 459.1 514.0 550.0 575.2 652.0 713.1 780.0 845.0

MTAIKHALQRDIFTPNDERLLSIVNVCKAGKKKKNCFLCATVTTERPVQVKVVKVKKSDKGDFYKRQIAWALRDLAVVDAKDAIKENPEFDLHFEKIYKWVASSTAEKNAFISCIWKLNQRYLRKKIDFVNVSSQLLEESVPSGENQSVTGGDEEVVDEYQELNAREEQDIEIMMEGCEYAIS**NAEAFAEKLSRELQVLDGANIQSIMASEKQVNILMKLLDEALKEVDQIELKLSSYEEMLQSVKEQMDQISESNHLIHLSNTNNVKLLSEIEFLVNHMDLAKGHIKALQEGDLASSRGIEACTNAADALLQCMNVALRPGHDLLLAVKQQQQRFSDLRELFARRLASHLNNVFVQQGHDQSSTLAQHSVELTLPNHHPFHRDLLRYAKLMEWLKSTDYGKYEGLTKNYMDYLSRLYEREIKDFFEVAKIKMTGTTKESKKFATLPRKESAVKQETESLHGSSGKLTGSTSSLNKLSVQSSGNRRSQSSSLLDMGNMSASDLDVADR*TKFDKI*FEQVLSELEPLCLAEQDFISKFFKLQQHQSMPGTMAEAEDLDGGTLSRQHNCGTPLPVSSEKDMIRQMMIKIFRCIEPELNNLIALGDKIDSFNSLYMLVKMSHHVWTAQNVDPASFLSTTLGNVLVTVKRNFDKCISNQIRQMEEVKISKKSKVGILPFVAEFEEFAGLAESIFKNAERRGDLDKAYTKLIRGVFVNVEKVANESQKTPRDVVMMENFHHIFATLSRLKISCLEAEKKEAKQKYTDHLQSYVIYSLGQPLEKLNHFFEGVEARVAQGIREEEVSYQLAFNKQELRKVIKEYPGKEVKKGLDNLYKKVDKHLCEEENLLQVVWHSMQDEFIRQYKHFEGLIARCYPG**SGVTMEFTIQDILDYCSSIAQSH*

Hsa1I: ENSP00000370695 (inclusion form)

Hsa1E: ENSP00000334431 (exclusion form)

Sme1: comp6427_c0_seq1

Hsa1I MTAIKHALQRDIFTPNDERLLSIVNVCKAGKKKKNCFLCATV 1 TTERPVQVKVVKVKKSDKGDFYKRQIAWALRDLAVVDAKDAIKE 0 NPEFDLHFEKIYKWVASSTAEKNAFISCIWKLNQRYLRKKIDFVNVSS Hsa1I

Hsa1E MTAIKHALQRDIFTPNDERLLSIVNVCKAGKKKKNCFLCATV 1 TTERPVQVKVVKVKKSDKGDFYKRQIAWALRDLAVVDAKDAIKE 0 NPEFDLHFEKIYKWVASSTAEKNAFISCIWKLNQRYLRKKIDFVNVSS Hsa1E

Sme1 ------------------------------------------ * -------------------------------------------- * ------------------------------------------------ Sme1

Hsa1I QLLEE 1 SVPSGENQSVTGGDEEVVDEYQELNAREEQDIEIMMEGCEYAIS**NAEAFAEKLSRELQVLDGA 0 NIQSIMASEKQVNILMKLLDEALKEVDQIELKLSSYEEMLQS * VKEQMDQISESNHLIHLSNTN** Hsa1I

Hsa1E QLLEE 1 SVPSGENQSVTGGDEEVVDEYQELNAREEQDIEIMMEGCEYAIS**NAEAFAEKLSRELQVLDGA 0 NIQSIMASEKQVNILMKLLDEALKEVDQIELKLSSYEEMLQS * VKEQMDQISESNHLIHLSNTN** Hsa1E

Sme1 --FET * DKPDNDTECVDVNDG---GRYQNITKAEEENMQKFLAESSEAVI**NAEKFQDTLTHHLLAMEGT * NVHSIMSSENQMLKLMECLDQAIEQVELLENKIDIQDKILAE 0 VEESMSCLRDRDKLIRITALN** Sme1

Hsa1I **NVKLLSEIEFLVN 0 HMDLAKGHIKALQEGDLASSRGIEACTNAADALLQCMNVALRPG 1 HDLLLAVKQQQQRFSDLRELFARRLASHLNNVFVQQG 0 HDQSSTLAQHSVELTLPNHHPFHRDLLRYAKLMEWLK** Hsa1I

Hsa1E **NVKLLSEIEFLVN 0 HMDLAKGHIKALQEGDLASSRGIEACTNAADALLQCMNVALRPG 1 HDLLLAVKQQQQRFSDLRELFARRLASHLNNVFVQQG 0 HDQSSTLAQHSVELTLPNHHPFHRDLLRYAKLMEWLK** Hsa1E

Sme1 **RDKLLESLESLLY * RISLEDKYFVALKNLDLRNTESVAVARAAVEKLHAIFNTSPIDD * LNSMRVVKEKTAQLNKAADEFARRFAAQFNNILTFQV * N-KLTTISTSNIEKILDLHRTNRKELISAADFMAWLK** Sme1

Hsa1I **STDYGKYEGLTKN 0 YMDYLSRLYEREIKDFFEVAKIKMTGTTKESKKFA 1 TLPRKESAVKQETES 1 LHGSSGKLTGSTSSLNKLSVQSSGNRRSQSSSLLDMGNMSASDLDVADR * TKFDKI 0 FEQV** Hsa1I

Hsa1E **STDYGKYEGLTKN 0 YMDYLSRLYEREIKDFFEVAKIKMTGTTKESKKFG 1 --------------- * LHGSSGKLTGSTSSLNKLSVQSSGNRRSQSSSLLDMGNMSASDLDVADR * TKFDKI 0 FEQV** Hsa1E

Sme1 **EDRPRTFTDLQKL 0 YIEKMSTVYNHEIADMIDLIKGKLISKEKNV---- 1 --------------- * --SYSTSKTGSHLNLKISEKIRGRQISESCTSLQRESSKMSTGGIFDIK 0 TAGLVI 0 FEDF** Sme1

Hsa1I **LSELEPLCLAEQDFISKFFKLQQHQSMPGTMA 0 EAEDLDGGTLSR * QHNCGTPLPVSSE 2 KDMIRQMMIKIFRCIEPELNNLIALGDKIDSFNSLYMLVKMSHHVWTAQNVDPASFLSTTLGNVLVTVKRNFDK** Hsa1I

Hsa1E **LSELEPLCLAEQDFISKFFKLQQHQSMPGTMA 0 EAEDLDGGTLSR * QHNCGTPLPVSSE 2 KDMIRQMMIKIFRCIEPELNNLIALGDKIDSFNSLYMLVKMSHHVWTAQNVDPASFLSTTLGNVLVTVKRNFDK** Hsa1E

Sme1 **LHKLKLIVLEEEEFMTRLFRLDCRRSSE---- * -----------R 0 NEAVSNKLEPHEP * HVYLISASTTVFSQLSDHLKSLIQILEQHNSSQSLLMVVIVDR--QFDQSVRSSEFLQISWGELFIYTKRSFNA** Sme1

Hsa1I **CIS 0 NQIRQMEEVKISKKSKVGILPFVAEFEEFAGLAESIFKNAERRGDLDKAYTKLIRGVFVNV 1 EKVANESQKTPRDVVMMENFHHIFATLSRLKISCLEAEKKEAKQKYTDHLQSYVIYSLGQPLEKLNH 0** Hsa1I

Hsa1E **CIS 0 NQIRQMEEVKISKKSKVGILPFVAEFEEFAGLAESIFKNAERRGDLDKAYTKLIRGVFVNV 1 EKVANESQKTPRDVVMMENFHHIFATLSRLKISCLEAEKKEAKQKYTDHLQSYVIYSLGQPLEKLNH 0** Hsa1E

Sme1 **YIV * NIFVLIPVLH--------------------------------------------------- * ------------------------------------------------------------------- *** Sme1

Hsa1I **FFEGVEARVAQGIREEEVSYQLAFNKQELRKVIKEYPGKEVKKGLDNLYKKVDKHLCEEENLLQV 0 VWHSMQDEFIRQYKHFEGLIARCYPG**SGVTMEFTIQDILDYCSSIAQSH Hsa1I

Hsa1E **FFEGVEARVAQGIREEEVSYQLAFNKQELRKVIKEYPGKEVKKGLDNLYKKVDKHLCEEENLLQV 0 VWHSMQDEFIRQYKHFEGLIARCYPG**SGVTMEFTIQDILDYCSSIAQSH Hsa1E

Sme1 ----------------------------------------------------------------- * ------------------------------------------------- Sme1

**ORTHOLOGOUS GROUP #4: *Formin-like (FMNL)***


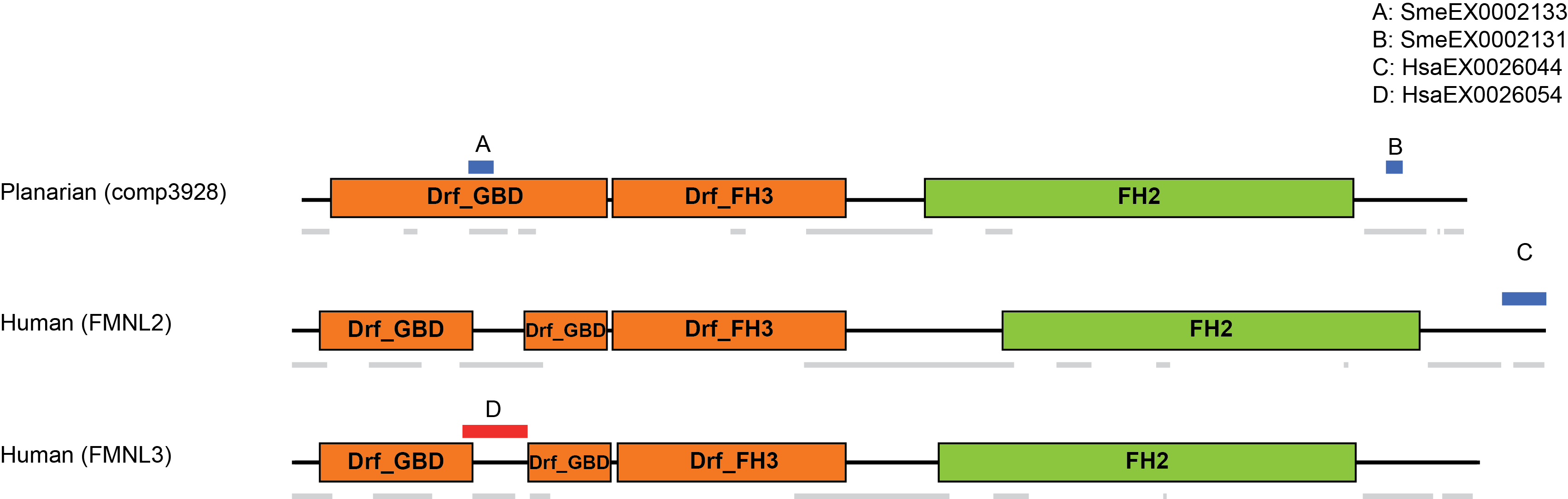


**- AS Exon key:**

SmeEX0002131

SmeEX0002133

HsaEX0026044

HsaEX0026054

**- Protein Domain key:**

**Rho GTPase-binding/formin homology 3 (GBD/FH3)**

**Formin homology-2 (FH2)**

**- Planarian proteins:**

>comp3928_c0_seq1 68.0 121.2 148.2 162.2 227.0 252.2 315.0 352.0 429.0 538.1 629.2 781.0 824.1 914.0 945.0 954.0 986.1

MGSTSSKSTPQYKFINSGEGRL**EMRPTNLQFEKRWDTFIQSLDVGPENVRQIDQLDPDQKEQLLLNFESKNPKYSAFHYVTLIKALQTGKSIRKSFNKKPDDDPVHVLSATEVSLRTNNVDWVYDFLNNNGLEVLLDYMSKTLVLFLNINYKNIETKKTLNLQSDTKTHLSSSDLFKSKKFNCCASIKRRSSVVTKFQNSKDSVNESVKNSLHQGLKCMRAILNNQRGCEMAFVHSRAIDVIALCLLHPNYQTKTLVLETLAAICLIVNGLQKVLAAFEYFKEITGESRRFDTLITYFRDHETFKVDDYNIDFMVSCTQFINIVVHSGEDINLRVYLQQEFTILGLDDFYRKLEERSGDRLKKQVAAYNGNRVDVAILLEDSETKETVLLDYEKLEHEFYANEEKYQTQIVHIENEKTELLQIIDSLKAQKEEQDKEYRTLREQISEPRNSTDREK**ELVLEINSLKKVLTEKNIPFNSSVLTELSPNKSSLPLASISPPPPPIAPPPPIAPPPSCAPPPPGQPPGVFKLGTGATNTQTQS**IRPPIQTKYKLPVFNWSVLKNQQLKGTVFVSMNDEVLYDKLEFEKFENIFKLNPVGDSIPNGVDSNDSSEKTSKRKPEKKTLLDGSKHRCLGVLLKFLDQEKLHSDILCKSINQLEVSVEVADRILYQLPTNDDIKTYRRYEYSDKLPINALTDEDRLLLTLCKVERLQQKLEVVVYIHNFPEAYKSLVEKVTFVTRASLAVRSSQKLKQILEIILAYGNLMNSSRRGIMYGFRLQSLDLLTDTKSLDKSWTLMQFFVDIVESKFQNLSDFSDEMRFVEQASKIPMEAITTDVASLVGGMKQVRDEMNCNTNQPPRLKKFFEDNENLTADLSKSAANAKETFLQAAEWFGERQNNPTPDVFFGIFHRFTENFKKSLEDVRKRRAQSIILNIVPTEETP**KAKPKDKNLSMEELQKKLALEARKKNRRMNNRTRQINADGIMDDILAGLDKTPLQAEIHPKRSNLRYQEEVF

**- Human proteins:**

>FMNL2_ENSP00000288670 40.0 68.0 95.0 120.2 148.2 199.2 236.0 261.2 293.0 318.0 355.0 405.0 439.0 543.1 613.1 655.0 722.2 801.0 824.0 851.0 894.1 949.0 983.0 1016.0 1057.1

MGNAGSMDSQQTDFRAHNVPLKLPMPEPGELEERFAIVLNAMNLPPDKARLLRQYDNEKKWELICDQERFQVKNPPHTYIQKLKGYLDPAVTRKKFRRRVQESTQVLRELEISLRTNHIGWVREFLNEENKGLDVLVEYLSFAQYAVTFDFESVESTVESSVDKSKPWSRSIEDLHRGSNLPSPVGNSVSRSGRHSALRYNTLPSRRTLKNSRLVSKKDDVHVCIMCLRAIMNYQYGFNMVMSHPHAVNEIALSLNNKNPRTKALVLELLAAVCLVRGGHEIILSAFDNFKEVCGEKQRFEKLMEHFRNEDNNIDFMVASMQFINIVVHSVEDMNFRVHLQYEFTKLGLDEYLDKLKHTESDKLQVQIQAYLDNVFDVGALLEDAETKNAALERVEELEENISHLSEKLQDTENEAMSKIVELEKQLMQRNKELDVVREIYKDANTQVHTLRKMVKEKEEAIQRQSTLEKKIHELEKQGTIKIQKKGDGDIAILPVVASGTLSMGSEVVAGNSVGPTMGAASSGPLPPPPPPLPPSSDTPETVQNGPVTPPMPPPPPPPPPPPPPPPPPPPPPLPGPAAETVPAPPLAPPLPSAPPLPGTSSPTVVFNSGLAAVK**IKKPIKTKFRMPVFNWVALKPNQINGTVFNEIDDERILEDLNVDEFEEIFKTKAQGPAIDLSSSKQKIPQKGSNKVTLLEANRAKNLAITLRKAGKTADEICKAIHVFDLKTLPVDFVECLMRFLPTENEVKVLRLYERERKPLENLSDEDRFMMQFSKIERLMQKMTIMAFIGNFAESIQMLTPQLHAIIAASVSIKSSQKLKKILEIILALGNYMNSSKRGAVYGFKLQSLDLLLDTKSTDRKQTLLHYISNVVKEKYHQVSLFYNELHYVEKAAAVSLENVLLDVKELQRGMDLTKREYTMHDHNTLLKEFILNNEGKLKKLQDDAKIAQDAFDDVVKYFGENPKTTPPSVFFPVFVRFVKAYKQAEEENELRKKQEQALMEKLLEQEA**LMEQQDPKSPSHKSKRQQQELIAELRRRQVKDNRHVYEGKDGAIEDIITDLRNQPYRRADAVRRSVRRRFDDQNLRSVNGAEITM

>FMNL2_ENSP00000288670f10936A 40.0 68.0 95.0 120.2 148.2 199.2 236.0 261.2 293.0 318.0 355.0 405.0 439.0 543.1 613.1 655.0 722.2 801.0 824.0 851.0 894.1 949.0 983.0 1016.0 1057.1 1088.2

MGNAGSMDSQQTDFRAHNVPLK**LPMPEPGELEERFAIVLNAMNLPPDKARLLRQYDNEKKWELICDQERFQVKNPPHTYIQKLKGYLDPAVTRKKFRRRVQESTQVLRELEISLRTNHIGWVREFLNEENKGLDVLVEYLSFAQYAVTFDFESVESTVESSVDKSKPWSRSIEDLHRGSNLPSPVGNSVSRSGRHSALRYNTLPSRRTLKNSRLVSKKDDVHVCIMCLRAIMNYQYGFNMVMSHPHAVNEIALSLNNKNPRTKALVLELLAAVCLVRGGHEIILSAFDNFKEVCGEKQRFEKLMEHFRNEDNNIDFMVASMQFINIVVHSVEDMNFRVHLQYEFTKLGLDEYLDKLKHTESDKLQVQIQAYLDNVFDVGALLEDAETKNAALERVEELEENISHLSEKLQDTENEAMSKIVELEKQLMQRNKELDVVREIYKDANTQVHTLRKMVKEKEEAIQRQSTLEKKIHE**LEKQGTIKIQKKGDGDIAILPVVASGTLSMGSEVVAGNSVGPTMGAASSGPLPPPPPPLPPSSDTPETVQNGPVTPPMPPPPPPPPPPPPPPPPPPPPPLPGPAAETVPAPPLAPPLPSAPPLPGTSSPTVVFNSGLAAVK**IKKPIKTKFRMPVFNWVALKPNQINGTVFNEIDDERILEDLNVDEFEEIFKTKAQGPAIDLSSSKQKIPQKGSNKVTLLEANRAKNLAITLRKAGKTADEICKAIHVFDLKTLPVDFVECLMRFLPTENEVKVLRLYERERKPLENLSDEDRFMMQFSKIERLMQKMTIMAFIGNFAESIQMLTPQLHAIIAASVSIKSSQKLKKILEIILALGNYMNSSKRGAVYGFKLQSLDLLLDTKSTDRKQTLLHYISNVVKEKYHQVSLFYNELHYVEKAAAVSLENVLLDVKELQRGMDLTKREYTMHDHNTLLKEFILNNEGKLKKLQDDAKIAQDAFDDVVKYFGENPKTTPPSVFFPVFVRFVKAYKQAEEENELRKKQEQALMEKLLEQEA**LMEQQDPKSPSHKSKRQQQELIAELRRRQVKDNRHVYEGKDGAIEDIITALKKNNITKFPNVHSRVRISSSTPVVEDTQS

>FMNL3_ENSP00000335655 43.0 71.0 98.0 123.2 151.2 202.2 239.0 264.2 296.0 321.0 358.0 408.0 442.0 535.1 558.1 600.0 667.2 746.0 769.0 796.0 839.1 893.0 927.0 958.0 999.1

MGNLESAEGVPGEPPSVPLLLPPGK**MPMPEPCELEERFALVLSSMNLPPDKARLLRQYDNEKKWDLICDQERFQVKNPPHTYIQKLQSFLDPSVTRKKFRRRVQESTKVLRELEISLRTNHIGWVREFLNDENKGLDVLVDYLSFAQCSVMFDFEGLESGDDGAFDKLRSWSRSIEDLQPPSALSAPFTNSLARSARQSVLRYSTLPGRRALKNSRLVSQKDDVHVCILCLRAIMNYQYGFNLVMSHPHAVNEIALSLNNKNPRTKALVLELLAAVCLVRGGHEIILAAFDNFKEVCKELHRFEKLMEYFRNEDSNIDFMVACMQFINIVVHSVEDMNFRVHLQYEFTKLGLEEFLQKSRHTESEKLQVQIQAYLDNVFDVGGLLEDAETKNVALEKVEELEEHVSHLTEKLLDLENENMMRVAELEKQLLQREKELESIKETYENTSHQVHTLRRLIKEKEEAFQRRCHLE**PNVRGLESVDSEALARVGPAELSEGMPPSDLDLLAPAPPPEEVLPLPPPPAPPLPPPPPPLPDKCPPAPPLPGAAPSVVLTVGLSAIR**IKKPIKTKFRLPVFNWTALKPNQISGTVFSELDDEKILEDLDLDKFEELFKTKAQGPALDLICSKNKTAQKAASKVTLLEANRAKNLAITLRKAGRSAEEICRAIHTFDLQTLPVDFVECLMRFLPTEAEVKLLRQYERERQPLEELAAEDRFMLLFSKVERLTQRMAGMAFLGNFQDNLQMLTPQLNAIIAASASVKSSQKLKQMLEIILALGNYMNSSKRGAVYGFKLQSLDLLLDTKSTDRKMTLLHFIALTVKEKYPDLANFWHELHFVEKAAAVSLENVLLDVKELGRGMELIRRECSIHDNSVLRNFLSTNEGKLDKLQRDAKTAEEAYNAVVRYFGESPKTTPPSVFFPVFVRFIRSYKEAEQENEARKKQEEVMREKQLAQEA**KKLDAKTPSQRNKWQQQELIAELRRRQAKEHRPVYEGKDGTIEDIITGLHCQPMVVRHQARSAAPPSGPPRAPGPH

Hsa1E: FMNL2_ENSP00000288670 (exclusion form)

Hsa1I: FMNL2_ENSP00000288670f10936A (inclusion form)

Hsa2: FMNL3_ENSP00000335655

Sme1: comp3928_c0_seq1

Hsa1E MGNAGSMD---SQQTDFRAHNVPLKLPMPEPGELEERFAIVLN 0 AMNLPPDKARLLRQYDNEKKWELICDQE 0 RFQVKNPPHTYIQKLKGYLDPAVTRKK 0 FRRRVQES-TQVLRELEISLRTNHIG 2 WVRE Hsa1E

Hsa1I MGNAGSMD---SQQTDFRAHNVPLKLPMPEPGELEERFAIVLN 0 AMNLPPDKARLLRQYDNEKKWELICDQE 0 RFQVKNPPHTYIQKLKGYLDPAVTRKK 0 FRRRVQES-TQVLRELEISLRTNHIG 2 WVRE Hsa1I

Hsa2 MGNLESAEGVPGEPPSVPLLLPPGKMPMPEPCELEERFALVLS 0 SMNLPPDKARLLRQYDNEKKWDLICDQE 0 RFQVKNPPHTYIQKLQSFLDPSVTRKK 0 FRRRVQES-TKVLRELEISLRTNHIG 2 WVRE Hsa2

Sme1 MGSTSSKS---TPQYKFINSGEGRLEMRPTNLQFEKRWDTFIQ * SLDVGPENVRQIDQLDPDQKEQLLLNFE 0 SKNPKYSAFHYVTLIKALQTGKSIRKS * FNKKPDDDPVHVLSATEVSLRTNNVD 2 WVYD Sme1

Hsa1E FLNEENKGLDVLVEYLSFAQYAVT 2 - * FDFESVESTVESSV * DKSKPWSRSIEDLHRGSNLPSPVGNSVSRSGRHSALR 2 YNTLPSRRTLKNSRLVSKKDDVHVCIMCLRAIMNYQY 0 GFNMVMSHPHAV Hsa1E

Hsa1I FLNEENKGLDVLVEYLSFAQYAVT 2 - * FDFESVESTVESSV * DKSKPWSRSIEDLHRGSNLPSPVGNSVSRSGRHSALR 2 YNTLPSRRTLKNSRLVSKKDDVHVCIMCLRAIMNYQY 0 GFNMVMSHPHAV Hsa1I

Hsa2 FLNDENKGLDVLVDYLSFAQCSVM 2 - * FDFEGLESGDDGAF * DKLRSWSRSIEDLQPPSALSAPFTNSLARSARQSVLR 2 YSTLPGRRALKNSRLVSQKDDVHVCILCLRAIMNYQY 0 GFNLVMSHPHAV Hsa2

Sme1 FLN--NNGLEVLLDYMSKTLVLFL * N 2 INYKNIETKKTLNL 2 QSDTKTHLSSSDLFKSKKFNCCASIKR---------R * SSVVTKFQNSKDSVNESVKNSLHQGLKCMRAILNNQR 0 GCEMAFVHSRAI Sme1

Hsa1E NEIALSLNNKNPR 2 TKALVLELLAAVCLVRGGHEIILSAFDNFKEV 0 CGEKQRFEKLMEHFRN------EDNNIDFMV 0 ASMQFINIVVHSVEDMNFRVHLQYEFTKLGLDEYLDK 0 LKHTESDKLQVQIQA Hsa1E

Hsa1I NEIALSLNNKNPR 2 TKALVLELLAAVCLVRGGHEIILSAFDNFKEV 0 CGEKQRFEKLMEHFRN------EDNNIDFMV 0 ASMQFINIVVHSVEDMNFRVHLQYEFTKLGLDEYLDK 0 LKHTESDKLQVQIQA Hsa1I

Hsa2 NEIALSLNNKNPR 2 TKALVLELLAAVCLVRGGHEIILAAFDNFKEV 0 CKELHRFEKLMEYFRN------EDSNIDFMV 0 ACMQFINIVVHSVEDMNFRVHLQYEFTKLGLEEFLQK 0 SRHTESEKLQVQIQA Hsa2

Sme1 DVIALCLLHPNYQ 2 TKTLVLETLAAICLIVNGLQKVLAAFEYFKEI * TGESRRFDTLITYFRDHETFKVDDYNIDFMV 0 SCTQFINIVVHSGEDINLRVYLQQEFTILGLDDFYRK 0 LEERSGDRLKKQVAA Sme1

Hsa1E YLDNVFDVGALLEDAETKNAALERVEELEENISHL 0 SEKLQDTENEAMSKIVELEKQLMQRNKELDVVRE 0 IYKDANTQVHTLRKMVKEKEEAIQRQSTLEKKIHELEKQGTIKIQKKGDGDIAILPVVASGTLSM Hsa1E

Hsa1I YLDNVFDVGALLEDAETKNAALERVEELEENISHL 0 SEKLQDTENEAMSKIVELEKQLMQRNKELDVVRE 0 IYKDANTQVHTLRKMVKEKEEAIQRQSTLEKKIHELEKQGTIKIQKKGDGDIAILPVVASGTLSM Hsa1I

Hsa2 YLDNVFDVGGLLEDAETKNVALEKVEELEEHVSHL 0 TEKLLDLENENMMRVAELEKQLLQREKELESIKE 0 TYENTSHQVHTLRRLIKEKEEAFQRRCHLEPNVRGLES--------------------------V Hsa2

Sme1 YNGNRVDVAILLEDSETKETVLLDYEKLEH----- * --EFYANEEKYQTQIVHIENEKTELLQIIDSLKA 0 QKEEQDKEYRTLREQISEPRNSTDREKELVLEINSLKK--------------------------- Sme1

Hsa1E GSEVVAGNSVGPTMGAASSGPLPPPPPPLPPSSDTPETV 1 QNGPVTPPMPPPPPPPPPPPPPPPPPPPPPLPGPAAETVPAPPLAP * PLPSAPPLPGTSSPTVVFNSGLAA 1 VKIKKPIKTKFRMPVFNWVALK Hsa1E

Hsa1I GSEVVAGNSVGPTMGAASSGPLPPPPPPLPPSSDTPETV 1 QNGPVTPPMPPPPPPPPPPPPPPPPPPPPPLPGPAAETVPAPPLAP * PLPSAPPLPGTSSPTVVFNSGLAA 1 VKIKKPIKTKFRMPVFNWVALK Hsa1I

Hsa2 DSEALAR--VGP---AELSEGMPPSDLDLLAPAPPPEEV * L-------------PLPPPPAPPLPPPPPPLP-------------D 1 KCPPAPPLPG-AAPSVVLTVGLSA 1 IRIKKPIKTKFRLPVFNWTALK Hsa2

Sme1 -----VLTEKNIPFNSSVLTELSPNKSSLPLASISP--- * ---------------PPPPIAPPPPIAPPP---------------S * CAPPPPGQPPGVFKLGTGATNTQT 1 QSIRPPIQTKYKLPVFNWSVLK Sme1

Hsa1E PNQINGTVFNEIDDERILED 0 LNVDEFEEIFKTKAQGP----AIDLSSSKQKIPQKGSNKVTLLEANRAK * NLAITLRKAGKTADEICKAIHV 2 FDLKTLPVDFVECLMRFLPTENEVKVLRLYER-ERKPLEN Hsa1E

Hsa1I PNQINGTVFNEIDDERILED 0 LNVDEFEEIFKTKAQGP----AIDLSSSKQKIPQKGSNKVTLLEANRAK * NLAITLRKAGKTADEICKAIHV 2 FDLKTLPVDFVECLMRFLPTENEVKVLRLYER-ERKPLEN Hsa1I

Hsa2 PNQISGTVFSELDDEKILED 0 LDLDKFEELFKTKAQGP----ALDLICSKNKTAQKAASKVTLLEANRAK * NLAITLRKAGRSAEEICRAIHT 2 FDLQTLPVDFVECLMRFLPTEAEVKLLRQYER-ERQPLEE Hsa2

Sme1 NQQLKGTVFVSMNDEVLYDK * LEFEKFENIFKLNPVGDSIPNGVDSNDSSEKTSKRKPEKKTLLDGSKHR 2 CLGVLLKFLDQEKLHSDILCKS * INQLEVSVEVADRILYQLPTNDDIKTYRRYEYSDKLPINA Sme1

Hsa1E LSDEDRFMMQFSKIERLMQKMTIMAFIGNFAESIQMLTPQ 0 LHAIIAASVSIKSSQKLKKILEI 0 ILALGNYMNSSKRGAVYGFKLQSLDLL 0 LDTKSTDRKQTLLHYISNVVKEKYHQVSLFYNELHYVEKAA Hsa1E

Hsa1I LSDEDRFMMQFSKIERLMQKMTIMAFIGNFAESIQMLTPQ 0 LHAIIAASVSIKSSQKLKKILEI 0 ILALGNYMNSSKRGAVYGFKLQSLDLL 0 LDTKSTDRKQTLLHYISNVVKEKYHQVSLFYNELHYVEKAA Hsa1I

Hsa2 LAAEDRFMLLFSKVERLTQRMAGMAFLGNFQDNLQMLTPQ 0 LNAIIAASASVKSSQKLKQMLEI 0 ILALGNYMNSSKRGAVYGFKLQSLDLL 0 LDTKSTDRKMTLLHFIALTVKEKYPDLANFWHELHFVEKAA Hsa2

Sme1 LTDEDRLLLTLCKVERLQQKLEVVVYIHNFPEAYKSLVEK * VTFVTRASLAVRSSQKLKQILEI * ILAYGNLMNSSRRGIMYGFRLQSLDLL 0 TDTKSLDKSWTLMQFFVDIVESKFQNLSDFSDEMRFVEQAS Sme1

Hsa1E AV 1 SLENVLLDVKELQRGMDLTKREYTMHDHN-TLLKEFILNNEGKLKKLQDDAKIAQD 0 AFDDVVKYFGENPKTTPPSVFFPVFVRFVKAYKQ 0 AEEENELRKKQEQALMEKLLEQEALMEQQDPKS 0 PSH Hsa1E

Hsa1I AV 1 SLENVLLDVKELQRGMDLTKREYTMHDHN-TLLKEFILNNEGKLKKLQDDAKIAQD 0 AFDDVVKYFGENPKTTPPSVFFPVFVRFVKAYKQ 0 AEEENELRKKQEQALMEKLLEQEALMEQQDPKS 0 PSH Hsa1I

Hsa2 AV 1 SLENVLLDVKELGRGMELIRRECSIHDN--SVLRNFLSTNEGKLDKLQRDAKTAEE 0 AYNAVVRYFGESPKTTPPSVFFPVFVRFIRSYKE 0 AEQENEARKKQEEVMREKQLAQEAKK--LDAKT 0 PSQ Hsa2

Sme1 KI 1 PMEAITTDVASLVGGMKQVRDEMNCNTNQPPRLKKFFEDNENLTADLSKSAANAKE * TFLQAAEWFGERQNNPTPDVFFGIFHRFTENFKK 0 SLED--VRKRRAQSIILNIVPTEETPKAKPKDK 0 NLS Sme1

Hsa1E KSKRQQ * QELIAELRRRQVKDNRHVYEGKDGAIEDIITD 1 LRNQPYRRADAVRRSVRRRFDDQNLRSVNGAEITM Hsa1E

Hsa1I KSKRQQ * QELIAELRRRQVKDNRHVYEGKDGAIEDIITA 1 LKKNNITKFPNVHSRVRISSSTPVVEDTQS----- Hsa1I

Hsa2 RNKWQQ * QELIAELRRRQAKEHRPVYEGKDGTIEDIITG 1 LHCQPMVVRHQARSAAPPSGPPRAPGPH------- Hsa2

Sme1 MEELQK 0 KLALEARKKNRRMNNRTRQINADGIMDDILAG 1 LDKTPLQAEIHPKRSNLRYQEEVF----------- Sme1

**ORTHOLOGOUS GROUP #5: *MAP/microtubule affinity-regulating kinase (MARK)***


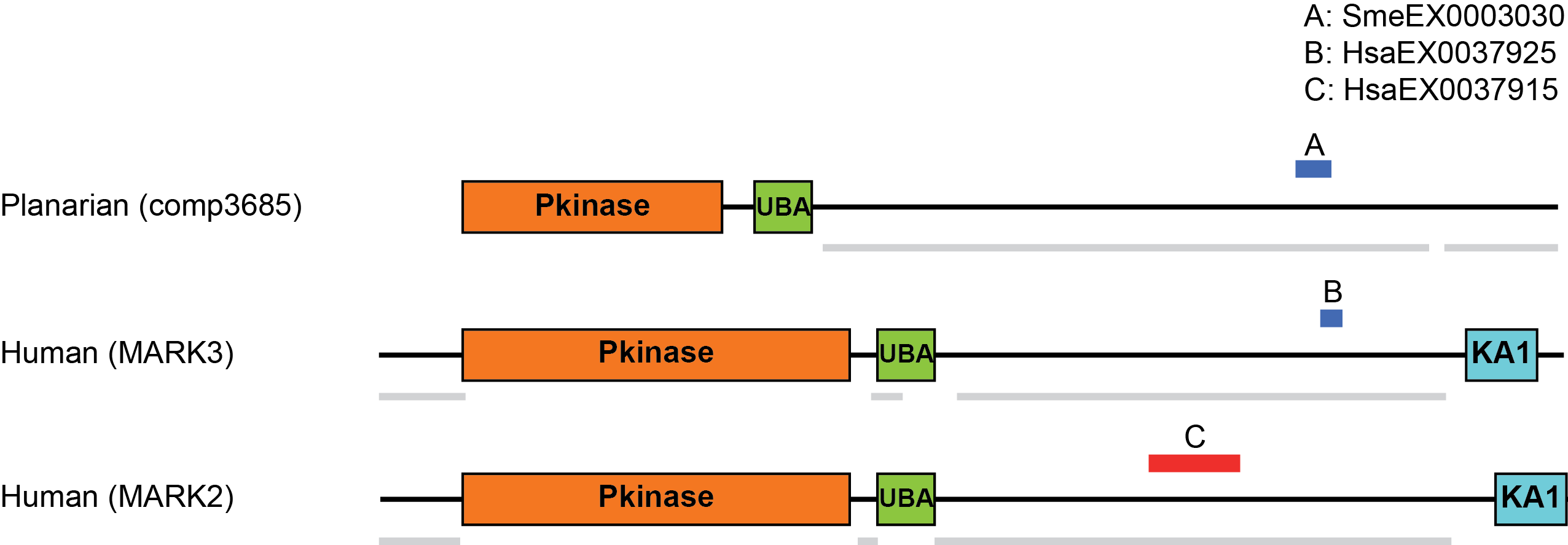


**- AS Exon key:**

SmeEX0003030

HsaEX0037925

HsaEX0037915

**- Protein Domain key:**

**PROTEIN_KINASE_DOM**

**Ubiquitin-associated domain (UBA)**

**- Planarian proteins:**

>comp3685_c0_seq1_2 89.0 163.0 246.2 334.1 450.2 544.2 561.2 642.2

**GEVFDYLVAHGRMKEKEARLKFRQIVSAVQYCHQKKIVHRDLKAENLLLDKEMNIKIADFGFSNEFKPGDKLDTFCGSPPYAAPELFQGKKYDGPEVDVWSLGVILYTLVSGSLPFDGQNLRDLRERVLKGKYRIPFYMSTDCESLLRKMLVLNPQKRYSLEAVMKDRWM**NIGFEENQLTPYIEPPPN**YHDPVRYELMVNMGFTHAEIENSLTEGNFDNITATYFLLEE**RTTNLETDSQGSNASLRQAHVNGSIKPTAPVNQVIASSNTANNVSSNTPTSTSNATSSISSKSQVRPESTSTNHGQSIPSKDNSIFSSNSTKPAGDVKREKKTAVDTNAIFKPTVGNRNDAGRASDRLTINTDKMLNRLQRTTITSANPTTNPSVSARQGDSSTAVAARVNVPMRKINQTTGIVSGGPAKSAGRFGTSTESPSSKFNNAQSTGSRVADLRSHVTPAAVPMSDRTHPKVRTSQPAQNIQHSIVTTNVTSSSPSTNASHNTSVVSEKTDSTPGIAATVPVKSKDNADNKDTDGKPLPSTNQKNITSIPKPPGSFARTQPYQSLRINRPNVPNPPSIAAFPRNSAERRTINNPSNPHADPSVVAGGDLDNQPIGQTIGTTNRGLTSSNTNSGLGFFNKIVKIGNKGSSSKSTTGNQQYSAQNPSLSSAGNAPNFQVNNMGSSGISQFEQTKLSNPTDDGGGSPESYGNTGAIMSDQ

**- Human proteins:**

>MARK3_ENSP00000411397 18.0 82.0 100.0 116.1 138.1 162.0 181.0 260.0 300.0 333.1 371.0 422.1 495.0 529.2 615.2 624.2 639.2

MSTRTPLPTVNERDTENHTSHGDGRQEVTSRTSRSGARCRNSIASCADEQPHIGN**YRLLKTIGKGNFAKVKLARHILTGREVAIKIIDKTQLNPTSLQKLFREVRIMKILNHPNIVKLFEVIETEKTLYLIMEYASGGEVFDYLVAHGRMKEKEARSKFRQIVSAVQYCHQKRIVHRDLKAENLLLDADMNIKIADFGFSNEFTVGGKLDTFCGSPPYAAPELFQGKKYDGPEVDVWSLGVILYTLVSGSLPFDGQNLKELRERVLRGKYRIPFYMSTDCENLLKRFLVLNPIKRGTLEQIMKDRWI**NAGHEEDELKPFVEPELD**ISDQKRIDIMVGMGYSQEEIQESLSKMKYDEITATYLLLG**RKSSELDASDSSSSSNLSLAKVRPSSDLNNSTGQSPHHKVQRSVFSSQKQRRYSDHAGPAIPSVVAYPKRSQTSTADSDLKEDGISSRKSSGSAVGGKGIAPASPMLGNASNPNKADIPERKKSSTVPSSNTASGGMTRRNTYVCSERTTADRHSVIQNGKENSTIPDQRTPVASTHSISSAATPDRIRFPRGTASRSTFHGQPRERRTATYNGPPASPSLSHEATPLSQTRSRGSTNLFSKLTSKLTRRNMSFRFIKRLPTEYERNGRYEGSSRNVSAEQKDENKEAKPRSLRFTWSMKTTSSMDPGDMMREIRKVLDANNCDYEQRERFLLFCVHGDGHAENLVQWEMEVCKLPRLSLNGVRFKRISGTSIAFKNIASKIANELKL

>MARK2_ENSP00000367040 19.0 79.0 97.0 113.1 135.1 159.0 178.0 257.0 297.0 330.1 368.0 412.1 473.0 505.2 559.2 645.2 654.2

MSSARTPLPTLNERDTEQPTLGHLDSKPSSKSNMIRGRNSATSADEQPHIGN**YRLLKTIGKGNFAKVKLARHILTGKEVAVKIIDKTQLNSSSLQKLFREVRIMKVLNHPNIVKLFEVIETEKTLYLVMEYASGGEVFDYLVAHGRMKEKEARAKFRQIVSAVQYCHQKFIVHRDLKAENLLLDADMNIKIADFGFSNEFTFGNKLDTFCGSPPYAAPELFQGKKYDGPEVDVWSLGVILYTLVSGSLPFDGQNLKELRERVLRGKYRIPFYMSTDCENLLKKFLILNPSKRGTLEQIMKDRWM**NVGHEDDELKPYVEPLPD**YKDPRRTELMVSMGYTREEIQDSLVGQRYNEVMATYLLLG**YKSSELEGDTITLKPRPSADLTNSSAPSPSHKVQRSVSANPKQRRFSDQAAGPAIPTSNSYSKKTQSNNAENKRPEEDRESGRKASSTAKVPASPLPGLERKKTTPTPSTNSVLSTSTNRSRNSPLLERASLGQASIQNGKDSLTMPGSRASTASASAAVSAARPRQHQKSMSASVHPNKASGLPPTESNCEVPRPSTAPQRVPVASPSAHNISSSGGAPDRTNFPRGVSSRSTFHAGQLRQVRDQQNLPYGVTPASPSGHSQGRRGASGSIFSKFTSKFVRRNLSFRFARRPHVVGSGGNDKEKEEFREAKPRSLRFTWSMKTTSSMEPNEMMREIRKVLDANSCQSELHEKYMLLCMHGTPGHEDFVQWEMEVCKLPRLSLNGVRFKRISGTSMAFKNIASKIANELKL

Hsa1: MARK2_ENSP00000367040

Hsa2: MARK3_ENSP00000411397

Sme1: comp3685_c0_seq1_2

Hsa1 MSSARTPLPTLNERDTEQP 0 T---LGHLDSKPSSKSNMIRGRNS-ATSADEQPHIGNYRLLKTIGKGNFAKVKLARHILTGKEV 0 AVKIIDKTQLNSSSLQKL 0 FREVRIMKVLNHPNIV 1 KLFEVIETEKT Hsa1

Hsa2 -MSTRTPLPTVNERDTENH 0 TSHGDGRQEVTSRTSRSGARCRNSIASCADEQPHIGNYRLLKTIGKGNFAKVKLARHILTGREV 0 AIKIIDKTQLNPTSLQKL 0 FREVRIMKILNHPNIV 1 KLFEVIETEKT Hsa2

Sme1 ------------------- * ---------------------------------------------------------------- * ------------------ * ---------------- * ----------- Sme1

uuuuuuuuu * uuuuuuuuuuuuuuuuuuuuuuuuuuuuuuuuuuuuuuuuuuuuuuuuuuuuuuuuuuuuuuuu * uuuuuuuuuuuuuuuuuu * uuuuuuuuuuuuuuuu * uuuuuuuuuuu

Hsa1 LYLVMEYASGG 1 EVFDYLVAHGRMKEKEARAKFRQI 0 VSAVQYCHQKFIVHRDLKA 0 ENLLLDADMNIKIADFGFSNEFTFGNKLDTFCGSPPYAAPELFQG * KKYDGPEVDVWSLGVILYTLVSGSLPFDG Hsa1

Hsa2 LYLIMEYASGG 1 EVFDYLVAHGRMKEKEARSKFRQI 0 VSAVQYCHQKRIVHRDLKA 0 ENLLLDADMNIKIADFGFSNEFTVGGKLDTFCGSPPYAAPELFQG * KKYDGPEVDVWSLGVILYTLVSGSLPFDG Hsa2

Sme1 ----------G * EVFDYLVAHGRMKEKEARLKFRQI * VSAVQYCHQKKIVHRDLKA * ENLLLDKEMNIKIADFGFSNEFKPGDKLDTFCGSPPYAAPELFQG 0 KKYDGPEVDVWSLGVILYTLVSGSLPFDG Sme1

uuuuuuuuuuu * uuuuuuuuuuuuuuuuuuuuuuuu * uuuuuuuuuuuuuuuuuuu * uuuuuuuuuuuuuuuuuuuuuuuuuuuuuuuuuuuuuuuuuuuuu * uuuuuuuuuuuuuuuuuuuuuuuuuuuuu

Hsa1 QNLKE 0 LRERVLRGKYRIPFYMSTDCENLLKKFLILNPSKRGTLEQ 0 IMKDRWMNVGHEDDELKPYVEPLPDYKDPRRTE 1 LMVSMGYTREEIQDSLVGQRYNEVMATYLLLGYKSSEL 0 EGD--------- Hsa1

Hsa2 QNLKE 0 LRERVLRGKYRIPFYMSTDCENLLKRFLVLNPIKRGTLEQ 0 IMKDRWINAGHEEDELKPFVEPELDISDQKRID 1 IMVGMGYSQEEIQESLSKMKYDEITATYLLLGRKSSEL 0 DAS--------- Hsa2

Sme1 QNLRD * LRERVLKGKYRIPFYMSTDCESLLRKMLVLNPQKRYSLEA 0 VMKDRWMNIGFEENQLTPYIEPPPNYHDPVRYE * LMVNMGFTHAEIENSLTEGNFDNITATYFLLEERTTNL * ETDSQGSNASLR Sme1

uuuuu * uuuuuuuuuuuuuuuuuuuuuuuuuuuuuuuuuuuuuuuu 0 uuuuuuuuuuuuuuuuuuuuuuuuuuuuuuuuu * uuuuuuuuuuuuuuuuuuuuuuuuuuuuuuuuuuuuuu * uuuuuuuuuuuu

Hsa1 * --------TIT-------LKPRPSADLTNSSAPSPSHKVQRSVSANPKQRRFSDQA 1 AGPAIPT------------SNSYSKKTQSNNA * ENKRPEEDRESGRKASSTAKVPASPLPGLERKKTTPTPSTN 0 Hsa1

Hsa2 * --------DSSSSSNLSLAKVRPSSDLNNSTGQSPHHKVQRSVFSSQKQRRYSDHA 1 G-PAIPS------------VVAYPKRSQTSTA * DS-DLKEDGISSRKSSGSAVGGKGIAP------------AS * Hsa2

Sme1 2 QAHVNGSIKPTAPVNQVIASSNTANNVSSNTPTSTSNATSSISSKSQVRPESTSTN * HGQSIPSKDNSIFSSNSTKPAGDVKREKKTAV 1 DTNAIFKPTVGNRNDAGRASDRLTINTDKMLNRLQRTTITS * Sme1

* uuuuuuuuuuuuuuuuuuuuuuuuuuuuuuuuuuuuuuuuuuuuuuuuuuuuuuuu * uuuuuuuuuuuuuuuuuuuuuuuuuuuuuuuu * uuuuuuuuuuuuuuuuuuuuuuuuuuuuuuuuuuuuuuuuu *

Hsa1 SVLSTSTNRSRNSPLLERASLGQASIQNGKDS 2 LTMPGSRASTASAS * AAVSAARPRQHQKSMSASVHPNKASGLPP * TESNC * EVPR------------PS 2 TAPQRVPVASPSAHNISSSGGAPDRT Hsa1

Hsa2 PMLGNASNPNK------------ADIPERKKS * STVP--------SS 0 NTASGGMTRRNTYVCSERTTADRHSVIQN * GKENS 2 TIP--------------- * --DQRTPVAS--THSISS-AATPDRI Hsa2

Sme1 ANPTTNPSVSARQGDSSTAVAARVNVPMRKIN * QTTGIVSGGPAKSA * GRFGTSTESPSSKFNNAQSTGSRVADLRS 2 HVTPA * AVPMSDRTHPKVRTSQPA * QNIQHSIVTTNVTSSSPSTNASHNTS Sme1

uuuuuuuuuuuuuuuuuuuuuuuuuuuuuuuu * uuuuuuuuuuuuuu * uuuuuuuuuuuuuuuuuuuuuuuuuuuuu * uuuuu * uuuuuuuuuuuuuuuuuu * uuuuuuuuuuuuuuuuuuuuuuuuuu

Hsa1 NFPRGVSSRSTFHAGQLRQVRDQQNLPYGVTPASPSG------HS * QGRRGASGSIFSKFTSK * FVRR 2 NLS-----FRFARR 2 P--------HVVGSG * GND--KEKEEFREAKPRSLRFTWSMKTTSS Hsa1

Hsa2 RFPRGTASRSTFHG----QPRERRTATYNGPPASPSLSHEATPLS * QTRSRGSTNLFSKLTSK * LTRR 2 NMS-----FRFIKR 2 LPTEYERNGRYEGSS 2 RNVSAEQKDENKEAKPRSLRFTWSMKTTSS Hsa2

Sme1 VVSEKTDSTPGIAATVPVKSKDNADNKDTDGKPLPSTNQKNITSI 2 PKPPGSFARTQPYQSLR 2 INRP * NVPNPPSIAAFPRN * SAERRTINNPSNPHA * DPSVVAGGDLDNQPIGQTIGTTNRGLTSSN Sme1

uuuuuuuuuuuuuuuuuuuuuuuuuuuuuuuuuuuuuuuuuuuuu * uuuuuuuuuuuuuuuuu * uuuu * uuuuuuuuuuuuuu * uuuuuuuuuuuuuuu * uuuuuuuuuuuuuuuuuuuuuuuuuuuuuu

Hsa1 MEP-----NEMMREIRKV * LDANSCQSELHEKYMLLCMHGTPGHEDFVQWEMEVCKLPRLSLNGVRFKRISG--TSMAFKNIASKIANELKL Hsa1

Hsa2 MDP-----GDMMREIRKV * LDANNCDYEQRERFLLFCVHGDGHAENLVQWEMEVCKLPRLSLNGVRFKRISG--TSIAFKNIASKIANELKL Hsa2

Sme1 TNSGLGFFNKIVKIGNKG 2 SSSKSTTGNQQYSAQNPSLSSAGNAPNFQVNNMGSSGISQFEQTKLSNPTDDGGGSPESYGNTGAIMSDQ--- Sme1

**ORTHOLOGOUS GROUP #6: *phosphorylase kinase, alpha (PKHA)***


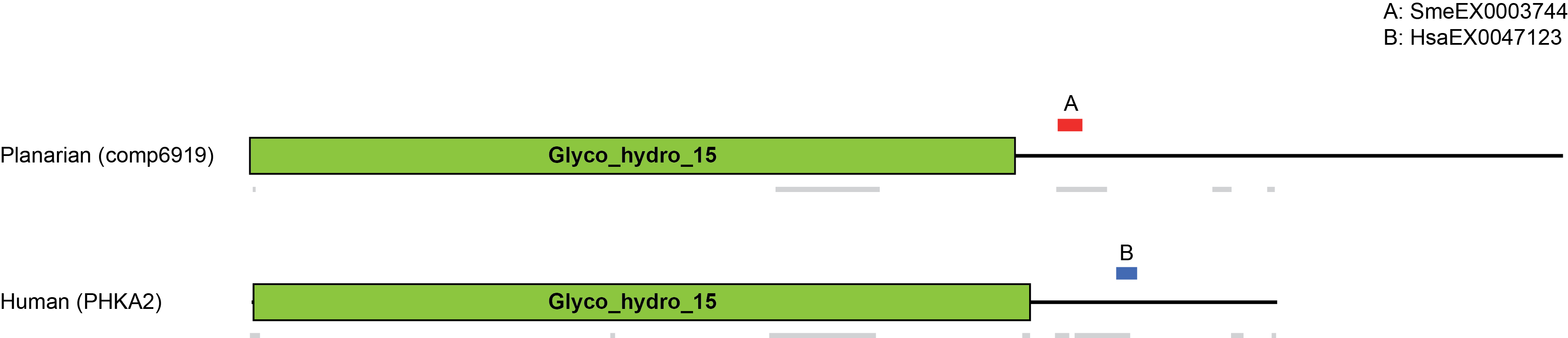


**- AS Exon key:**

SmeEX0003744

HsaEX0047123

**- Protein Domain key:**

**Glyco_hydro_15 super family (split into 2)**

**Planarian C1**

**- Planarian proteins:**

>comp6919_c0_seq1f962A 22.0 75.0 147.1 175.0 202.0 285.2 426.0 485.1 522.0 595.2 621.1 678.1 702.1 794.0 877.0 953.1 971.1 982.0 1061.0

MSKSKLQNFYDYI**KETILKNQDPITGLIPASAESSDSWTRDNCYAVLSVWGLSLAYRKIPDLDEDRSKGYELEKCAIKLMRGILICFLKQADKVEAFKNSQKPSDSLHAKYDSKSCNTIVDDYSWGHLQIDAISLFLLTLAQMTASGINIIWSIDEVSFVQNLVFYIEMAYRIPDYGIWERGDKTNHGLPELNTSSVGMAKAALESLSELDLFGPHGGPQSIIQVFPDECEQCQAVLESMLPRESYSKELDSALISIISYPAFAVNDEELIETTRTKIIEQLLGRYGCKRFMRDGFRTPKEDRNRLHYEPHELRVFEKIECEWPMFFAYLALDACFRDQFKLASEYMDKLEAVMITLPSGFKVVPESYAVRLETMDLEIQNPGSQDREPKGKMPHIWGQSLYLLAKLVTEKIITPGEIDPLGRR**MLMEPKPDLVVQVSVIVENEEMKKRLTKENIPCQTSAELYQQSAIKVFPARTLIYILKGLGKCESLNLQGRVSKEVGVLATSRLYLIGNHMIAFQPQFMDTKDFYISMDFEFIVDVFRTNVAYLRRVWSNPGRPTLVIPLFNWFFKPDGSIEKSILTTLMKLKSGYIYGTRVKLGTLGDFVSTSFVTKLAFLHDKGDDVVRKLLPIESTKKFIRQMSITHDGSPSFKSLTLRRKSVALANAVFADLEGTDVVNDLQRKESILQQWILKSPDEDLYTSRPSNFLKSGPADESRSSTMHLKNLVSGYSDKLRMDNSDPGESNEYLLDRLQATNELGEQGEILFILYQREGLDWRAEIGGEEIF**TVKQMLIELYENAGKSNEWWLVRFISGLLNKRIDILAKSLTDLLIRQKQVTVGLPPEPREIVISTPMAPHELSEVIRKACGEDYAIGMLTQEILVYLAMFARTEPQLLSQMLRIRVGL**IIQVMASELGRALDCSATQASIYLFDLSPSQTKGLLQHILAGREFTLSTS**TVFTNIIREQIHKVPDT**TKRERRKSGLQSTKPVISISGLDIEFSEEKEINKEDSDNNRIGQWIRRRKIDGALNRVPFKFYEKLWKVIERTQAISISNKYLGNNLTKEMTKGEIKFALVVESIINSVPVPEYRQLIVEALCVLSSLVELDAENKIDINYVIPMDKIISNANYLFLLDQVKFNGDSTLCCTKNMTNERRTSLHCRGSNQICIYFYDTSPSGRYGTMTYLLRAFSETIPIPYDKTANSLIDCQVQ

>comp6919_c0_seq1 22.0 75.0 147.1 175.0 202.0 285.2 426.0 485.1 522.0 595.2 621.1 678.1 702.1 794.0 877.0 953.1 964.0 1043.0

MSKSKLQNFYDYI**KETILKNQDPITGLIPASAESSDSWTRDNCYAVLSVWGLSLAYRKIPDLDEDRSKGYELEKCAIKLMRGILICFLKQADKVEAFKNSQKPSDSLHAKYDSKSCNTIVDDYSWGHLQIDAISLFLLTLAQMTASGINIIWSIDEVSFVQNLVFYIEMAYRIPDYGIWERGDKTNHGLPELNTSSVGMAKAALESLSELDLFGPHGGPQSIIQVFPDECEQCQAVLESMLPRESYSKELDSALISIISYPAFAVNDEELIETTRTKIIEQLLGRYGCKRFMRDGFRTPKEDRNRLHYEPHELRVFEKIECEWPMFFAYLALDACFRDQFKLASEYMDKLEAVMITLPSGFKVVPESYAVRLETMDLEIQNPGSQDREPKGKMPHIWGQSLYLLAKLVTEKIITPGEIDPLGRR**MLMEPKPDLVVQVSVIVENEEMKKRLTKENIPCQTSAELYQQSAIKVFPARTLIYILKGLGKCESLNLQGRVSKEVGVLATSRLYLIGNHMIAFQPQFMDTKDFYISMDFEFIVDVFRTNVAYLRRVWSNPGRPTLVIPLFNWFFKPDGSIEKSILTTLMKLKSGYIYGTRVKLGTLGDFVSTSFVTKLAFLHDKGDDVVRKLLPIESTKKFIRQMSITHDGSPSFKSLTLRRKSVALANAVFADLEGTDVVNDLQRKESILQQWILKSPDEDLYTSRPSNFLKSGPADESRSSTMHLKNLVSGYSDKLRMDNSDPGESNEYLLDRLQATNELGEQGEILFILYQREGLDWRAEIGGEEIF**TVKQMLIELYENAGKSNEWWLVRFISGLLNKRIDILAKSLTDLLIRQKQVTVGLPPEPREIVISTPMAPHELSEVIRKACGEDYAIGMLTQEILVYLAMFARTEPQLLSQMLRIRVGL**IIQVMASELGRALDCSATQASIYLFDLSPSQTKGLLQHILAGREFTLSTSKRERRKSGLQSTKPVISISGLDIEFSEEKEINKEDSDNNRIGQWIRRRKIDGALNRVPFKFYEKLWKVIERTQAISISNKYLGNNLTKEMTKGEIKFALVVESIINSVPVPEYRQLIVEALCVLSSLVELDAENKIDINYVIPMDKIISNANYLFLLDQVKFNGDSTLCCTKNMTNERRTSLHCRGSNQICIYFYDTSPSGRYGTMTYLLRAFSETIPIPYDKTANSLIDCQVQ

**- Human proteins:**

>PKHA2_ENSP00000369274 27.0 80.0 96.0 152.1 180.0 207.0 240.0 289.0 307.0 348.0 380.0 416.0 442.1 487.1 524.0 572.1 598.2 655.1 713.1 743.0 787.2 840.0 866.2 893.0 936.1 970.1 1010.0 1020.0 1038.0 1095.0 1113.0 1180.0

MRSRSNS**GVRLDGYARLVQQTILCYQNPVTGLLSASHEQKDAWVRDNIYSILAVWGLGMAYRKNADRDEDKAKAYELEQNVVKLMRGLLQCMMRQVAKVEKFKHTQSTKDSLHAKYNTATCGTVVGDDQWGHLQVDATSLFLLFLAQMTASGLRIIFTLDEVAFIQNLVFYIEAAYKVADYGMWERGDKTNQGIPELNASSVGMAKAALEAIDELDLFGAHGGRKSVIHVLPDEVEHCQSILFSMLPRASTSKEIDAGLLSIISFPAFAVEDVNLVNVTKNEIISKLQGRYGCCRFLRDGYKTPREDPNRLHYDPAELKLFENIECEWPVFWTYFIIDGVFSGDAVQVQEYREALEGILIRGKNGIRLVPELYAVPPNKVDEEYKNPHTVDRVPMGKVPHLWGQSLYILSSLLAEGFLAAGEIDPLNRRFSTSVKPDV**VVQVTVLAENNHIKDLLRKHGVNVQSIADIHPIQVQPGRILSHIYAKLGRNKNMNLSGRPYRHIGVLGTSKLYVIRNQIFTFTPQFTDQHHFYLALDNEMIVEMLRIELAYLCTCWRMTGRPTLTFPISRTMLTNDGSDIHSAVLSTIRKLEDGYFGGARVKLGNLSEFLTTSFYTYLTFLDPDCDEKLFDNASEGTFSPDSDSDLVGYLEDTCNQESQDELDHYINHLLQSTSLRSYLPPLCKNTEDRHVFSAIHSTRDILSVMAKAKGLEVPFVPMTLPTKVLSAHRKSLNLVDSPQPLLEKVPESDFQWPRDDHGDVDCEKLVEQLKDCSNLQDQADILYILYVIKGPSWDTNLSGQHGV**TVQNLLGELYGKAGLNQEWGLIRYISGLLRKKVEVLAEACTDLLSHQKQLTVGLPPEPREKIISAPLPPEELTKLIYEASGQDISIAVLTQEIVVYLAMYVRAQPSLFVEMLRLRIGL**IIQVMATELARSLNCSGEEASESLMNLSPFDMKNLLHHILSGKEFGVERSVRPIHSSTSSPTISIHEVGHTGVTKTERSGINRLRSEMKQMTRRFSADEQFFSVGQAASSSAHSSKSARSSTPSSPTGTSSSDSGGHHIGWGERQGQWLRRRRLDGAINRVPVGFYQRVWKILQKCHGLSIDGYVLPSSTTREMTPHEIKFAVHVESVLNRVPQPEYRQLLVEAIMVLTLLSDTEMTSIGGIIHVDQIVQMASQLFLQDQVSIGAMDTLEKDQATGICHFFYDSAPSGAYGTMTYLTRAVASYLQELLPNSGCQMQ

Sme1E: comp6919_c0_seq1 (exclusion form)

Sme1I: comp6919_c0_seq1f962A (inclusion form)

Hsa1: PKHA2_ENSP00000369274

Sme1E -MSKS----KLQNFYDYIKETILKNQD 0 PITGLIPASAESSDSWTRDNCYAVLSVWGLSLAYRKIPDLDEDRSKGYELEKC 0 AIKLMRGILICFLKQA * DKVEAFKNSQKPSDSLHAKYDSKSCNTIVDDYSWG Sme1E

Sme1I -MSKS----KLQNFYDYIKETILKNQD 0 PITGLIPASAESSDSWTRDNCYAVLSVWGLSLAYRKIPDLDEDRSKGYELEKC 0 AIKLMRGILICFLKQA * DKVEAFKNSQKPSDSLHAKYDSKSCNTIVDDYSWG Sme1I

Hsa1 MRSRSNSGVRLDGYARLVQQTILCYQN 0 PVTGLLSASHEQKDAWVRDNIYSILAVWGLGMAYRKNADRDEDKAKAYELEQN 0 VVKLMRGLLQCMMRQV 0 AKVEKFKHTQSTKDSLHAKYNTATCGTVVGDDQWG Hsa1

Sme1E HLQIDAISLFLLTLAQMTASG 1 INIIWSIDEVSFVQNLVFYIEMAYRIPD 0 YGIWERGDKTNHGLPELNTSSVGMAKA 0 ALESLSELDLFGPHGGPQSIIQVFPDECEQCQA * VLESMLPRESYSKELDSAL Sme1E

Sme1I HLQIDAISLFLLTLAQMTASG 1 INIIWSIDEVSFVQNLVFYIEMAYRIPD 0 YGIWERGDKTNHGLPELNTSSVGMAKA 0 ALESLSELDLFGPHGGPQSIIQVFPDECEQCQA * VLESMLPRESYSKELDSAL Sme1I

Hsa1 HLQVDATSLFLLFLAQMTASG 1 LRIIFTLDEVAFIQNLVFYIEAAYKVAD 0 YGMWERGDKTNQGIPELNASSVGMAKA 0 ALEAIDELDLFGAHGGRKSVIHVLPDEVEHCQS 0 ILFSMLPRASTSKEIDAGL Hsa1

Sme1E ISIISYPAFAVNDEELIETTRTKIIEQLLG * R 2 YGCKRFMRDGFRTPKED * RNRLHYEPHELRVFEKIECEWPMFFAYLALDACFRDQFKLA * SEYMDKLEAVMITLPSGFKVVPESYAVRLETM * DLEI Sme1E

Sme1I ISIISYPAFAVNDEELIETTRTKIIEQLLG * R 2 YGCKRFMRDGFRTPKED * RNRLHYEPHELRVFEKIECEWPMFFAYLALDACFRDQFKLA * SEYMDKLEAVMITLPSGFKVVPESYAVRLETM * DLEI Sme1I

Hsa1 LSIISFPAFAVEDVNLVNVTKNEIISKLQG 0 R * YGCCRFLRDGYKTPRED 0 PNRLHYDPAELKLFENIECEWPVFWTYFIIDGVFSGDAVQV 0 QEYREALEGILIRGKNGIRLVPELYAVPPNKV 0 DEEY Hsa1

Sme1E QNPGSQDREPKGKMPHIWGQSLYLLAKLVTEK * IITPGEIDPLGRRML 0 MEPKPDLVVQV * SVIVENEEMKKRLTKENIPCQTSAELYQQSAIKVFPARTLIYILKGLG 1 KCESLNLQGRVSKEVGVLATSR Sme1E

Sme1I QNPGSQDREPKGKMPHIWGQSLYLLAKLVTEK * IITPGEIDPLGRRML 0 MEPKPDLVVQV * SVIVENEEMKKRLTKENIPCQTSAELYQQSAIKVFPARTLIYILKGLG 1 KCESLNLQGRVSKEVGVLATSR Sme1I

Hsa1 KNPHTVDRVPMGKVPHLWGQSLYILSSLLAEG 0 FLAAGEIDPLNRRFS * TSVKPDVVVQV 1 TVLAENNHIKDLLRKHGVNVQSIADIHP---IQVQPGRILSHIYAKLG 1 RNKNMNLSGRPYRHIGVLGTSK Hsa1

Sme1E LYLIGNHMIAFQPQF 0 MDTKDFYISMDFEFIVDVFRTNVAYLRRVWSNPGRPTLVIPLFNWFFK * PDGS-IEKSILTTLMKLKSGYIYGTR 2 VKLGTLGDFVSTSFVTKLAFLHDKGD 1 D-----VVRKLLP Sme1E

Sme1I LYLIGNHMIAFQPQF 0 MDTKDFYISMDFEFIVDVFRTNVAYLRRVWSNPGRPTLVIPLFNWFFK * PDGS-IEKSILTTLMKLKSGYIYGTR 2 VKLGTLGDFVSTSFVTKLAFLHDKGD 1 D-----VVRKLLP Sme1I

Hsa1 LYVIRNQIFTFTPQF 0 TDQHHFYLALDNEMIVEMLRIELAYLCTCWRMTGRPTLTFPISRTMLT 1 NDGSDIHSAVLSTIRKLEDGYFGGAR 2 VKLGNLSEFLTTSFYTYLTFLDPDCD * EKLFDNASEGTFS Hsa1

Sme1E IESTKKFIRQMSITHDGS * PS------FKSLTLRRKSVALANAVFADLEGTDVVND 1 LQRKESILQQWILKSPDEDLY * T--SR 1 PSNFLKSGPADESRSSTMHLKNLVS * GYSDKLRMDNSDPGESNEY Sme1E

Sme1I IESTKKFIRQMSITHDGS * PS------FKSLTLRRKSVALANAVFADLEGTDVVND 1 LQRKESILQQWILKSPDEDLY * T--SR 1 PSNFLKSGPADESRSSTMHLKNLVS * GYSDKLRMDNSDPGESNEY Sme1I

Hsa1 PDSDSDLVGYLEDTCNQE 1 SQDELDHYINHLLQSTSLRSYLPPLCKNTEDRHVFSA * IHSTRDILSVMAKAKGLEVPF 1 VPMTL * PTKVLSAHRKSLNLVDSPQPLLEKV 0 PESDFQWPRDDHGDVDCEK Hsa1

Sme1E LLDRLQATNELGEQGEILFILYQRE * GLDWRAEIGGEEIFTVKQMLIEL 0 YENAGKSNEWWLVRFISGLLNKRIDILAKS * LTDLLIRQKQVTVGLPPEPREIVIST * PMAPHELSEVIRKACGEDYAIGML Sme1E

Sme1I LLDRLQATNELGEQGEILFILYQRE * GLDWRAEIGGEEIFTVKQMLIEL 0 YENAGKSNEWWLVRFISGLLNKRIDILAKS * LTDLLIRQKQVTVGLPPEPREIVIST * PMAPHELSEVIRKACGEDYAIGML Sme1I

Hsa1 LVEQLKDCSNLQDQADILYILYVIK 2 GPSWDTNLSGQHGVTVQNLLGEL * YGKAGLNQEWGLIRYISGLLRKKVEVLAEA 0 CTDLLSHQKQLTVGLPPEPREKIISA 2 PLPPEELTKLIYEASGQDISIAVL Hsa1

Sme1E TQE 0 ILVYLAMFARTEPQLLSQMLRIRVGLIIQVMASELGRALDCSA * TQASIYLFDLSPSQTKGLLQHILAGREFTLSTS 1 - * --------------------- 1 KRERRKSGLQ--------- * -- Sme1E

Sme1I TQE 0 ILVYLAMFARTEPQLLSQMLRIRVGLIIQVMASELGRALDCSA * TQASIYLFDLSPSQTKGLLQHILAGREFTLSTS 1 T * ----VFTNIIREQIHKVPDTT 1 KRERRKSGLQ--------- * -- Sme1I

Hsa1 TQE 0 IVVYLAMYVRAQPSLFVEMLRLRIGLIIQVMATELARSLNCSG 1 EEASESLMNLSPFDMKNLLHHILSGKEFGVERS * V 1 *RPIHSSTSSPTISIHEVGHTG * VTKTERSGINRLRSEMKQM* 0 TR Hsa1

Sme1E -------- * -------S 0 TKPVISISGL * DIEFSEEKEINKEDSD------NNRIGQWIRRRKIDGALNRVPFKFYEKLWKVIERT * QAISISNKYLGNNLTKEM 0 TKGEIKFALVVESIINSVPVPEYR Sme1E

Sme1I -------- * -------S 0 TKPVISISGL * DIEFSEEKEINKEDSD------NNRIGQWIRRRKIDGALNRVPFKFYEKLWKVIERT * QAISISNKYLGNNLTKEM 0 TKGEIKFALVVESIINSVPVPEYR Sme1I

Hsa1 RFSADEQF 0 FSVGQAAS * SSAHSSKSAR 0 SSTPSSPTGTSSSDSGGHHIGWGERQGQWLRRRRLDGAINRVPVGFYQRVWKILQKC 0 HGLSIDGYVLPSSTTREM 0 TPHEIKFAVHVESVLNRVPQPEYR Hsa1

Sme1E QLIVEALCVLSSLVELDAENKIDINYVIPMDKIISNANYLFLLDQV * KFNGDSTLCCTKNMTNERRTSLHCRGSNQICIYFYDTSPSGRYGTMTYLLRAFSETIPIPYDKTANSLIDCQVQ Sme1E

Sme1I QLIVEALCVLSSLVELDAENKIDINYVIPMDKIISNANYLFLLDQV * KFNGDSTLCCTKNMTNERRTSLHCRGSNQICIYFYDTSPSGRYGTMTYLLRAFSETIPIPYDKTANSLIDCQVQ Sme1I

Hsa1 QLLVEAIMVLTLLSDTEMT---SIGGIIHVDQIVQMASQLFLQDQV 0 SIGAMDTLEKDQ--------------ATGICHFFYDSAPSGAYGTMTYLTRAVASYLQELLPNSG-----CQMQ Hsa1

**ORTHOLOGOUS GROUP #7: *Procollagen-lysine, 2-oxoglutarate 5-dioxygenase (PLOD)***


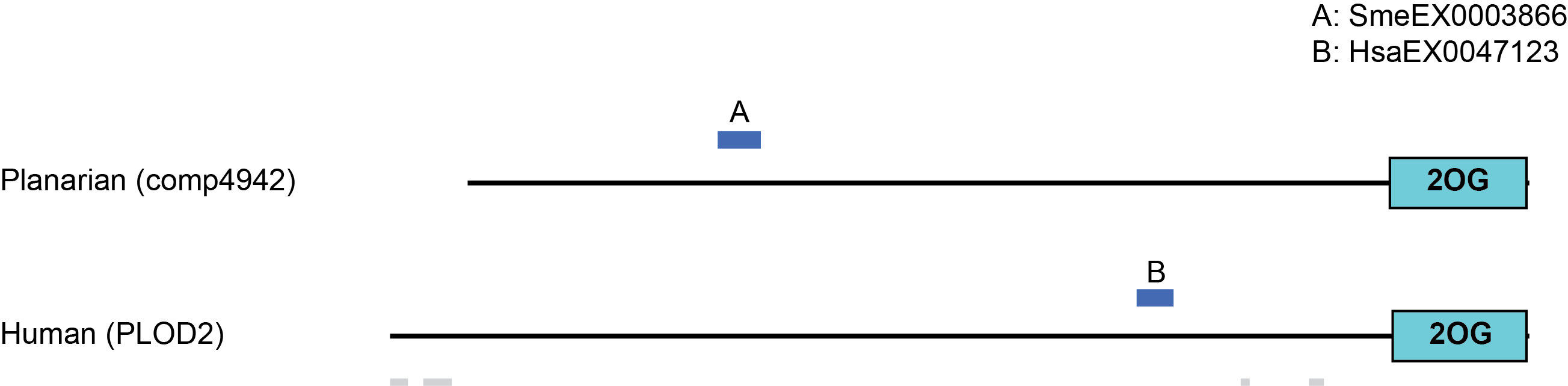


**- AS Exon key:**

SmeEX0003866

HsaEX0047123

**- Protein Domain key:**

**P4Hc Prolyl 4-hydroxylase alpha subunit homologues; Mammalian enzymes catalyse hydroxylation**

**Human C1**

**- Planarian proteins:**

>comp4942_c0_seq1 29.0 74.2 113.0 168.0 189.1 222.0 256.0 300.0 340.2 416.2 464.0 502.0 611.2 654.0

LAVTVATEDNDALARYRNSAEYFNIPYEIFGTGQSWLGGDIKNKPGGGQKVRIVREGLKKYKDREDFIIMFTDSYDVVFQNTSETILNKFKATGAKVLFSAEGFCWPDSSLNDSYPKVGEYEKRFLNSGGFIGYAPYLYKMITLKEIGNEDDDQLYYTEIFLNETLRNEFQMKLDTKSEIFQNLNGVLDEVVLNFQDNLGYLKNVFSGTIPVVIHANGPVKIEFNSMTNYIGHNWNPAEGCQQCKQNKMDLKLKMMVDYPRVTVGIFVNVLTPFIDEFFARIQKLSYPQDKMDVYIYCLADFHLKRCTEFVSKTDNSFASIHLIDSKQFSVEALARNDFIKRSAEINNDFVFFVDSIVHLTKSDAIEHLVSQNRSILAPMVTRRQALWSNFWGALNKNGYYARSEDYLEIVQDVKKGIWNVPFVSSVYMVERNTLKKLAGVYGESHSDSEQFDITFCANVRDKNIFMYVDNQEVFGYQVNAENFTNNHLHNDLWEIFTNPLDWQEKYIHPEYFEIASDDKLMDDVAQPCSDVFWFPLV**SQTFCKQMIEEMENHGQWSDGSNKDNRLEGGYENVPTRDIHMRQVNWEEHWLHVLQTYIYPMQKKLFAGYDDVPSARMNFVVRYRPDEQPTLRPHHDASSYSLNIALNLPGVNYEGGGTKFHRYNCEVKQSKLGWVLMHPGRVTHLHEGLRTTNGTRYI**FVTFVNP

**- Human proteins:**

>PLOD2_ENSP00000282903 37.1 68.0 113.2 168.1 206.0 227.1 260.0 294.0 336.0 376.2 411.2 453.2 501.0 522.0 560.0 582.0 617.0 666.0 708.0

MGGCTVKPQLLLLALVLHPWNPCLGADSEKPSSIPTDKLLVITVATKESDGFHRFMQSAKYFNYTVKVLGQGEEWRGGDGINSIGGGQKVRLMKEVMEHYADQDDLVVMFTECFDVIFAGGPEEVLKKFQKANHKVVFAADGILWPDKRLADKYPVVHIGKRYLNSGGFIGYAPYVNRIVQQWNLQDNDDDQLFYTKVYIDPLKREAINITLDHKCKIFQTLNGAVDEVVLKFENGKARAKNTFYETLPVAINGNGPTKILLNYFGNYVPNSWTQDNGCTLCEFDTVDLSAVDVHPNVSIGVFIEQPTPFLPRFLDILLTLDYPKEALKLFIHNKEVYHEKDIKVFFDKAKHEIKTIKIVGPEENLSQAEARNMGMDFCRQDEKCDYYFSVDADVVLTNPRTLKILIEQNRKIIAPLVTRHGKLWSNFWGALSPDGYYARSEDYVDIVQGNRVGVWNVPYMANVYLIKGKTLRSEMNERNYFVRDKLDPDMALCRNAREMTLQREKDSPTPETFQMLSPPKGVFMYISNRHEFGRLLSTANYNTSHYNNDLWQIFENPVDWKEKYINRDYSKIFTENIVEQPCPDVFWFPIF**SEKACDELVEEMEHYGKWSGGKHHDSRISGGYENVPTDDIHMKQVDLENVWLHFIREFIAPVTLKVFAGYYTKGFALLNFVVKYSPERQRSLRPHHDASTFTINIALNNVGEDFQGGGCKFLRYNCSIESPRKGWSFMHPGRLTHLHEGLPVKNGTRYIAVSFI**DP

Hsa1E: PLOD2_ENSP00000353170 (exclusion form)

Hsa1I: PLOD2_ENSP00000282903 (inclusion form)

Sme1: comp4942_c0_seq1

Hsa1E MGGCTVKPQLLLLALVLHPWNPCLGADSEKPSSIPTD 1 KLLVITVATKESDGFHRFMQSAKYFNYTVKV 0 LGQGEEWRGGDGINSIGGGQKVRLMKEVMEHYADQDDLVVMFTEC 2 FDVIFAGGPEEVLKKFQK Hsa1E

Hsa1I MGGCTVKPQLLLLALVLHPWNPCLGADSEKPSSIPTD 1 KLLVITVATKESDGFHRFMQSAKYFNYTVKV 0 LGQGEEWRGGDGINSIGGGQKVRLMKEVMEHYADQDDLVVMFTEC 2 FDVIFAGGPEEVLKKFQK Hsa1I

Sme1 ------------------------------------- * --LAVTVATEDNDALARYRNSAEYFNIPYEI 0 FGTGQSWLGGDIKNKPGGGQKVRIVREGLKKYKDREDFIIMFTDS 2 YDVVFQNTSETILNKFKA Sme1

uuuuuuuuuuuuuuuuuuuuuuuuuuu * uuuuuuuuuuuuuuuuuuuuuuuuuuuuuuu 0 uuuuuuuuuuuuuuuuuuuuuuuuuuuuuuuuuuuuuuuuuuuuu 2 uuuuuuuuuuuuuuuuuu

Hsa1E ANHKVVFAADGILWPDKRLAD * KYPVV-HIGKRYLNSGG 1 FIGYAPYVNRIVQQWNLQDNDDDQLFYTKVYIDPLKRE 0 AINITLDHKCKIFQTLNGAVD 1 EVVLKFENGKARAKNTFYETLPVAINGNGPT Hsa1E

Hsa1I ANHKVVFAADGILWPDKRLAD * KYPVV-HIGKRYLNSGG 1 FIGYAPYVNRIVQQWNLQDNDDDQLFYTKVYIDPLKRE 0 AINITLDHKCKIFQTLNGAVD 1 EVVLKFENGKARAKNTFYETLPVAINGNGPT Hsa1I

Sme1 TGAKVLFSAEGFCWPDSSLND 0 SYPKVGEYEKRFLNSGG * FIGYAPYLYKMITLKEIGNEDDDQLYYTEIFLNETLRN 0 EFQMKLDTKSEIFQNLNGVLD 1 EVVLNFQDNLGYLKNVFSGTIPVVIHANGPV Sme1

uuuuuuuuuuuuuuuuuuuuu * uuuuuuuuuuuuuuuuu * uuuuuuuuuuuuuuuuuuuuuuuuuuuuuuuuuuuuuu 0 uuuuuuuuuuuuuuuuuuuuu 1 uuuuuuuuuuuuuuuuuuuuuuuuuuuuuuu

Hsa1E KI 0 LLNYFGNYVPNSWTQDNGCTLCEFDTVDLS--AV * DV 0 HPNVSIGVFIEQPTPFLPRFLDILLTLDYPKEALKLFIHNKE 0 VYHEKDIKVFFDKAKHEIKTIKIVGPEENLSQAEARNMGM 2 DFCRQ Hsa1E

Hsa1I KI 0 LLNYFGNYVPNSWTQDNGCTLCEFDTVDLS--AV * DV 0 HPNVSIGVFIEQPTPFLPRFLDILLTLDYPKEALKLFIHNKE 0 VYHEKDIKVFFDKAKHEIKTIKIVGPEENLSQAEARNMGM 2 DFCRQ Hsa1I

Sme1 KI 0 EFNSMTNYIGHNWNPAEGCQQCKQNKMDLKLKMM 0 VD * YPRVTVGIFVNVLTPFIDEFFARIQKLSYPQDKMDVYIYCLA 0 DFHLKRCTEFVSKTDNSFASIHLIDSKQFSVEALARNDFI 2 KRS-A Sme1

uu 0 uuuuuuuuuuuuuuuuuuuuuuuuuuuuuuuuuu * uu * uuuuuuuuuuuuuuuuuuuuuuuuuuuuuuuuuuuuuuuuuu 0 uuuuuuuuuuuuuuuuuuuuuuuuuuuuuuuuuuuuuuuu 2 uuuuu

Hsa1E DEKCDYYFSVDADVVLTNPRTLKILIEQNR 2 KIIAPLVTRHGKLWSNFWGALSPDGYYARSEDYVDIVQGNRV 2 GVWNVPYMANVYLIKGKTLRSEMNERNYFVRDKLDPDMALCRNAREMG 0 ----------- Hsa1E

Hsa1I DEKCDYYFSVDADVVLTNPRTLKILIEQNR 2 KIIAPLVTRHGKLWSNFWGALSPDGYYARSEDYVDIVQGNRV 2 GVWNVPYMANVYLIKGKTLRSEMNERNYFVRDKLDPDMALCRNAREMT 0 LQREKDSPTPE Hsa1I

Sme1 EINNDFVFFVDSIVHLTKSDAIEHLVSQNR * SILAPMVTRRQALWSNFWGALNKNGYYARSEDYLEIVQDVKK 2 GIWNVPFVSSVYMVERNTLKKLAGVYGESHSDSEQFDITFCANVRDKM 0 ----------- Sme1

uuuuuuuuuuuuuuuuuuuuuuuuuuuuuu * uuuuuuuuuuuuuuuuuuuuuuuuuuuuuuuuuuuuuuuuuu 2 uuuuuuuuuuuuuuuuuuuuuuuuuuuuuuuuuuuuuuuuuuuuuuuu * uuuuuuuuuuu

Hsa1E ---------- 0 VFMYISNRHEFGRLLSTANYNTSHYNNDLWQIFENPVD 0 WKEKYINRDYSKIFTEN----IVEQP 0 CPDVFWFPIFSEKACDELVEEMEHYGKWSGGKHHD 0 SRISGGYENVPTDDIHMKQ Hsa1E

Hsa1I TFQMLSPPKG 0 VFMYISNRHEFGRLLSTANYNTSHYNNDLWQIFENPVD 0 WKEKYINRDYSKIFTEN----IVEQP 0 CPDVFWFPIFSEKACDELVEEMEHYGKWSGGKHHD 0 SRISGGYENVPTDDIHMKQ Hsa1I

Sme1 ---------- 0 IFMYVDNQEVFGYQVNAENFTNNHLHNDLWEIFTNPLD 0 WQEKYIHPEYFEIASDDKLMDDVAQP * CSDVFWFPLVSQTFCKQMIEEMENHGQWSDGSNKD * NRLEGGYENVPTRDIHMRQ Sme1

uuuuuuuuuu 0 uuuuuuuuuuuuuuuuuuuuuuuuuuuuuuuuuuuuuu 0 uuuuuuuuuuuuuuuuuuuuuuuuuu * uuuuuuuuuuuuuuuuuuuuuuuuuuuuuuuuuuu * uuuuuuuuuuuuuuuuuuu

Hsa1E VDLENVWLHFIREFIAPVTLKVFAGYYTK * G 0 FALLNFVVKYSPERQRSLRPHHDASTFTINIALNNVGEDFQG 0 GGCKFLRYNCSIESPRKGWSFMHPGRLTHLHEGLPVKNGTRYIAVSFIDP Hsa1E

Hsa1I VDLENVWLHFIREFIAPVTLKVFAGYYTK * G 0 FALLNFVVKYSPERQRSLRPHHDASTFTINIALNNVGEDFQG 0 GGCKFLRYNCSIESPRKGWSFMHPGRLTHLHEGLPVKNGTRYIAVSFIDP Hsa1I

Sme1 VNWEEHWLHVLQTYIYPMQKKLFAGYDDV 2 P * SARMNFVVRYRPDEQPTLRPHHDASSYSLNIALNLPGVNYEG 0 GGTKFHRYNCEVKQSKLGWVLMHPGRVTHLHEGLRTTNGTRYIFVTFVNP Sme1

**ORTHOLOGOUS GROUP #8: *Cytoplasmic linker associated protein (CLASP)***


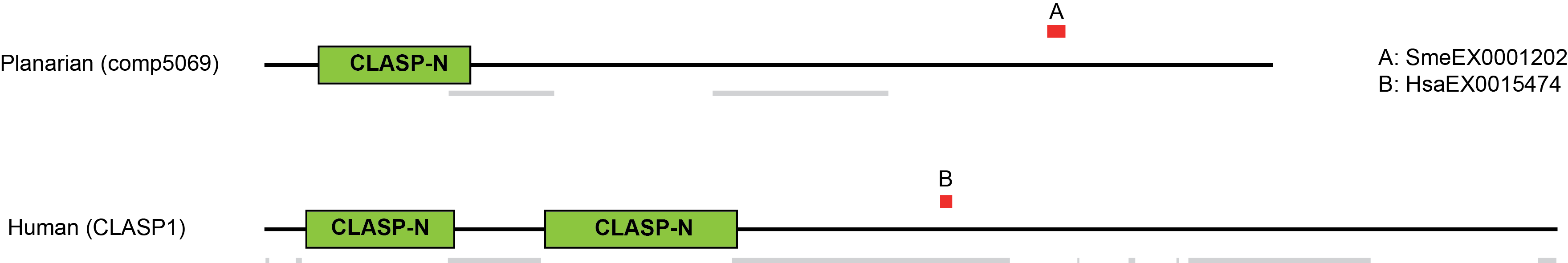


**- AS Exon key:**

SmeEX0001202

HsaEX0015474

**- Protein Domain key:**

**CLASP N terminal**

**- Planarian proteins:**

>comp5069_c0_seq1 730.2 784.0 876.0 892.1 912.1 932.1 975.2 984.0 1097.0 1135.0

MNKKQEFGAVTEEDFEQTFEQTPEIEISSIKELRESLLNINNKLHAD**DWQHRVDNLRILRGIIKANRFPTDDFLQNIKFLDLEIKNCVSDLRSQVVREACITVAFLSKEIKNKLDYFLEQIIESLMNLVSNGVKIMSTSGKVALKYIVYNTNSVRILPFFLTTISTSKSTSLRQFCSELLKLICENWNMKYLEKSSSNIAEAIKKGISDADEQTRAQSREAFCWFNGKFPKLGDKLLQSLDPTKQKALQ**KELLSFKGDEIDSADVFKVPITNAVKTTVKSVTSKPSNFTGRTRVNSAKSENPSANSSTEYLYSKQQSNFGWRTAKPKTATARIDTSDPRGRIQKVSQSQPSTRDPSPSCKSGYITYSNGASDYKYTNNFQKKASPNTYSSLRYKDAFETQVAEGL*KKSRRNSHD*HYQSDDNTSETSSVISDQLPSFNEVVVLLNSKTWNDRKRGLNKAEKYITEASLKPLERKALIDIFNSMFLENQTVVIKRFLTVLYAFVDRYHDHLHDWLYSLIIKLLGLQANERVKSSKMEIQKILNLLIDNYGTIVMFMLCCKFVTDKTNEPTIKIRLCLLEYMKCILQLSQPSLLDTNNPDLGPMLGNILVWTAETKSTDLRKISSNIVIKLFDLNASQFSRLLKLLPNTFQDRATSILNAYLSSQQAVRSTETASDVGDCRVTVKPTNSASKFNRSNSFHTKTTKVYNTKKTASNTTYSSHNSNMSSVSFHKRTPKQKIEPEKSSEFIKINTEALQNLSLNCDNFDKTSVLSNSSNKSGTTPGSHWGSYQKGASSNPFYQTKTSQIPTPRQNLNNYSPANNYSYDNTNMQKFLSSQNSHPELSIDNGGSNVSSSLYPSTSNESNVDNKYNPEYYSSGPIDTSVHSNRDAPQNNTGSISKILKPVIGYQTLRKIAELPPEDMMSEILKELSNHNDRHEERKACLFRLIKMFRENTPTDWDEHFKTLLFLLLETMGDNEIDIENNNAKSEIRAQSLRVLQELAKSQAPRFLEYTQLTILKVLEAHKDPEKSVIKAAEECSRVLTSTMPRKVCLKMLIDVISDGTENQLCLPALKMLIKLIKNSNRESIEADVNNLCQALSIVVNHKESQVRKESVLCMVELHKVIGEGIWSLLKLNNSQARLLEVYIKKGGS

**- Human proteins:**

> CLASP1_ENSP00000386442 66.0 92.1 127.0 157.2 183.0 215.2 238.1 289.1 314.0 345.0 393.2 439.0 462.2 482.2 508.2 564.2 581.1 638.1 673.1 682.1 738.1 773.0 781.0 862.2 884.0 885.0 939.2 966.0 1014.0 1062.0 1097.2 1148.0 1229.1 1293.0 1336.0 1405.0 1444.0

MEPRMESCLAQVLQKDVGKRLQVGQELIDYFSDKQKSADLEHDQTMLDKLVDGLATSWVNSSNYKVVLLGMDI**LSALVTRLQDRFKAQIGTVLPSLIDRLGDAKDSVREQDQTLLLKIMDQAANPQYVWDRMLGGFKHKNFRTREGICLCLIATLNASGAQTLTLSKIVPHICNLLGDPNSQVRDAAINSLVEIYRHVGERVRADLS**KKGLPQSRLNVIFTKFDEVQKSGNMIQSANDKNFDDEDSVDGNRPSSASSTSSKAPPSSRRNVGMGTTRRLGSSTLGSKSSAAKEGAGAVDEEDFIKAFDDVPVVQIYSSRDLEESIN**KIREILSDDKHDWEQRVNALKKIRSLLLAGAAEYDNFFQHLRLLDGAFKLSAKDLRSQVVREACITLGHLSSVLGNKFDHGAEAIMPTIFNLIPNSAKIMATSGVVAVRLIIRHTHIPRLIPVITSNCTSKSVAVRRRCFEFLDLLLQEWQTHSLERHISVLAETIKKGIHDADSEARIEARKCYWGFHSHFSREAEHLYHTLESSYQKALQ**SHLKNSDSIVSLPQSDRSSSSSQESLNRPLSAKRSPTGSTTSRASTVSTKSVSTTGSLQRSRSDIDVNAAASAKSKVSSSSGTTPFSSAAALPPGSYASLGRIRTRRQSSGSATNVASTPDNRGRSRAKVVSQSQRSRSANPAGAGSRSSSPGKLLGSGYGGLTGGSSRGPPVTPSSEKRSKIPRSQGCSRETSPNRIGLDRFGLGQPGRIPGSVNAMRVLSTSTDLEAAVADALLLGDSRSKKKPVRRRYEPYGMYSDDDANSDASSVCSERSYGSRNGGIPHYLRQTEDVAEVLNHCASSNWSERKEGLLGLQNLLKSQRTLSRVELKRLCEIFTRMFADPHSKRVFSMFLETLVDFIIIHKDDLQDWLFVLLTQLLKKMGADLLGSVQAKVQKALDVTRDSFPFDQQFNILMRFIVDQTQTPNLKVKVAILKYIESLARQMDPTDFVNSSETRLAVSRIITWTTEPKSSDVRKAAQIVLISLFELNTPEFTMLLGALPKTFQDGATKLLHNHLKNSSNTSVGSPSNTIGRTPSRHTSSRTSPLTSPTNCSHGGLSPSMLDYDTENLNSEEIYSSLRGVTEAIEKFSFRSQEDLNEPIKRDGKKECDIVSRDGGAASPATEGRGGSEVEGGRTALDNKTSLLNTQPPRAFPGPRARDYNPYPYSDAINTYDKTALKEAVFDDDMEQLRDVPIDHSDLVADLLKELSNHNERVEERKGALLELLKITREDSLGVWEEHFKTILLLLLETLGDKDHSIRALALRVLREILRNQPARFKNYAELTIMKTLEAHKDSHKEVVRAAEEAASTLASSIHPEQCIKVLCPIIQTADYPINLAAIKMQTKVVERIAKESLLQLLVDIIPGLLQGYDNTESSVRKASVFCLVAIYSVIGEDLKPHLAQLTGSKMKLLNLYIKRAQTTNSNSSSSSDVSTHS

Sme1: comp5069_c0_seq1_full

Hsa1: CLASP1_ENSP00000386442

Sme1 ------------------------------------------------------------------ * -------------------------- * ----------------------------------- * ---- Sme1

Hsa1 MEPRMESCLAQVLQKDVGKRLQVGQELIDYFSDKQKSADLEHDQTMLDKLVDGLATSWVNSSNYKV 0 VLLGMDILSALVTRLQDRFKAQIGTV 1 LPSLIDRLGDAKDSVREQDQTLLLKIMDQAANPQY 0 VWDR Hsa1

Sme1 -------------------------- * -------------------------- * -------------------------------- * ----------------------- * --------------------- Sme1

Hsa1 MLGGFKHKNFRTREGICLCLIATLNA 2 SGAQTLTLSKIVPHICNLLGDPNSQV 0 RDAAINSLVEIYRHVGERVRADLSKKGLPQSR 2 LNVIFTKFDEVQKSGNMIQSAND 1 KNFDDEDSVDGNRPSSASSTS Hsa1

Sme1 ----------------------------MN * KKQEFGAVTEEDFEQTFEQTPEIEI * SSIKELRESLLNINNKLHAD--DWQHRVDNL * RILRGIIKANRFPTDDFLQNIKFLDLEIKNCVSDLRSQVVREACI Sme1

Hsa1 SKAPPSSRRNVGMGTTRRLGSSTLGSKSSA 1 AKEGAGAVDEEDFIKAFDDVPVVQI 0 YSSRDLEESINKIREILSDDKHDWEQRVNAL 0 KKIRSLLLAGAAEYDNFFQHLRLLDGAFKLSAKDLRSQVVREACI Hsa1

Sme1 TVA * FLSKEIKNKLDYFLEQIIESLMNLVSNGVKIMSTSGKVALKYIVYN * TNSVRILPFFLTTISTSKSTSLRQ * FCSELLKLICENWNMKYLEK * SSSNIAEAIKKGISDADEQTRAQSRE * AFCWFN Sme1

Hsa1 TLG 2 HLSSVLGNKFDHGAEAIMPTIFNLIPNSAKIMATSGVVAVRLIIRH 0 THIPRLIP-VITSNCTSKSVAVRR 2 RCFEFLDLLLQEWQTHSLER 2 HISVLAETIKKGIHDADSEARIEARK 2 CYWGFH Hsa1

Sme1 GKFPKLGDKLLQSLDPTKQKALQKELLS---------------------- * ----------------- * --FKGDEIDSADVFKVPITNAVKTTVKSVTSKPSNFTGRTRVNSAKSENPSANSSTE * YLYSKQQ Sme1

Hsa1 SHFSREAEHLYHTLESSYQKALQSHLKNSDSIVSLPQSDRSSSSSQESLN 2 RPLSAKRSPTGSTTSRA 1 STVSTKSVSTTGSLQRSRSDIDVNAAASAKSKVSSSSGTTPFSSAAALPPGSYASLG 1 RIRTRRQ Hsa1

Sme1 S-----NFGWRTAKPKTATARIDTSDPR * GRIQKVSQS * QPSTRDPSPSCKSGYITYSNG-------------ASDYKYTNNFQKKASPN----- * ---------------TYSSLRYKDAFETQVAEGLK * Sme1

Hsa1 SSGSATNVASTPDNRGRSRAKVVSQSQR 1 SRSANPAGA 1 GSRSSSPGKLLGSGYGGLTGGSSRGPPVTPSSEKRSKIPRSQGCSRETSPNRIGLD 1 RFGLGQPGRIPGSVNAMRVLSTSTDLEAAVADALL 0 Hsa1

Sme1 KS------ * -----RRNSHDHYQSDDNTSETSSVISD---------------QLPSFNEVVVLLNSKTWNDRKRGLNKAEKYIT-EASLK * PLERKALIDIFNSMFLEN-QTV * V * IKRFLTVLYAFVDRYH Sme1

Hsa1 LGDSRSKK 0 KPVRRRYEPYGMYSDDDANSDASSVCSERSYGSRNGGIPHYLRQTEDVAEVLNHCASSNWSERKEGLLGLQNLLKSQRTLS 2 RVELKRLCEIFTRMFADPHSKR 0 V 0 FSMFLETLVDFIIIHK Hsa1

Sme1 DHLHDWLYSLIIKLLGLQANERVKSSKMEIQKILNLLI * DNYGTIVMFMLCCKFVTDKTNEPTIKI * RLCLLEYMKCILQLSQPSLLDTNNPDLGPMLGNILVWTAETKSTDLRKI * SSNIVIKLFDLNASQFS Sme1

Hsa1 DDLQDWLFVLLTQLLKKMGADLLGSVQAKVQKALDVTR 2 DSFPFDQQFNILMRFIVDQTQTPNLKV 0 KVAILKYIESLARQMDPTDFVNSS-ETRLAVSRIITWTTEPKSSDVRKA 0 AQIVLISLFELNTPEFT Hsa1

Sme1 RLLKLLPNTFQDRATSILNAYLSSQQAVRSTETASDVG * DCRVTVKPTNSASKFNRSNSFHTKTTKVYNTKKTAS * NTTYSSHNSNMSSVSFHKR 2 TPKQKIEPEKSSEFIKINTEALQNLSLNCDNF * DKT Sme1

Hsa1 MLLGALPKTFQDGATKLLHNHLKNS-------SNTSVG 0 SPSNTIGRTPSRHTSSRTSPLTSPTNCSHG-GLSPS 2 MLDYDTENLNSEEIYSSLR * GVTEAIEKFSFRSQEDLNEPIKRDGKKECDIV 0 SRD Hsa1

Sme1 SVLSNSSNKSGTTPGSHWG 0 SYQKGASSNPFYQTKTSQIPTPRQNLNNYSPANNYSYDNTNMQKFLSSQNSHPELSIDNGGSNVSSSLYPSTSNESNVDNKYNPEYYSSGPI 0 DTSVHSNRDA * PQNNTG 1 S Sme1

Hsa1 GGAASPATEGRGGSEVEGG * RTALDNKTSLLNTQPPRAFPGPRARDYNPYPYS-------------------------------------------DAINTYDKTALKEAVF * DDDMEQLRDV 1 PIDHS- * - Hsa1

Sme1 ISKILKPVIGYQTLRKIAE 1 LPPEDMMSEILKELSNHNDR 1 HEERKACLFRLIKMFRENTPTDWDEHFKTLLFLLLETMGDNEI 2 DIENNNAKS 0 EIRAQSLRVLQELAKSQAPRFLEYTQLTILKVLEAHK Sme1

Hsa1 ------------------- * ----DLVADLLKELSNHNER * VEERKGALLELLKITREDSLGVWEEHFKTILLLLLETLGDK-- * -------DH 0 SIRALALRVLREILRNQPARFKNYAELTIMKTLEAHK Hsa1

Sme1 DPEKSV * IKAAEECSRVLTSTMPRKVCLKMLIDVISDGTENQLCLPALKMLIKLIKNSNRESIEADVNNLCQALSIV 0 VNHKESQVRKESVLCMVELHKVIGEGIWS-LLKLNNSQA 0 RLLEVYIKKGGS---- Sme1

Hsa1 DSHKEV 0 VRAAEEAASTLASSIHPEQCIKVLCPIIQT-ADYPINLAAIKMQTKVVERIAKESLLQLLVDIIPGLLQG 0 YDNTESSVRKASVFCLVAIYSVIGEDLKPHLAQLTGSKM 0 KLLNLYIKRAQTTNSN Hsa1

**ORTHOLOGOUS GROUP #9: *Cytoplasmic linker associated protein (MBNL)***


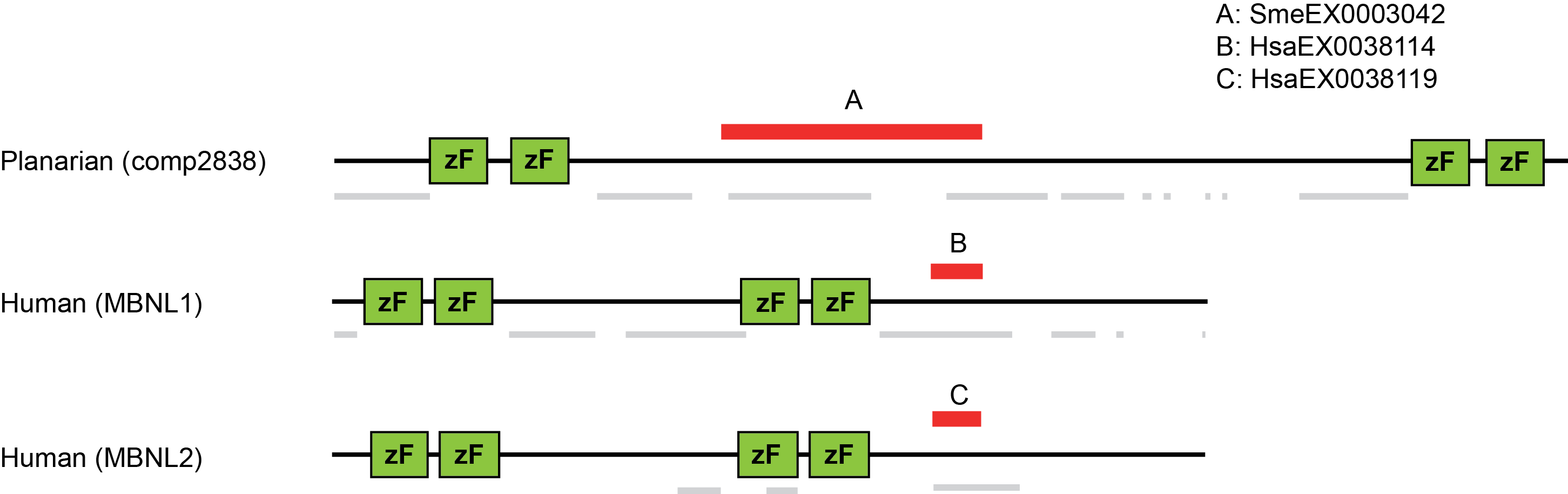


**- AS Exon key:**

SmeEX0003042

HsaEX0038114

HsaEX0038119

**- Protein Domain key:**

**Zn finger**

**- Planarian proteins:**

>comp2838_c0_seq1 2.2 89.0 122.0 176.0 287.0 450.1 478.0 507.1

MIFPNTPANNSLTATHLPQFFNTAAISLPFNYNGTQINYRDSS**WLKLNVCELYLKSECPNVDNCSLAHPPS**TVKIE**NNNVTVCYDYIKKKDCKHGNCKYYHPHD**ALASAILRRGDDSKKNGNALNNMGLNIGIRNNPLQHFNRSHEIINLPNHNLLSSQINSGLMSDSQIKSKLE**VMRQLNTVNAASAWLPGLNVATAQQTLANPATNQANVSLHTLLPGVSAKNSALNQLGLIVSTSPGCSAVKPIVGGYITDTKVSGKRVATEAFGPNSSSGSSNMIHASSDNN**SYIGLSDIKKPKHEANDKTSDKSDTGMESVASVSYVMPQMINSTSQQLQSPSPLAMAGLAGTNINNVYNLLHSTPIQTSAIAPYSQLSPLALNNLMIQSLLTSNSNMANNHHSLDALLGLSAQKIYPMASAYPQVNVAQLNLLNQLYYQQLYQSSLTNQTQMLYPGTINIAEFQQRSFEETSVAFINDKGTI**LETLPVCKDFLQGKCKRNQSCNYVHLLD**SNVEVK**DNKLTVCKNALKGQCHRPVCKFYHIPS**YQLQKLGFST

**- Human proteins:**

>MBNL1 59.0 116.0 184.0 270.0 288.0 339.1 371.0

MAVSVTPIRDTK**WLTLEVCREFQRGTCSRPDTECKFAHPSK**SCQVE**NGRVIACFDSLKGRCSRENCKYLHPPP**HLKTQLEINGRNNLIQQKNMAMLAQQMQLANAMMPGAPLQPVPMFSVAPSLATNASAAAFNPYLGPVSPSLVPAEILPTAPMLVTGNPGVPVPAAAAAAAQKLMR**TDRLEVCREYQRGNCNRGENDCRFAHPAD**STMIDTN**DNTVTVCMDYIKGRCSREKCKYFHPPA**HLQAKIKAAQYQVNQAAAAQAAATAAAMTQSAVKSLKRPLEATFDLGIPQAVLPPLPKRPALEKTNGATAVFNTGIFQYQQALANMQLQQHTAFLPPVPMVHGATPATVSAATTSATSVPFAATATANQIPIISAEHLTSHKYVTQM

>MBNL2

MALNVAPVRDTK**WLTLEVCRQFQRGTCSRSDEECKFAHPPK**SCQVE**NGRVIACFDSLKGRCSRENCKYLHPPT**HLKTQLEINGRNNLIQQKTAAAMLAQQMQFMFPGTPLHPVPTFPVGPAIGTNTAISFAPYLAPVTPGVGLVPTEILPTTPVIVPGSPPVTVPGSTATQKLLR**TDKLEVCREFQRGNCARGETDCRFAHPAD**STMIDTS**DNTVTVCMDYIKGRCMREKCKYFHPPA**HLQAKIKAAQHQANQAAVAAQAAAAAATVMTQSTAKAMKRPLEATVDLAFPPGALHPLPKRQALEKSNGTSAVFNPSVLHYQQALTSAQLQQHAAFIPTGSVLCMTPATSIVPMMHSATSATVSAATTPATSVPFAATATANQIILK

**Intron alignment not needed (different protein regions)**

**ORTHOLOGOUS GROUP #10: *Dynamin (DNM)***


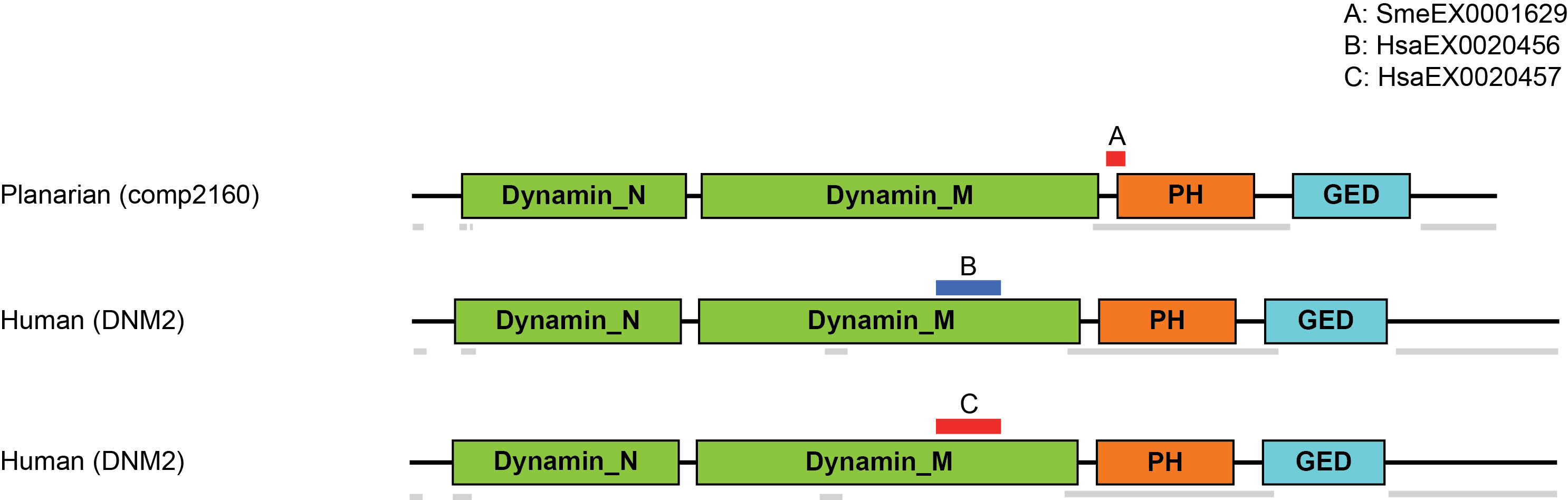


**- AS Exon key:**

SmeEX0001629

HsaEX0020456

HsaEX0020457

(mutually exclusive exons)

**- Protein Domain key:**

**Dynamin-type guanine nucleotide-binding (G) domain**

**PH domain**

**GED domain**

**- Planarian proteins:**

>comp2160_c0_seq1 55.2 80.1 130.1 230.2 332.2 378.0 390.0 412.2 459.0 511.2 528.0 535.0 573.0 652.0 768.0 811.2

MSGNYGMEQLIQVVNRLQDAFVSLNIPL**NLNLPQIAVVGSQSAGKSSVLENFVGRDFLPRGSGIVTRRPLVLQLITATTEYAEFLHCSNKKFTDFEEVRREIEADTERITGKNKGISNTPINLRVYSPNVLNLTLIDLPGMTKVAVGDQPQNIESQIRTMILQYIQEENCLILAVSPANSDLANSDALKISKEVDPGGVRTIGVITKLDLMDEGTDARDILDNKLLPLRRGYVGIVNRSQRDIDGKKDIRTALAAERKFFITHKSYKHLADRMGTAYLQKVLNQQLTNHIRDSLP**QLKNSIENQLASIEREVEACKSIRPEDPNYKTKALMINVQIFETQFIKSIEGAGAEIETKELSGGAIINQIFHERFPYELIKANILYISRDLVQLESDERELRRQISYAIKNNHGIRTGIFIPDMAFEAIVKREIEKLKEPSMKCVDLVVGELIKLIHHCTEKMQNYPRLREEIERLTSQKIRLEEVSAKDQIIELIDMQLAYINTNHADFQRDANGDRKTADGERNKLGNQYSDNVR**KIIREGWLTLQNVTILRGGSKDLYFILTSDSLSWFKDDEKTEKKFDMKLEGLKLKDISGGFMSKKCSFALFNPNQKNVYKDYKQLELVAENQEMLDAWKASFLRAGV**YPTKDETEPSGDEKTDSVNIQGDPQLERQ**VETIRNLVESYMKIVSKSHRDLVPKTIMCIIIDEVKAFLKQEMLANMYSLDMNTLMEESEDEKDRRENLLRSHQALKDALDIINEVSSKTV**SVPVPSAVNDDWRQPDPSVDLSARRPPPPPPNMNKPKPINKGLPPPIMPQRVQSYNPTVP

**- Human proteins:**

>DNM2_ENSP00000468734 54.2 79.1 129.1 197.1 230.1 284.0 331.2 377.0 399.2 446.0 475.0 498.2 516.0 520.0 558.0 594.2 632.0 687.0 764.2 848.2

MGNRGMEELIPLVNKLQDAFSSIGQSC**HLDLPQIAVVGGQSAGKSSVLENFVGRDFLPRGSGIVTRRPLILQLIFSKTEHAEFLHCKSKKFTDFDEVRQEIEAETDRVTGTNKGISPVPINLRVYSPHVLNLTLIDLPGITKVPVGDQPPDIEYQIKDMILQFISRESSLILAVTPANMDLANSDALKLAKEVDPQGLRTIGVITKLDLMDEGTDARDVLENKLLPLRRGYIGVVNRSQKDIEGKKDIRAALAAERKFFLSHPAYRHMADRMGTPHLQKTLNQQLTNHIRESLP**ALRSKLQSQLLSLEKEVEEYKNFRPDDPTRKTKALLQMVQQFGVDFEKRIEGSGDQVDTLELSGGARINRIFHERFPFELVKMEFDEKDLRREISYAIKNIHGVRTGLFTPDLAFEAIVKKQVVKLKEPCLKCVDLVIQELINTVRQCTSKLSSYPRLREETERIVTTYIREREGRTKDQILLLIDIEQSYINTNHEDFIGFANAQQRSTQLNKKRAIPNQGEI**LVIRRGWLTINNISLMKGGSKEYWFVLTAESLSWYKDEEEKEKKYMLPLDNLKIRDVEKGFMSNKHVFAIFNTEQRNVYKDLRQIELACDSQEDVDSWKASFLRAGV**YPEKDQAENEDGAQENTFSMDPQLERQ**VETIRNLVDSYVAIINKSIRDLMPKTIMHLMINNTKAFIHHELLAYLYSSADQSSLMEESADQAQRRDDMLRMYHALKEALNIIGDISTSTV**STPVPPPVDDTWLQSASSHSPTPQRRPVSSIHPPGRPPAVRGPTPGPPLIPVPVGAAASFSAPPIPSRPGPQSVFANSDLFPAPPQIPSRPVRIPPGIPPGVPRRPPAAPSRPTIIRPAEPSLLD

Hsa1A: DNM2_ENSP00000468734

Hsa1B: DNM2_ENSP00000313164

Sme1: comp2160_c0_seq1

Hsa1A -MGNRGMEELIPLVNKLQDAFSSIGQSCH**LDLPQIAVVGGQSAGKSSVLENFVGR 2 DFLPRGSGIVTRRPLILQLIFSKTE 1 HAEFLHCKSKKFTDFDEVRQEIEAETDRVTGTNKGISPVPINLRVYSPHV 1 L** Hsa1A

Hsa1B -MGNRGMEELIPLVNKLQDAFSSIGQSCH**LDLPQIAVVGGQSAGKSSVLENFVGR 2 DFLPRGSGIVTRRPLILQLIFSKTE 1 HAEFLHCKSKKFTDFDEVRQEIEAETDRVTGTNKGISPVPINLRVYSPHV 1 L** Hsa1B

Sme1 MSGNYGMEQLIQVVNRLQDAFVSLNIPLN**LNLPQIAVVGSQSAGKSSVLENFVGR 2 DFLPRGSGIVTRRPLVLQLITATTE 1 YAEFLHCSNKKFTDFEEVRREIEADTERITGKNKGISNTPINLRVYSPNV 1 L** Sme1

Hsa1A **NLTLIDLPGITKVPVGDQPPDIEYQIKDMILQFISRESSLILAVTPANMDLANSDALKLAKEVDPQG 1 LRTIGVITKLDLMDEGTDARDVLENKLLPLRR * G 1 YIGVVNRSQKDIEGKKDIRAALAAERKFFLS** Hsa1A

Hsa1B **NLTLIDLPGITKVPVGDQPPDIEYQIKDMILQFISRESSLILAVTPANMDLANSDALKLAKEVDPQG 1 LRTIGVITKLDLMDEGTDARDVLENKLLPLRR * G 1 YIGVVNRSQKDIEGKKDIRAALAAERKFFLS** Hsa1B

Sme1 **NLTLIDLPGMTKVAVGDQPQNIESQIRTMILQYIQEENCLILAVSPANSDLANSDALKISKEVDPGG * VRTIGVITKLDLMDEGTDARDILDNKLLPLRR 2 G * YVGIVNRSQRDIDGKKDIRTALAAERKFFIT** Sme1

Hsa1A **HPAYRHMADRMGTPHLQKTLNQQ 0 LTNHIRESLP**ALRSKLQSQLLSLEKEVEEYKNFRPDDPTRKTKALLQ 2 MVQQFGVDFEKRIEGSGDQVDTLELSGGARINRIFHERFPFELVK- * -----------M 0 Hsa1A

Hsa1B **HPAYRHMADRMGTPHLQKTLNQQ 0 LTNHIRESLP**ALRSKLQSQLLSLEKEVEEYKNFRPDDPTRKTKALLQ 2 MVQQFGVDFEKRIEGSGDQVDTLELSGGARINRIFHERFPFELVK- * -----------M 0 Hsa1B

Sme1 **HKSYKHLADRMGTAYLQKVLNQQ * LTNHIRDSLP**QLKNSIENQLASIEREVEACKSIRPEDPNYKTKALMI 2 NVQIFETQFIKSIEGAGAEIETKELSGGAIINQIFHERFPYELIKA 0 NILYISRDLVQL 0 Sme1

Hsa1A EFDEKDLRREISYAIKNIHGVR 2 TGLFTPDLAFEAIVKKQVVKLKEPCLKCVDLVIQELINTVRQCTSKL 0 SSYPRLREETERIVTTYIREREGRTKDQI 0 LLLIDIEQSYINTNHEDFIGFAN 2 AQQRST- Hsa1A

Hsa1B EFDEKDLRREISYAIKNIHGVR 2 TGLFTPDMAFEAIVKKQIVKLKEPSLKCVDLVVSELATVIKKCAEKL 0 SSYPRLREETERIVTTYIREREGRTKDQI 0 LLLIDIEQSYINTNHEDFIGFAN 2 AQQRST- Hsa1B

Sme1 ESDERELRRQISYAIKNNHGIR 2 TGIFIPDMAFEAIVKREIEKLKEPSMKCVDLVVGELIKLIHHCTEKM 0 QNYPRLREEIERLTSQKIRLEEVSAKDQI * IELIDMQLAYINTNHADFQRDAN 2 GDRKTAD Sme1

Hsa1A -QLNKKRAIP * NQG 0 EILV 0 **IRRGWLTINNISLMKGGSKEYWFVLTAESLSWYKDEEE 0 KEKKYMLPLDNLKIRDVEKGFMSNKHVFAIFNTEQR 2 NVYKDLRQIELACDSQEDVDSWKASFLRAG**VYPE Hsa1A

Hsa1B -QLNKKRAIP * NQP 0 ETLV 0 **IRRGWLTINNISLMKGGSKEYWFVLTAESLSWYKDEEE 0 KEKKYMLPLDNLKIRDVEKGFMSNKHVFAIFNTEQR 2 NVYKDLRQIELACDSQEDVDSWKASFLRAG**VYPE Hsa1B

Sme1 GERNKLGNQY 0 SDN * VRKI 0 **IREGWLTLQNVTILRGGSKDLYFILTSDSLSWFKDDEK 0 TEKKFDMKLEGLKLKDISGGFMSKKCSFALFNPNQK * NVYKDYKQLELVAENQEMLDAWKASFLRAG**VYPT Sme1

Hsa1A KDQA 0 ENEDG * AQENTFSM--DPQLERQVETIRNLVDSYVAIINKSIRDLMPKTIMHLMINNT 0 KAFIHHELLAYLYSSADQSSLMEESADQAQRRDDMLRMYHALKEALNIIGDISTSTVSTPVPPPV * DD Hsa1A

Hsa1B KDQA 0 ENEDG * AQENTFSM--DPQLERQVETIRNLVDSYVAIINKSIRDLMPKTIMHLMINNT 0 KAFIHHELLAYLYSSADQSSLMEESADQAQRRDDMLRMYHALKEALNIIGDISTSTVSTPVPPPV * DD Hsa1B

Sme1 KDET * EPSGD 0 EKTDSVNIQGDPQLERQVETIRNLVESYMKIVSKSHRDLVPKTIMCIIIDEV * KAFLKQEMLANMYS-LDMNTLMEESEDEKDRRENLLRSHQALKDALDIINEVSSKTVSVPVPSAV 0 ND Sme1

Hsa1A TWLQSASSHS 2 PTPQRRPVSSIHPPGRPPAVRGPTPGPPLIPVPVGAAASFSAPPIPSRPGPQSVFANSDLFPAPPQIPSRPVRIPPGIPPGVP- * R 2 RPPAAPSR * PTIIRPAEPSLLD Hsa1A

Hsa1B TWLQSASSHS 2 PTPQRRPVSSIHPPGRPPAVRGPTPGPPLIPVPVGAAASFSAPPIPSRPGPQSVFANSDLFPAPPQIPSRPVRIPPGIPPGVPS 2 R * RPPAAPSR * PTIIRPAEPSLLD Hsa1B

Sme1 DWRQPDPSVD * LSARR-------PPPPPPNMNKPKP------------------------------------------------INKGLP----- * - * -PPIMPQR 2 VQSYNPTVP---- Sme1

**ORTHOLOGOUS GROUP #11: *PTPRF interacting protein, binding protein (PPFIBP)***


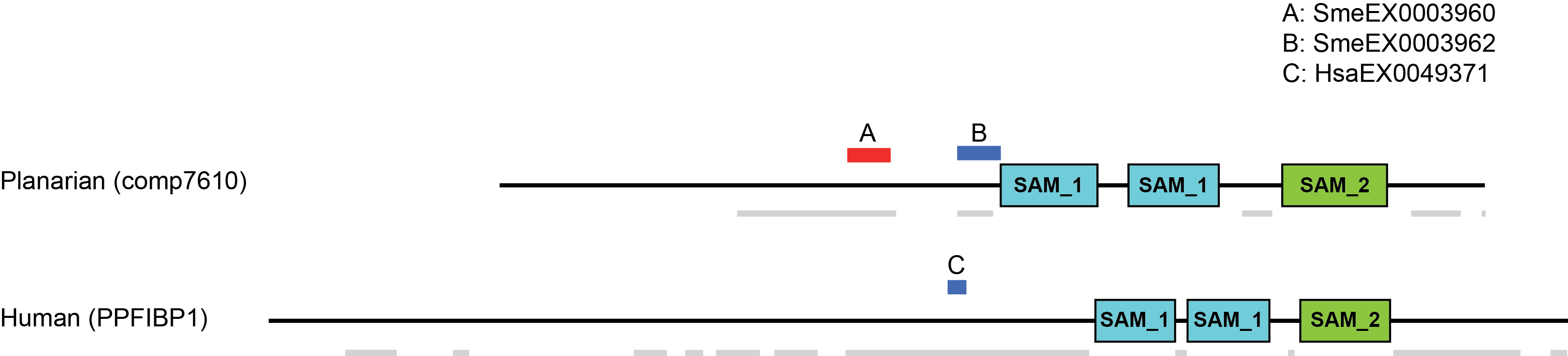


**- AS Exon key:**

SmeEX0003960

SmeEX0003962

HsaEX0049371

**- Protein Domain key:**

**SAM_1 domain**

**SAM_2 domain**

**- Planarian proteins:**

>comp7610_c0_seq1 ... 313.2 329.2 384.2 408.2 455.0 532.0 632.0 695.0

*MSVNYNGYNNNSASKLLESVLEDMNNIIKANDHSGNSENICPLTTKYVDYTDFSNGNNNCSEISHDQKFELMVSELQNCLQEAVDVRSMMRNLSSDKKEFLKALLKTDLENNINISHKVDLMDEISKLKLDIAYLQRDKQGLLNQINDLNSKVQLLNDENVRLRKEMNLMKVDEMDYCFPPNELGDNCSIGESHQSKDSALSGALRNESPLATSTPEKPKSATKVPVVPPHRIQINQRQLPHDFLNKISPPGHIRESTGKSNSWSELRTNQVIHDDQVPMMTPPSQRKSSMNFITNRI*SSDSKKKTKLFSNKSLGLLPCEYDANFDLSICSQPVSSNINALALLAVTDKLAAENSSSQFNRNNPLRMTAGPRLLAGGNKLKPTEVAVQRVLSLSNPNLQSITLLPSKSD**FKLWSKDHVCAFLHQIGLGAYANNCRKWLTNGLQFIEASKKEIEKELNITNKLHLKKLLIHIRQL**TSVHVTAATYFCLTRPKLKIAANFTTRL**WLDDIGLLHYMSLFASNCIDGYMLDQITLEDLQHLKLTNELHVLSLKRGIQLLR**EIDYDLSLLIRRPAENTFNTQSETGSNTNLEVTCNVHPGEV**CRWTQHQVMEWLRRIELPEYASNIRGSGIHGALIILEDRFNANSLASVLNIPQNRSLLRRHLQTKFQEL**IGTKIVAQKKRALENSDFSPVTITSKMKYHRKKSFFNVIKRKTSNETSEELLVPYTYKIEDLDLKLNQETSSVDNNNAESSSTSNLPQTNL

**- Human proteins:**

>PPFIBP1_ENSP00000228425 22.1 91.0 120.0 158.0 202.0 233.0 271.1 282.1 303.0 331.1 383.0 421.2 440.2 461.0 471.0 528.1 539.1 589.2 632.2 683.0 720.1 750.0 791.0 833.0 896.0 956.0 978.0

MMSDASDMLAAALEQMDGIIAGSKALEYSNGIFDCQSPTSPFMGSLRALHLVEDLRGLLEMMETDEKEGLRCQIPDSTAETLVEWLQSQMTNGHLPGNGDVYQERLARLENDKESLVLQVSVLTDQVEAQGEKIRDLEFCLEEHREKVNATEEMLQQELLSRTSLETQKLDLMAEISNLKLKLTAVEKDRLDYEDKFRDTEGLIQEINDLRLKVSEMDSERLQYEKKLKSTKDELASLKEQLEEKESEVKRLQEKLVCKMKGEGVEIVDRDENFKKKLKEKNIEVQKMKKAVESLMAANEEKDRKIEDLRQCLNRYKKMQDTVVLAQGKKGKDGEYEELLNSSSISSLLDAQGFSDLEKSPSPTPVMGSPSCDPFNTSVPEEFHTTILQVSIPSLLPATVSMETSEKSKLTPKPETSFEENDGNIILGATVDTQLCDKLLTSSLQKSSSLGNLKKETSDGEKETIQKTSEDRAPAESRPFGTLPPRPPGQDTSMDDNPFGTRKVRSSFGRGFFKIKSNKRTASAPNLDRKRSASAPTLAETEKETAEHLDLAGASSRPKDSQRNSPFQIPPPSPDSKKKSRGIMKLFGKLRRSQSTTFNPDDMSEPEFKRGGTRATAGPRLGWSRDLGQSNSDLDMP**FAKWTKEQVCNWLMEQGLGSYLNSGKHWIASGQTLLQASQQDLEKELGIKHSLHRKKLQLALQALGSE**EETNHGK**LDFNWVTRWLDDIGLPQYKTQFDEGRVDGRMLHYMTVDDLLSLKVVSVLHHLSIKRAIQVLRIN**NFEPNCLRRRPSDENTIAPSE**VQKWTNHRVMEWLRSVDLAEYAPNLRGSGVHGGLMVLEPRFNVETMAQLLNIPPNKTLLRRHLATHFNLL**IGAEAQHQKRDAMELPDYVLLTATAKVKPKKLAFSNFGNLRKKKQEDGEEYVCPMELGQASGSASKKGFKPGLDMRLYEEDDLDRLEQMEDSEGTVRQIGAFSEGINNLTHMLKEDDMFKDFAARSPSASITDEDSNV

Sme1: comp7610f_c0_seq1

Hsa1: PPFIBP1_ENSP00000228425

Sme1 ---------------------- * -MSVNYNG--YNNNSASKLLES------VLEDMNNIIKANDHSGNSENICPLTTKYVD------YTDFS * NGNNNCSEISHDQKFELMVSELQNCLQEA * VDVRSMMRNLS Sme1

Hsa1 MMSDASDMLAAALEQMDGIIAG 1 SKALEYSNGIFDCQSPTSPFMGSLRALHLVEDLRGLLEMMETDEKEGLRCQIPDSTAETLVEWLQSQMT 0 NGHLPGNGDVYQERLARLENDKESLVLQV 0 SVLTDQVEAQG Hsa1

Sme1 S-------------DKKEFLKALLKTD * LENNINIS-HKVDLMDEISKLKLDIAYLQ----------RDKQG * LLNQINDLNSKVQLLNDENVRLRKEMN---- * ----------------------------- Sme1

Hsa1 EKIRDLEFCLEEHREKVNATEEMLQQE 0 LLSRTSLETQKLDLMAEISNLKLKLTAVEKDRLDYEDKFRDTEG 0 LIQEINDLRLKVSEMDSERLQYEKKLKSTKD 0 ELASLKEQLEEKESEVKRLQEKLVCKMKG Hsa1

Sme1 --------- * ----------- * --------------------L * MKVDEMDYCFP-PNELGDNCSIGESHQS * KDSALSGALRNESP----LATSTPEKPKSATKVPVVPPHRIQINQRQLPHDF * LNKI Sme1

Hsa1 EGVEIVDRD 1 ENFKKKLKEKN 1 IEVQKMKKAVESLMAANEEKD 0 RKIEDLRQCLNRYKKMQDTVVLAQGKKG 1 KDGEYEELLNSSSISSLLDAQGFSDLEKSPSPTPVMGSPSCDPFNTSVPEEF 0 HTTI Hsa1

Sme1 SPP--------------GHIRESTGKSNSWSELR * TNQVIHDDQVP------MM * TPPSQRKSSMNFITNRISSDS * KKKTKLFS-- * -----NKS 2 LGLLP---------------------------- Sme1

Hsa1 LQVSIPSLLPATVSMETSEKSKLTPKPETSFEEN 2 DGNIILGATVDTQLCDKLL 2 TSSLQKSSSLGNLKKETSDGE 0 KETIQKTSED 0 RAPAESRP * FGTLPPRPPGQDTSMDDNPFGTRKVRSSFGRGF Hsa1

Sme1 --------CEYDANFD * LSI 2 CSQPVSSN * INALALLAVTDKLAAENSSSQFNRNNPLRMTAGPRLLAGGNK * LKPTE 2 VAVQRVLSLSNPNLQSITLLPSKS--------------------- 2 Sme1

Hsa1 FKIKSNKRTASAPNLD 1 RKR * SASAPTLA 1 ETEKETAEHLDLAGASSRPKDSQRNSPFQIPPPSPDSKKKSRGIMKLFGK 2 LRRSQSTTFNPDDMSEPEFKRGGTRATAGPRLGWSRDLGQSNSDL 2 Hsa1

Sme1 ---**DFKLWSKDHVCAFLHQIGLGAYANNCRKWLTNGLQFIEASKKEIEKE** 0 LNITNKLHLKKLLIHIRQLTSVHVTAATYFCLTRPKLKIAANFTTR * LWLDDIGLLHYMSLFASNCIDGYMLDQITLE 0 DL Sme1

Hsa1 **DLDMFAKWTKEQVCNWLMEQGLGSYLNSGKHWIASGQTLLQASQQDLEKE** 0 LGIKHSLHRKKLQLALQALGSEEETNHG---------KLDFNWVTR 1 -WLDDIGLPQYKTQFDEGRVDGRMLHYMTVD 0 DL Hsa1

Sme1 QHLKLTNELHVLSLKRGIQLLREIDYDLSLLIRRPAENTFNTQSETGSNTNLEVTC * NVHPGEVCRWTQHQVMEWLRRIELPEYASNIRGSGIHGALII 0 LEDRFNANSLASVLNIPQNRSLLRRHLQTKFQELIG Sme1

Hsa1 LSLKVVSVLHHLSIKRAIQVLRINNFEPNCLRRRPSD-----------------EN 0 TIAPSEVQKWTNHRVMEWLRSVDLAEYAPNLRGSGVHGGLMV 0 LEPRFNVETMAQLLNIPPNKTLLRRHLATHFNLLIG Hsa1

Sme1 TKIVAQKKRALENSDFSPVTITSKMKY 0 HRKKSFFNVIKRKTSNETSEELLVPYTY-------------------------------- * ---------------KIEDLDL * KLNQETSSVDNNNAESSSTSNL Sme1

Hsa1 AEAQHQKRDAMELPDYVLLTATAKVKP 0 KKLAFSNFGNLRKKKQEDGEEYVCPMELGQASGSASKKGFKPGLDMRLYEEDDLDRLEQM 0 EDSEGTVRQIGAFSEGINNLTH 0 MLKEDDMFKDFAARSPSASITD Hsa1

Sme1 PQTNL Sme1

Hsa1 EDSNV Hsa1

**ORTHOLOGOUS GROUP #12: *RAB11 family interacting protein, class II (RAB11FIP)***


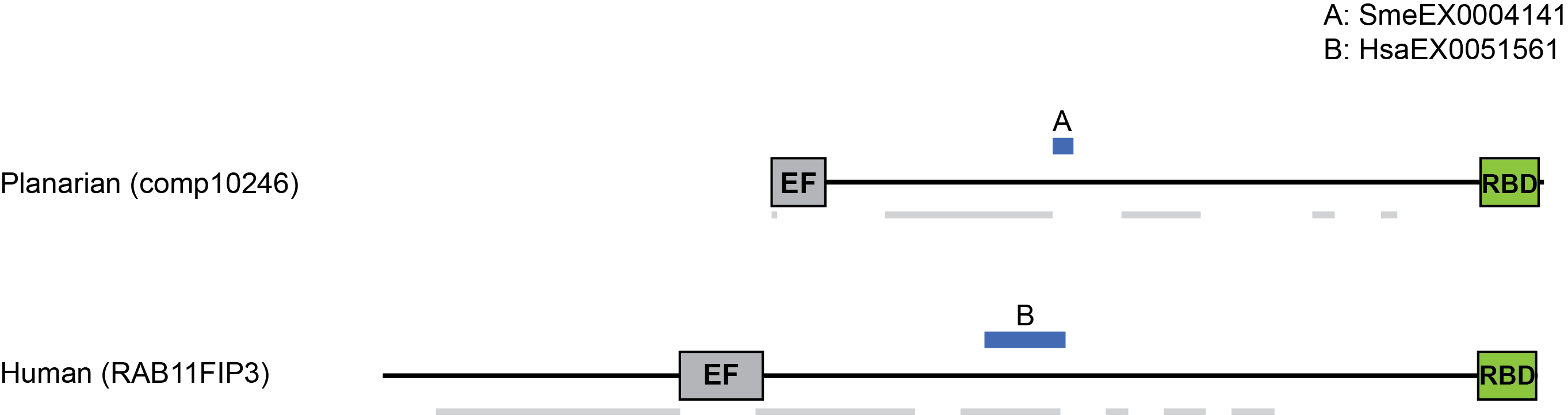


**- AS Exon key:**

SmeEX0004141

HsaEX0051561

**- Protein Domain key:**

**EF-hand, calcium binding motif**

**RBD-FIP domain / RILP (Rab binding domain)**

**FIP domain**

**- Planarian proteins:**

>comp10246_c0_seq1f23A 50.0 81.1 118.0 145.2 189.2 206.2 211.2 260.0 327.0 343.2 371.0 417.0 499.0

**MSYFTEVFHALDHDNKGYILFDDFKNEMLKFLST**GEQSFLTKEKLDQKKTLEQFLNSLTQQNDKIFFTDFIEGIKKLTNSDSKITDNELDNESNCDYHQIFANNDSSPFINDGDNYSTGDSGLDNESGINRENVCNGIGKQNRKRIKAFQNADLSQSIETLKRRFSENESLNNTDYDGQIYKTNQTYHNIPHISLKSSYNCERRKRNYASSDFVTCGSNRINRYSGCEIGDDTKSCHYWTPHSGSVTDLYYHDSMSDIKEKLEKLEQSMENLQTTATQSKTSKFRWMDENQNLKSKIHTLQDIVKDQKKHFEVKLNNEIETSRKYLNKFEKSKLEEIDILNQRLRAKDIELDNLRKEFTTALGECERLKYDKRKSEDKLEDLQQQMGNIEEAMSRLKDFHSRELEILSKERNNLLATVKNSDFEQNSTNVNFPMKNVSSQLLSEISKLRLENQLLKSKLEVCEEEFLLFSIQQGQNILQTIPTDNSWMMEIDGY**TKAEIIDLLVKEKDANFKLREYVSNILTRVIDKDPSLLEIT***

**- Human proteins:**

>RAB11FIP3_ENSP00000398730 239.0 270.1 302.0 372.2 422.2 467.2 479.2 511.0 545.2 592.2 620.0 666.0 718.0 765.0

MASAPPASPPGSEPPGPDPEPGGPDGPGAAQLAPGPAELRLGAPVGGPDPQSPGLDEPAPGAAADGGARWSAGPAPGLEGGPRDPGPSAPPPRSGPRGQLASPDAPGPGPRSEAPLPELDPLFSWTEEPEECGPASCPESAPFRLQGSSSSHRARGEVDVFSPFPAPTAGELALEQGPGSPPQPSDLSQTHPLPSEPVGSQEDGP**RLRAVFDALDGDGDGFVRIEDFIQFATVYGAEQVKDLTKYLDPSGLGVISFEDFYQGIT**AIRNGDPDGQCYGGVASAQDEEPLACPDEFDDFVTYEANEVTDSAYMGSESTYSECETFTDEDTSTLVHPELQPEGDADSAGGSAVPSECLDAMEEPDHGALLLLPGRPHPHGQSVITVIGGEEHFEDYGEGSEAELSPETLCNGQLGCSDPAFLTPSTDPLAAKLHSILTDEAFEFYCSQCHKQINRLEDLSARLSDLEMNSPTKRLSSKKVARYLHQSGALTMEALEDPSPELMEGPEEDIADKVVFLERRVLELEKDTAATGEQHSRLRQENLQLVHRANALEEQLKEQELRACEMVLEETRRQKELLCKMEREKSIEIENLQTRLQQLDEENSELRSCTPCLKANIERLEEEKQKLLDEIESLTLRLSEEQENKRRMGDRLSHERHQFQRDKEATQELIEDLRKQLEHLQLLKLEAEQRRGRSSSMGLQEYHSRARESELEQEVRRLKQDNRNLKEQNEELNGQIITLSIQGAKSLFSTAFSESLAAEISSVS**RDELMEAIQKQEEINFRLQDYIDRIIVAIMETNPSILEVK***

Hsa1I: RAB11FIP3_ENSP00000398730 (inclusion form)

Hsa1E: RAB11FIP3_ENSP00000262305 (exclusion form)

Sme1E: comp10246_c0_seq1 (exclusion form)

Sme1I: comp10246_c0_seq1f23A (inclusion form)

Hsa1I MASAPPASPPGSEPPGPDPEPGGPDGPGAAQLAPGPAELRLGAPVGGPDPQSPGLDEPAPGAAADGGARWSAGPAPGLEGGPRDPGPSAPPPRSGPRGQLASPDAPGPGPRSEAPLPELDPLFSWTEEPEECGPASCPES Hsa1I

Hsa1E MASAPPASPPGSEPPGPDPEPGGPDGPGAAQLAPGPAELRLGAPVGGPDPQSPGLDEPAPGAAADGGARWSAGPAPGLEGGPRDPGPSAPPPRSGPRGQLASPDAPGPGPRSEAPLPELDPLFSWTEEPEECGPASCPES Hsa1E

Sme1E -------------------------------------------------------------------------------------------------------------------------------------------- Sme1E

Sme1I -------------------------------------------------------------------------------------------------------------------------------------------- Sme1I

Hsa1I APFRLQGSSSSHRARGEVDVFSPFPAPTAGELALEQGPGSPPQPSDLSQTHPLPSEPVGSQEDGPR * LRAVFDALDGDGDGFVRIEDFIQFATVYGAEQV 0 KDLTKYLDPSGLGVISFEDFYQGITAIRNGD 1 P Hsa1I

Hsa1E APFRLQGSSSSHRARGEVDVFSPFPAPTAGELALEQGPGSPPQPSDLSQTHPLPSEPVGSQEDGPR * LRAVFDALDGDGDGFVRIEDFIQFATVYGAEQV 0 KDLTKYLDPSGLGVISFEDFYQGITAIRNGD 1 P Hsa1E

Sme1E -------------MSYFTEVFHALDHDNKGYILFDDFKNEMLKFLSTGEQSFLTKEKLDQKK---T 0 LEQFLNSLTQQND-------------------- * -------------KIFFTDFIEGIKKLTNSD 1 - Sme1E

Sme1I -------------MSYFTEVFHALDHDNKGYILFDDFKNEMLKFLSTGEQSFLTKEKLDQKK---T 0 LEQFLNSLTQQND-------------------- * -------------KIFFTDFIEGIKKLTNSD 1 - Sme1I

Hsa1I DGQCYGGVASAQDEEPLACPDEFDDFVTYEA 0 NEVTDSAYMGSESTYSE * CETFTDEDTSTLVHPELQPEGDADSAG * GSAVPSECLDAMEEPDHGALLLLPGR 2 PHPHGQSVITVIGGEEHFEDYGE * G Hsa1I

Hsa1E DGQCYGGVASAQDEEPLACPDEFDDFVTYEA 0 NEVTDSAYMGSESTYSE * CETFTDEDTSTLVHPELQPEGDADSAG * GSAVPSECLDAMEEPDHGALLLLPGR 2 PHPHGQSVITVIGGEEHFEDYGE * G Hsa1E

Sme1E ---------SKITDNELDNESNCDYHQIFAN * NDS--SPFINDGDNYST 0 GDSGLDNESGINRENVCNGIGKQNRKR 2 IKAFQNADLSQSIETLKRRFSENESL * NNTDYDGQIYKTN-----QTYHN 2 I Sme1E

Sme1I ---------SKITDNELDNESNCDYHQIFAN * NDS--SPFINDGDNYST 0 GDSGLDNESGINRENVCNGIGKQNRKR 2 IKAFQNADLSQSIETLKRRFSENESL * NNTDYDGQIYKTN-----QTYHN 2 I Sme1I

Hsa1I SEAELSPETLCNGQLG * -CSDP * AFLTPS 2 TDPLAAKLHSILTDEAFEFYCSQCHKQINRLEDLSARLSDLEMNS 2 PTKRLSSKKVAR 2 YLHQSGALTMEALEDPSPELMEGPEEDIAD * KV 0 VFL Hsa1I

Hsa1E SEAELSPETLCNGQLG * -CSDP * AFLTPS * --------------------------------------------- 2 PTKRLSSKKVAR 2 YLHQSGALTMEALEDPSPELMEGPEEDIAD * KV 0 VFL Hsa1E

Sme1E PHISLKSSYNCERRKS * ----- 2 DFVTCG * S-------------------------------------------N * RINRYSGCEIGD * DTKSCHYWTPHSG-SVTDLYYHDSMSDIKE 0 KL * EKL Sme1E

Sme1I PHISLKSSYNCERRKR 2 NYASS 2 DFVTCG * S-------------------------------------------N * RINRYSGCEIGD * DTKSCHYWTPHSG-SVTDLYYHDSMSDIKE 0 KL * EKL Sme1I

Hsa1I ERRVLELEKDTAATGEQHSRLRQENLQLVHR 2 ANALEEQLKEQELRACEMVLEETRRQKELLC * KMEREKSIEIENLQTR 2 LQQLDEENSELRSCTPCLKANIERLEEE 0 KQKLLDEIESLTLRLSEEQENK Hsa1I

Hsa1E ERRVLELEKDTAATGEQHSRLRQENLQLVHR 2 ANALEEQLKEQELRACEMVLEETRRQKELLC * KMEREKSIEIENLQTR 2 LQQLDEENSELRSCTPCLKANIERLEEE 0 KQKLLDEIESLTLRLSEEQENK Hsa1E

Sme1E EQSMENLQTTATQSKTSKFRWMDENQNLKSK * IHTLQDIVKDQKKHFEVKLNNEIETSRKYLN 0 KFEKSKLEEIDILNQR 2 LRAKDIELDNLRKEFTTALGECERLKYD 0 KRKSEDKLEDLQQQMGNIEEAM Sme1E

Sme1I EQSMENLQTTATQSKTSKFRWMDENQNLKSK * IHTLQDIVKDQKKHFEVKLNNEIETSRKYLN 0 KFEKSKLEEIDILNQR 2 LRAKDIELDNLRKEFTTALGECERLKYD 0 KRKSEDKLEDLQQQMGNIEEAM Sme1I

Hsa1I RRMGDRLSHERHQFQRDKEATQEL 0 IED * LRKQLEHLQLLKLEAEQRRGRSSSMGLQEYHSRARESELEQEVRRLKQD 0 NRNLKEQNEELNGQIITLSIQGAKSLFSTAFSES-LAAEISSVSRDEL 0 MEAI Hsa1I

Hsa1E RRMGDRLSHERHQFQRDKEATQEL 0 IED * LRKQLEHLQLLKLEAEQRRGRSSSMGLQEYHSRARESELEQEVRRLKQD 0 NRNLKEQNEELNGQIITLSIQGAKSLFSTAFSES-LAAEISSVSRDEL 0 MEAI Hsa1E

Sme1E SRLKDFHSRELEILSKER---NNL * LAT 0 VKNS---------DFEQNSTNVN------FPMKNVSSQLLSEISKLRLE * NQLLKSKLEVCEEEFLLFSIQQGQNILQTIPTDNSWMMEIDGYTKAEI 0 IDLL Sme1E

Sme1I SRLKDFHSRELEILSKER---NNL * LAT 0 VKNS---------DFEQNSTNVN------FPMKNVSSQLLSEISKLRLE * NQLLKSKLEVCEEEFLLFSIQQGQNILQTIPTDNSWMMEIDGYTKAEI 0 IDLL Sme1I

Hsa1I QKQEEINFRLQDYIDRIIVAIMETNPSILEVK Hsa1I

Hsa1E QKQEEINFRLQDYIDRIIVAIMETNPSILEVK Hsa1I

Sme1E VKEKDANFKLREYVSNILTRVIDKDPSLLEIT Sme1I

Sme1I VKEKDANFKLREYVSNILTRVIDKDPSLLEIT Sme1I

**ORTHOLOGOUS GROUP #13: *SET binding factor (SFB)***


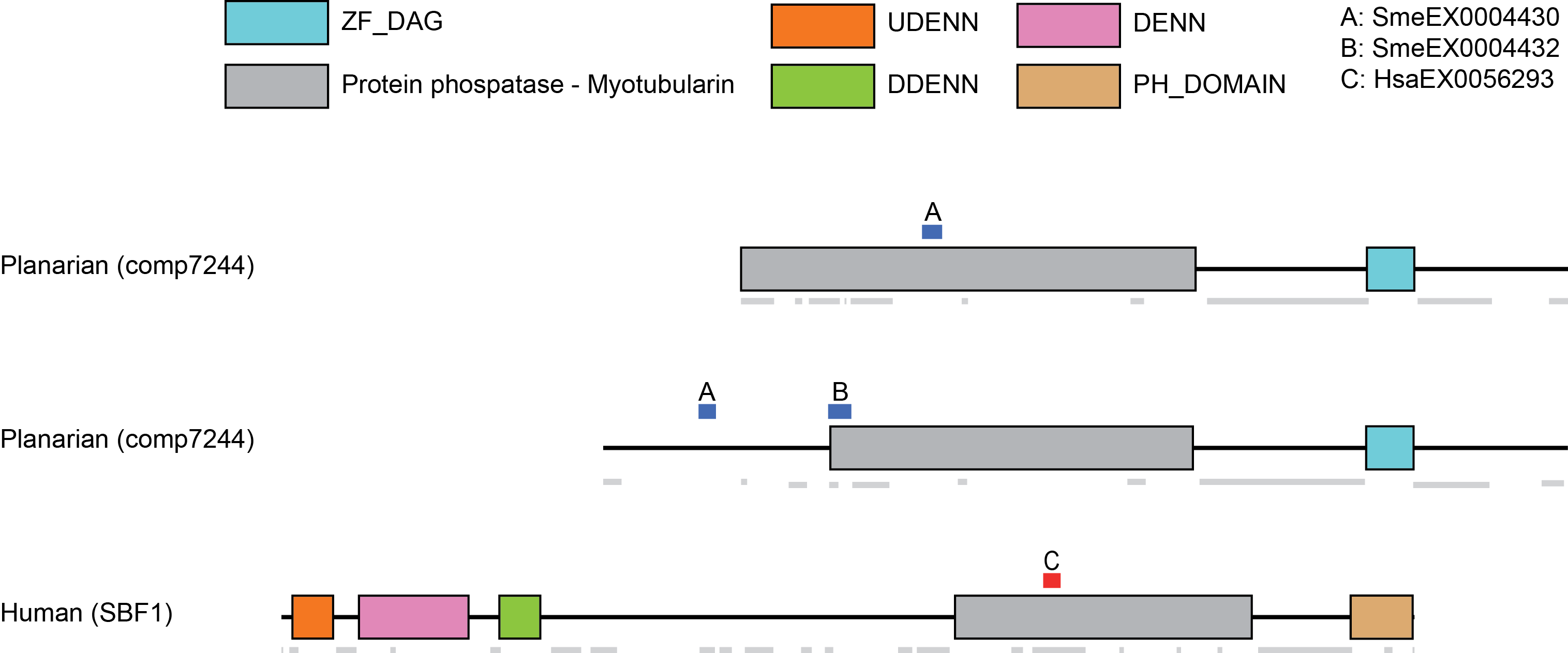


**- AS Exon key:**

SmeEX0004430

SmeEX0004432

HsaEX0056293

**- Protein Domain key:**

**Protein tyrosine phosphatases (PTP)**

**UDENN domain**

**DENN domain**

**DDENN domain**

**Zinc finger, DAG-binding domain**

**Pleckstrin-homology domain (PH)**

**- Planarian proteins:**

>transcript_comp7244_c0_seq1 (genome assembly is incorrect)

MIRLAEYFVVVGYDFEKSKHNESDGKIIQRFPSKDWPDYKYEVQVEMFCQPCGWILSDVKRPPTFFVAYLTNTVGEIYYLACLSFHEAISASLLDHGKPINASSAHSSYSRKIQHSNSSSHSRNLSTSSGIQTNKMNGESAGHETNGIIEHENDGFLEDPNLVKPDDKYAPKSLILLARHGHLDVLKNCLSIIYSIYMDPHREISLEYIVGNILSAVDAPSIGGSRISFTIGAGDKQVIQPSVCPTLPVTRSSVSMLFKHLGMQNVLAIFSSLINDQKILLYSQSLNRLTEACHALRSLLYPLQYGNVFIPIIPKTIIDVLSTPTPFLAGVHTSYRPMYDLIDVVMVDLDGGSAKYPDSIPLVSLCEPYNSITIDSLTKILNPDLLTADEVYPPFPSPPPDPVWQDKQLRAVFIRLFCRLFAGYRSCLTLTRIRVKPVIHFNESMFLLLRGLRNDSDEFIYRLLGCMSFGQFIQDRGTPYRVCDVFDEEYESMTELLRCKSDSFDDLQTIDKLAKKLYDNENRGSPVVHQLPQEAKLAHTRIHTQQFQLLDENLISDHIKTNTRNKSRREKQNQADLYDSTSLNKLETAEPRFVPHGLELDKQIFKTEALSDKNKVIREFVANIFNNHITEALKRRNTIRQDLKNRLIRRIFIDELYRQLYPDQSDTFGGQYDLNIAPYNSEISCERKALLGYQQFELVNDLMGEALRQEGQNEESGVVPIIMELSTKFCTQLNNIRYYSNMTHQIQEHPVWESQKFWITAFCDQVNNEIRQLYLHHSESQYRQRSERQNLAERHSANLHTSGGDRKRQLIDKSWSHNAGVSSQPSLAPVKSRLSVGNTSPTRIISESKRILYTNKMSALDMAAEEMKIIDIPDPEVQAQEEATVYAQILHFINLIVNFRIPLNIVDKNEQRSHSNHLQHQQIHNYPSDYGDGISMATSVSGGNCDSESGYEDAGYVNTSIINLETWLRKFVDKTASENNLTQANIENIKSKIEGIIEGHISNLEAIYPEVQNIPKVKKVEVTSPVLLPNEKVIIINNKESLRCHLLLDGRHEKLDMQRQREQDFEGLGGQTVTQGNPHKFVDEEDDFDALRPLLPAQGALFITNYRVVFHGAPKDPYQSNKLVCRSFPIAALKSIKKLGMKPFVFGVSNSQGVSFNLMSNRASRTRGGEGGCLDVIRIRSNTFQMLKIGLEQEDSSPEIKEELSIKLIALRYPPMLSFSFNTSNKIGTTHQKMNYCTGLPDETADTNKRCPSESISSLYSSSHNYSFSTTSPSIHSNLKQKNTFTRNTLRLAKEGINKISKIKGRTSPSSNSKSKGISESESERSSAIHDDENRDFVLDLNYTATDSKLLE**KLRSTLGYAEYSRLNIGSFPAMFNVNDRSGNRNELVRLIYNFNDRYQNVKSYQSMMVVPQVFNEASLKKFAKTHRKNRFPIITWRSMKTKAYLLRGAGFQAKGIMGKLLKQTSSQGNTTTASSVMFRNVTTTLQRGFQNMTLRPVYSERSLDEKQPDAEITAAKEQIRYFQAIVDISPSFDEIQQHLSKLDPLVHWNDNCRQNVMHHRKNPLEKAYKSVMAGGEKARKRIDNVFHIHNTEFDGGDKGLKFADEDDTLSQTSTSTIGFGADKKPIRLQNKEIKGYQRKGVRQIIQAKLQTTSLFRTSSPSNFKVIENSQSKSHGHNKNLNHPEDFARSTSFNLPTNRRSSKESLTSGPPIVDEIIDDEEGTMATTNVLNQSGDPGAHLGELRIVPLYILGEKSQMKTMSKSDSFHDIEFIAVENPNKQQIKEAYELLLKAALPDNVSSVPIISHLNPSGSSNRFQEVQPGSETSLAVAANKWILQIQCLLELSGTVVDLIDIQSASVAVCLEDGWDITSQIVSLAQVMLDPNYRTISGFWALIEKDWLMAGHKFYSRLYMSSNSKAGNFIPVFLQFLDAVHQLLNQFPMAFEFNDFYLKFFAYHHLSNRFHNFKMDCEGERIQWMRSLDEDCTPGSRLYEQNSIWNYIQHHHEEWPIFFNFLYNPRLSQEVLRPCTNLSMIDIWEYYL**NEDLSSGSVYDLDHFCPSYRKRNTQHYDPILNGAFNNSHVESVYAILGLREGCEVRNWQKIWNESCEESKEDLRRLESVFLNSDDEDYDADATLKSYSTPIKKSNKMANNCLISNAKYHKEEIQKIRTITYNDHKNTSLQRLEKRHLENPQENTIIHSTSDANYQILNSDHNLQKLPTHLEFQTICEICRNRTTLIGTVMLKCSRCNLACHDKCIRDFPNECKANVDHSLKESSPDFSESSELSPNNQRITPDTVTPTSILSNQTLQSAGNKLNDSPVDKILDFDRRKKSDDLIQASETTFSGDLYKLSQHTLFPSWQKKYFTLDIIRHELRYSDSKDVRSNSKHILLQDITGVRILTTLQNHQKRFPESAFLELQTDERTYKLGSDHPSVINEWMEKIQNSIQ

>comp7244_c0_seq1f631A 19.1 93.1 111.1 289.0 403.0 553.0 611.0 740.0 830.0 858.2

GFQNMTLRPVYSERSLDEKQPDAEITAAKEQIRYFQAIVDISPSFDEIQQHLSKLDPLVHWNDNCRQNVMHHRKNPLEKAYKSVMAGG**EKARTLLKNEVFKMSSSHDDLNTTNRRSSKESLTSGPPIVDEIIDDEEGTMATTNVLNQSGDPGAHLGELRIVPLYILGEKSQMKTMSKSDSFHDIEFIAVENPNKQQIKEAYELLLKAALPDNVSSVPIISHLNPSGSSNRFQEVQPGSETSLAVAANKWILQIQCLLELSGTVVDLIDIRSASVAVCLEDGWDITSQIVSLAQVMLDPNYRTISGFWALIEKDWLMAGHKFYSRLYMSSNSKAGNFIPVFLQFLDAVHQLLNQFPMAFEFNDFYLKFFAYHHLSNRFHNFKMDCEGERIQ**WMRSLDEDCTPGSRLYEQNSIWNYIQHHHEEWPIFFNFLYNPRLSQEVLRPCTNLSMIDIWEYYLNEDLSSGSVYDLDHFCPSYRKRNTQHYDPILNGAFNNSHVESVYAILGLREGCEVRNWQKIWNESCEESKEDLRRLESVFLNSDDEDYDADATLKSYSTPIKKSNKMANNCLISNAKYHKEEIQKIRTITYNDHKNTSLQRLEKRHLENPQENTIIHSISDANYQILNSDHNLQKLPTHLEFQT**ICEICRNRTTLIGTVMLKCSRCNLACHDKCIRDFPNECK**ANVDHSLKESSPDFSESSELSPNNQRITPDTVTPTSILSNQTLQSAGNKLNDSPVDKILDFDRRKKSDDLIQASETTFSGDLYKLSQHTLFPSWQKKYFTLDIIRHELRYSDSKDVRSNSKHILLQDITGVRILTTLQNHQKRFPESAFLE

>comp7244_c0_seq1f631A2

GFQNMTLRPVYSERSLDEKQPDAEITAAKEQIRYFQAIVDISPSFDEIQQHLSKLDPLVHWNDNCRQNVMHHRKNPLEKAYKSVMAGGEKAR**TLLKNEVFKMSSSHDDLNRIDNVFHIHNTEFDGGDKGLKFADEDDTLSQTSTSTIGFGADKKPIRLQNKEIKGYQRKGVRQIIQAKLQTTSLFRTSSPSNFKVIENSQSKSHGHNKNLNHPEDFARSTSFNLPSIGFISSFRGNAAAFTPLHNLSCTTNRRSSKESLTSGPPIVDEIIDDEEGTMATTNVLNQSGDPGAHLGELRIVPLYILGEKSQMKTMSKSDSFHDIEFIAVENPNKQQIKEAYELLLKAALPDNVSSVPIISHLNPSGSSNRFQEVQPGSETSLAVAANKWILQIQCLLELSGTVVDLIDIRSASVAVCLEDGWDITSQIVSLAQVMLDPNYRTISGFWALIEKDWLMAGHKFYSRLYMSSNSKAGNFIPVFLQFLDAVHQLLNQFPMAFEFNDFYLKFFAYHHLSNRFHNFKMDCEGERIQ**WMRSLDEDCTPGSRLYEQNSIWNYIQHHHEEWPIFFNFLYNPRLSQEVLRPCTNLSMIDIWEYYLNEDLSSGSVYDLDHFCPSYRKRNTQHYDPILNGAFNNSHVESVYAILGLREGCEVRNWQKIWNESCEESKEDLRRLESVFLNSDDEDYDADATLKSYSTPIKKSNKMANNCLISNAKYHKEEIQKIRTITYNDHKNTSLQRLEKRHLENPQENTIIHSISDANYQILNSDHNLQKLPTHLEFQT**ICEICRNRTTLIGTVMLKCSRCNLACHDKCIRDFPNECK**ANVDHSLKESSPDFSESSELSPNNQRITPDTVTPTSILSNQTLQSAGNKLNDSPVDKILDFDRRKKSDDLIQASETTFSGDLYKLSQHTLFPSWQKKYFTLDIIRHELRYSDSKDVRSNSKHILLQDITGVRILTTLQNHQKRFPESAFLE

**- Human proteins:**

>SBF1_ENSP00000370196 19.1 48.0 94.0 147.0 184.0 219.1 263.2 300.0 338.0 364.0 402.0 445.0 478.0 546.1 584.0 634.0 657.0 710.0 799.2 857.1 882.0 947.1 990.0 1049.2 1095.1 1164.2 1230.1 1276.1 1302.1 1363.0 1423.0 1457.0 1519.0 1561.1 1605.0 1682.0 1718.0 1788.2 1818.0 1862.0

**MARLADYFVLVAFGPHPRGSGEGQGQILQRFPEKDWEDNPFPQGIELFCQPSGWQLCPERNPPTFFVAVLTDINSERHYCACLTFW**EPAEPSQETTRVEDATEREEEGDEGGQTHLSPTAPAPSAQL**FAPKTLVLVSRLDHTEVFRNSLGLIYAIHVEGLNVCLENVIGNLLTCTVPLAGGSQRTISLGAGDRQVIQTPLADSLPVSRCSVALLFRQLGITNVLSLFCAALTEHKVLFLSRSYQRLADACRGLLALLFPLRYSFTYVPILPAQLLEVLSTPTPFIIGVNAAFQAETQELLDVIVADLDGG**TVTIPECVHIPPLPEPLQSQTHSVLSMVLDPELELADLAFPPPTTSTSSLKMQD**KELRAVFLRLFAQLLQGYRWCLHVVRIHPEPVIRFHKAAFLGQRGLVEDDFLMKVLEGMAFAGFVSER**GVPYRPTDLFDELVAHEVARMRADENHPQRVLRHVQELAEQLYKNENPYPAVAMHKVQRPGESSHLRRVPRPFPRLDEGTVQWIVDQAAAKMQGAPPAVKAERRTTVPSGPPMTAILERCSGLHVNSARRLEVVRNCISYVFEGKMLEAKKLLPAVLRALKGRAARRCLAQELHLHVQQNRAVLDHQQFDFVVRMMNCCLQDCTSLDEHGIAAALLPLVTAFCRKLSPGVTQFAYSCVQEHVVWSTPQFWEAMFYGDVQTHIRALYLEPTEDLAPAQEVGEAPSQEDERSALDVASEQRRLWPTLSREKQQELVQKEESTVFSQAIHYANRMSYLLLPLDSSKSRLLRERAGLGDLESASNSLVTNSMAGSVAESYDTESGFEDAETCDVAGAVVRFINRFVDKVCTESGVTSDHLKGLHVMVPDIVQMHIETLEAVQRESRRLPPIQKPKLLRPRLLPGEECVLDGLRVYLLPDGREEGAGGSAGGPALLPAEGAVFLTTYRVIFTGMPTDPLVGEQVVVRSFPVAALTKEKRISVQTPVDQLLQDGLQLRSCTFQLLKMAFDEEVGSDSAELFRKQLHKLRYPPDIRATFAFTLGSAHTPGRPPRVTKDKGPSLRTLSRNLVKNAKKTIGRQHVTRKKYNPPSWEHRGQPPPEDQEDEISVSEELEPSTLTPSSAL**KPSDRMTMSSLVERACCRDYQRLGLGTLSSSLSRAKSEPFRISPVNRMYAICRSYPGLLIVPQSVQDNALQRVSRCYRQNRFPVVCWRSGRSKAVLLRSGGLHGKGVVGLFKAQNAPSPGQSQADSSSLEQEKYLQAVVSSMPRYADASGRNTLSGFSSAHMGSHVPSPRARVTTLSNPMAASASRRTAPRGKWGSVRTSGRSSGLGTDVGSRLAGRDALAPPQANGGPPDPGFLRPQRAALYILGDKAQLKGVRSDPLQQWELVPIEVFEARQVKASFKKLLKACVPGCPAAEPSPASFLRSLEDSEWLIQIHKLLQVSVLVVELLDSGSSVLVGLEDGWDITTQVVSLVQLLSDPFYRTLEGFRLLVEKEWLSFGHRFSHRGAHTLAGQSSGFTPVFLQFLDCVHQVHLQFPMEFEFSQFYLKFLGYHHVSRRFRTFLLDSDYERIEL**GLLYEEKGERRGQVPCRSVWEYVDRLSKRTPVFHNYMYAPEDAEVLRPYSNVSNLKVWDFYTEETLAEGPPYDWELAQGPPEPPEEERSDGGAPQSRRRVVWPCYDSCPRAQPDAISRLLEELQRLETELGQPAERWKDTWDRVKAAQRLEGRPDGRGTPSSLLVSTAPHHRRSLGVYLQEGPVGSTLSLSLDSDQSSGSTTSGSRQAARRSTSTLYSQFQTAESE**NRSYEGTLYKKGAFMKPWKARWFVLDKTKHQLRYYDHRVDTECKGVIDLAEVEAVAPGTPTMGAPKTVDEKAFFDVKTTRRVYNFCAQDVPSAQQWVDRIQSCLS**DA

**Intron alignment inconclusive**

**ORTHOLOGOUS GROUP #14: *Erythrocyte membrane protein band 4.1-like (EPB41L)***


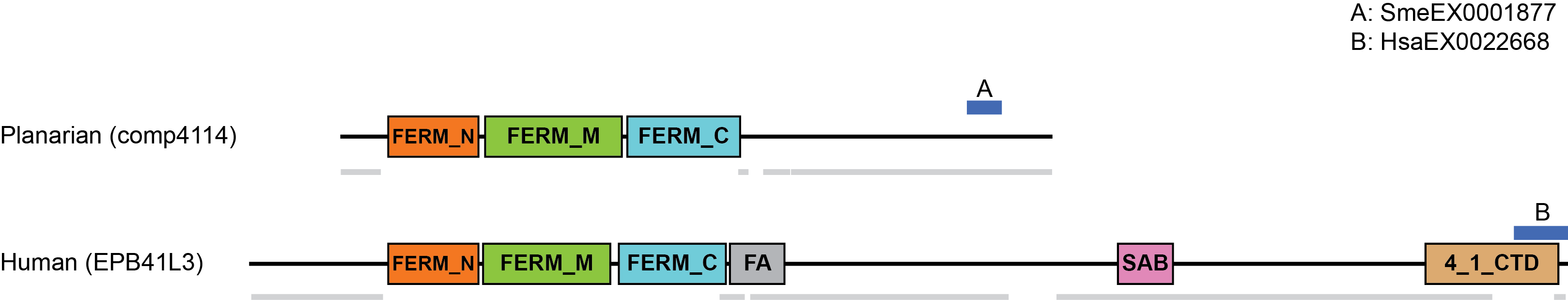


**- AS Exon key:**

SmeEX0001877

HsaEX0022668

**- Protein Domain key:**

**FERM_C_NBL4_NBL5**

**SAB domain**

**4_1_CTD**

**- Planarian proteins:**

>comp4114_c0_seq1 51.0 87.0 101.1 162.1 204.2 236.0 265.2 290.0 429.2 506.0 520.2 541.2 553.0 583.0

MATLLRLLTRRKKSNKRDYVDSEMQTRLKDTDLECKIVHLDGTDFTFFLEKRALGQKLEDYTFHQIEVNDDKNYFGLQYTDKKNINHWLDIGKKVRKQLEIGPPFTLRFRVKFFSSDPCSQLHDEYTRYLFVQQLKLLLRSGELRCDDKDMAAEIAALSLQGELGDYDEMEHSPAFISQFRFFPESQQTEELELLIISKFNLLKDRNILPSTADKQFLEKCRTLPDY**GVDQHNIKGKNDKDYILGLTPSGILIYEGQIKIGLFPWNNVTKLSFKKSRFKVVVQDVQPDDNGDVEHTFVFQSFDIVACKQMWKSCVEHHAFYR**LEGVRKPPTKFQQIFRLKSRFYASFHTEYQMAVKSTFGTSTRRSSTFTRAPSLRLAPRASFERNELERRKVQQRNRERVREENKNAAVQKMTSMGASGSGASTPSSAGVDTNYRPLSSPTKVNIAANMQQRNTPRSLGPSSIVVSHAIRKQVSEESTSSESSESIVAKPQVKPKPTNIPIVQKPNNARPNLNHGNQRNNISSQSDSSDSSDDDNDGDNSPPPPVAKRTVNVNHAPTLQQRSSRPVTSKQSFNPRNSNNEQIIQV*

**- Human proteins:**

>EPB41L3_ENSP00000343158 62.0 128.0 163.0 177.1 202.2 275.2 305.0 356.0 388.2 447.1 503.0 690.0 708.0 720.0 784.0 825.0 948.0 992.0 1025.0 1052.0

MTTESGSDSESKPDQEAEPQEAAGAQGRAGAPVPEPPKEEQQQALEQFAAAAAHSTPVRREVTDKEQEFAARAAKQLEYQQLEDDKLSQKSSSSKLSRSPLKIVKKPKSMQCKVILLDGSEYTCDVEKRSRGQVLFDKVCEHLNLLEKDYFGLTYRDAENQKNWLDPAKEIKKQVRSGAWHFSFNVKFYPPDPAQLSEDITRYYLCLQLRDDIVSGRLPCSFVTLALLGSYTVQSELGDYDPDECGSDYISEFRFAPNHTKELEDKVIELHKSHRGMTPAEAEMHFLENAKKLSM**YGVDLHHAKDSEGVEIMLGVCASGLLIYRDRLRINRFAWPKVLKISYKRNNFYIKIRPGEFEQFESTIGFKLPNHRAAKRLWKVCVEHHTFFRL**LLPEAPPKKFLTLGSKFRYSGRTQAQTRRASALIDRPAPYFERSSSKRYTMSRSLDGEVGTGQYATTKGISQTNLITTVTPEKKAEEERDEEEDKRRKGEEVTPISAIRHEGKSPGLGTDSCPLSPPSTHCAPTSPTELRRRCKENDCKLPGYEPSRAEHLPGEPALDSDGPGRPYLGDQDVAFSYRQQTGKGTTLFSFSLQLPESFPSLLDDDGYLSFPNLSETNLLPQSLQHYLPIRSPSLVPCFLFIFFFLLSASFSVPYALTLSFPLALCLCYLEPKAASLSASLDNDPSDSSEEETDSERTDTAADGETTATESDQEEDAEL**KAQELEKTQDDLMKHQTNISELKRTFLETSTDTAVTNEWEKRLSTSPVR**LAARQEDAPMIEPLVPEETKQSSGEKLMDGSEIFSLLESARKPTEFIGGVTSTSQSWVQKMETKTESSGIETEPTVHHLPLSTEKVVQETVLVEERRVVHASGDASYSAGDSGDAAAQPAFTGIKGKEGSALTEGAKEEGGEEVAKAVLEQEETAAASRERQEEQSAAIHISETLEQKPHFESSTVKTETISFGSVSPGGV**KLEISTKEVPVVHTETKTITYESSQVDPGTDLEPGVLMSAQTITSETTSTTTTTHITKTVKGGISETRIEKRIVITGDADIDHDQALAQAIKEAKEQHPDMSVTKVVVHKETEI**TPEDGED*

**Intron alignment not needed (different protein regions)**

**ORTHOLOGOUS GROUP #15: *Ankyrin (ANK)***


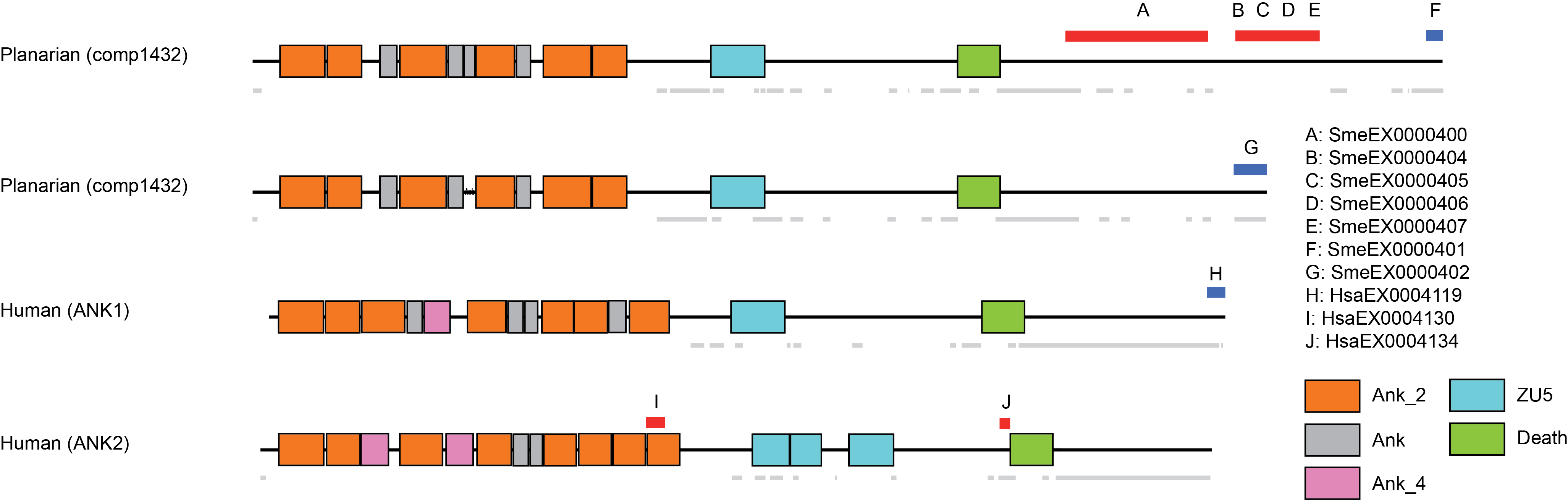


**- AS Exon key:**

SmeEX0000401 => Last exon

SmeEX0000402 => PTC (C1 in red font)

All after the death_Ank dom:

SmeEX0000400

SmeEX0000404

SmeEX0000405

SmeEX0000406

SmeEX0000407

...

HsaEX0004119 => PTC

HsaEX0004134 => between death and ZU5 domains

HsaEX0004130 => Exactly the last ANK domain

**- Protein Domain key:**

**Death_ank1**

**ZU5 domain**

**- Planarian proteins:**

>comp1432_c0_seq1 43.0 77.0 143.0 176.0 249.0 282.0 348.0 382.0 415.0 448.0 514.0 740.0 803.1 827.1 857.0 902.2 964.2 1029.1 1101.0 1196.2 1273.0 1380.1 1552.1 1604.1 1879.1 1939.1 1993.1 2017.1 2057.1 2099.1 2141.1 2183.1 2320.1

MFSCFGKNDHKLPPEDPGNISPISNENSLASPNLKKNSKASNSEKEHIFLRAAHNGYFSKVQEALNSNIDVNSCNSNGLNALHLASKGGHADIVQFLIDNGADIGAKTRKNNTAIHIASLAGSLEVVKILVKHGANINEQSQTGFTPLYMAAQENHVGVVKFLLDNGADINLSTTDNFTPLAVAIQQGHEETVSILLNKSNFLNSNDISNDIKLPPLHIATRKDDYHAVLLLLHHQEDVQAALTQASLNGFTALHVAAYYNSDKVGRLLLENGAPVDYTSKNNVTALHVAAKWGRNKMVKVLLEFNANVEMSTRDGIRPLHCAASQGHRDICALLIEHKANCNTRTKNGLVPLHMVAQGNHSELVEFFINQNSVNVNETSLDFLTPLHISSHCNNVEVAEALIKHGANVNDRALNGFVALHIAAKQNHVEFVEMLMSNLCMIDTQTESGFTALHIASIIGSESIAKKLVENNCEIDIPDQFDRTSLHMATIFNHFNIVVYLKEHGGSVDKQMKTGETCLMLAIKSEHKAIVEYLINQGASMSVSDSWGLTALHWATLVVRENILNSNCTDININEAIEIFKFVLDGCSRFDINRTTKNGISSLHLAADCGCLEIVHLLLDKGADINIISINGTTPLHIACKNNNSNVLLVLLESLSYDSLTMAYVSCPWSPMHFSSYFGSVDSTILLISHENDQTSVREKLRQRTPNGNTCLHLAVQQLQTDIVRVLLENDADFNLENEDGLSAFDMAKIVDDETIINLLGIDTTEMMTTDDKVYLFQREEFSDEEQLETLYMDSLNELEFFGIGKNSGEKCELQLSIMSLLDYATTIDSIRRADMSLKINNSLNRYRNITLSEIKQTRELLIAAQPASMISTISSTNTEMSPFDVEKWDFHRVTDLPV**NSGFLVSFLVDARGGYMQAQRMPQMKIFVPPDAAPAPTRIICHLLRPEGVANLPGMNDCDGFASRILQIHSSCSRFQKPILIEMPYIALLKSNDREIVILK**KNADSDTWKKHSNTFTEETLKAEFGSLYESITPVDQLESNRICRIITTDVPTYFSLISQFQRDVNMIGPDGGYIESKTDKRIKLTFPQGALMKKIQIGLEVQHVDPGIINHVVGDNHKISLSPIITVEPKRRKFRDLIEIQMGLPKGVTKNDIQDLKLLFSISEYITDWEDVTSSTELVLLENTNCMAHSTKISARVWLIYAPNVPNISELANKIYALHCPTPYVGKFLSYARRHDQEECQIRCLLITDDTPDKTLECQESFELIATGPEVEVINKLQYQIECAGNIVAVTKSDEAILFPVQSFVENRITLPIRVRNQDEPFQGKIAFIPHFQSLSKQIVKPITTLDIIIPEIIIDMVGSFRNPDRKHRVKNATLERIKGCQAIA**KTELDLKSVADAVGADWRALAEELELEADDINLVLNENCTDEEKAFMTLINWQTVLGFKATGNELNRALRQIGREDVVKSCI**GDISFVEDEAEVKSAIEQLNNDDKKTHDECWDDIIASKGKPSGYNESVDDSMIVETGDDWSQSHRESKLSDISDDKSLVGESTHDSESEIKPLVDPSKAVTLIDRTSSEKTAAFLLLVNQLDEVLPHPVIHESYNSADKIEACEESVVKSFKTDKQVANVDQTISLLNLINCQINDDFVHVDTVIDLDDFFPEPQEIDVDTLSQSDLNDYGENRDQNEFVLEVCIPEDKVEGLIKNYEGCEITNTLMNMSRDENPKVNDKILQIDLLKRRRYTLDKARLLQHNETFHFHAPVSSLRRCFSVDSRRGRGIFSLCSQVNSLHEASILERGDWPSNEDLKNIQTLQIGVEDSDSPLDTLDNKNDIEISEIIQTDIKTEEENSKIESKSYLKDILPDSPITDLDDLSLCDELEQSNEKKEVLEPRIVAGPLMSTMPSHLDIDLDTISPVTAGNFVYTQPVLFDDDKIVAENDYDTEKKEDINICYKNENPDNFKVDNSEVHSTSRYSVAGQTACNAPFTEDKTVVNKDFDLELLNQQNTNLKETEITYNEPVDELETKMNVNQNFECPIQMKADVEIDSEHSGQFHNTDTDVKGVLNQLATDVVDQELLKDDLQSLDKECVKPYDCNIDINACTNESLSYAEPYSTDYQFIEKNIVEKEESEKNVENERTPESYRDDENINESKVNNNPQFETEKEISIRSDNFVYDPISDEFVTLDNDEALNQPEKHSVLHEVIESDPQIQTEVEEEVEVLPDGRIVNRKITRKQISRIITEKVTRDIEPENEENIDSKVTFESPPEVIETEEELPDGTILKRTISTSMKTTSTTAKTVTEKEISKDENSADSAAMRQSEALTDNVSGKPTVKSRKNKNKKKSNKK*

>comp1432_c0_seq1f1063A 43.0 77.0 143.0 176.0 249.0 282.0 348.0 382.0 415.0 448.0 514.0 740.0 803.1 827.1 857.0 902.2 964.2 1029.1 1101.0 1196.2 1273.0 1380.1 1552.1 1604.1 1879.1 1939.1

MFSCFGKNDHKLPPEDPGNISPISNENSLASPNLKKNSKASNSEKEHIFLRAAHNGYFSKVQEALNSNIDVNSCNSNGLNALHLASKGGHADIVQFLIDNGADIGAKTRKNNTAIHIASLAGSLEVVKILVKHGANINEQSQTGFTPLYMAAQENHVGVVKFLLDNGADINLSTTDNFTPLAVAIQQGHEETVSILLNKSNFLNSNDISNDIKLPPLHIATRKDDYHAVLLLLHHQEDVQAALTQASLNGFTALHVAAYYNSDKVGRLLLENGAPVDYTSKNNVTALHVAAKWGRNKMVKVLLEFNANVEMSTRDGIRPLHCAASQGHRDICALLIEHKANCNTRTKNGLVPLHMVAQGNHSELVEFFINQNSVNVNETSLDFLTPLHISSHCNNVEVAEALIKHGANVNDRALNGFVALHIAAKQNHVEFVEMLMSNLCMIDTQTESGFTALHIASIIGSESIAKKLVENNCEIDIPDQFDRTSLHMATIFNHFNIVVYLKEHGGSVDKQMKTGETCLMLAIKSEHKAIVEYLINQGASMSVSDSWGLTALHWATLVVRENILNSNCTDININEAIEIFKFVLDGCSRFDINRTTKNGISSLHLAADCGCLEIVHLLLDKGADINIISINGTTPLHIACKNNNSNVLLVLLESLSYDSLTMAYVSCPWSPMHFSSYFGSVDSTILLISHENDQTSVREKLRQRTPNGNTCLHLAVQQLQTDIVRVLLENDADFNLENEDGLSAFDMAKIVDDETIINLLGIDTTEMMTTDDKVYLFQREEFSDEEQLETLYMDSLNELEFFGIGKNSGEKCELQLSIMSLLDYATTIDSIRRADMSLKINNSLNRYRNITLSEIKQTRELLIAAQPASMISTISSTNTEMSPFDVEKWDFHRVTDLPV**NSGFLVSFLVDARGGYMQAQRMPQMKIFVPPDAAPAPTRIICHLLRPEGVANLPGMNDCDGFASRILQIHSSCSRFQKPILIEMPYIALLKSNDREIVIL**KKNADSDTWKKHSNTFTEETLKAEFGSLYESITPVDQLESNRICRIITTDVPTYFSLISQFQRDVNMIGPDGGYIESKTDKRIKLTFPQGALMKKIQIGLEVQHVDPGIINHVVGDNHKISLSPIITVEPKRRKFRDLIEIQMGLPKGVTKNDIQDLKLLFSISEYITDWEDVTSSTELVLLENTNCMAHSTKISARVWLIYAPNVPNISELANKIYALHCPTPYVGKFLSYARRHDQEECQIRCLLITDDTPDKTLECQESFELIATGPEVEVINKLQYQIECAGNIVAVTKSDEAILFPVQSFVENRITLPIRVRNQDEPFQGKIAFIPHFQSLSKQIVKPITTLDIIIPEIIIDMVGSFRNPDRKHRVKNATLERIKGCQAIA**KTELDLKSVADAVGADWRALAEELELEADDINLVLNENCTDEEKAFMTLINWQTVLGFKATGNELNRALRQIGREDVVKSCI**GDISFVEDEAEVKSAIEQLNNDDKKTHDECWDDIIASKGKPSGYNESVDDSMIVETGDDWSQSHRESKLSDISDDKSLVGESTHDSESEIKPLVDPSKAVTLIDRTSSEKTAAFLLLVNQLDEVLPHPVIHESYNSADKIEACEESVVKSFKTDKQVANVDQTISLLNLINCQINDDFVHVDTVIDLDDFFPEPQEIDVDTLSQSDLNDYGENRDQNEFVLEVCIPEDKVEGLIKNYEGCEITNTLMNMSRDENPKVNDKILQIDLLKRRRYTLDKARLLQHNETFHFHAPVSSLRRCFSVDSRRGRGIFSLCSQVNSLHEASILERGDWPSNEDLKNIQTLQIGVEDSDSPLDTLDNKNDIEISEIIQTDIKTEEENSKIESKSYLKDILPDSPITDLDDLSLCDELEQSNEKKEVLEPRIVAGPLMSTMPSHLDIDLDTISPVTAGNFVYTQPVLFDDDKIVAENDYDTGMDSSNTVSSLISDSAMYGPEESESDSRSTMTIEENLSNLDLTFEEYETADESFESD*

>comp3277_c0_seq1 15.2 49.0 83.0 116.0 149.0 182.0 214.0 243.0 271.0 300.0 322.0 352.0 382.0 415.0 450.0 483.0 513.2

IFLPNYCNLILIRMSESEIFKDFVLQNNLKGIMDLSNPNLINKIIDITTNTRPIHIACNKGFFEMAEFMVNKKCNFEYSNKNGWRPLHHCCANGHLDIVQLLLSKDCEINCQTNDGMRPIHFACANGHFEIAKELIAKDCLLDIQTKQGWTSIHWAACNGHIEVVKLLIEKKCNTDIQDKDGCRPILIACAYGHIEIVKLLINNQNISFCPARDGVAPSVAGEIHLDKKCLEFLGGIQHSKQIFSCLKNIDCDKFSTFYRPITLMSIEEKTRISVLEFATENNLNNIIQIIMNKPVTDDEIQTVMEPLNNLTDRGRFLIEECFLYRCIKNRHMEGFKIFSSPQTINRPRKLVQKSFLEIAVEFSSRGFIKDILQKTIYDKEVWDRAKKDAEQNNQMEILSEISDREFVLSSRKQANLSILHTAVCDNNPDMVKYILDNPDLADKDMNNPRIGTALAFAHMLHHKLCIDILEQYNVDANTQNKLLETSLHEAIASGMHMKCQYLIDNSVEIDRVE*

**- Human proteins:**

>ANK1_ENSP00000289734 10.0 44.0 77.0 110.0 143.0 205.0 238.0 271.0 304.0 370.0 403.0 436.0 469.0 535.0 568.0 601.0 667.0 700.0 733.0 766.0 797.0 821.1 853.2 880.0 912.2 987.2 1039.1 1110.0 1178.1 1210.2 1287.0 1329.0 1369.0 1395.1 1420.1 1464.1 1513.1 1699.2 1799.0 1827.0 1849.0

MPYSVGFREADAATSFLRAARSGNLDKALDHLRNGVDINTCNQNGLNGLHLASKEGHVKMVVELLHKEIILETTTKKGNTALHIAALAGQDEVVRELVNYGANVNAQSQKGFTPLYMAAQENHLEVVKFLLENGANQNVATEDGFTPLAVALQQGHENVVAHLINYGTKGKVRLPALHIAARNDDTRTAAVLLQNDPNPDVLSKTGFTPLHIAAHYENLNVAQLLLNRGASVNFTPQNGITPLHIASRRGNVIMVRLLLDRGAQIETKTKDELTPLHCAARNGHVRISEILLDHGAPIQAKTKNGLSPIHMAAQGDHLDCVRLLLQYDAEIDDITLDHLTPLHVAAHCGHHRVAKVLLDKGAKPNSRALNGFTPLHIACKKNHVRVMELLLKTGASIDAVTESGLTPLHVASFMGHLPIVKNLLQRGASPNVSNVKVETPLHMAARAGHTEVAKYLLQNKAKVNAKAKDDQTPLHCAARIGHTNMVKLLLENNANPNLATTAGHTPLHIAAREGHVETVLALLEKEASQACMTKKGFTPLHVAAKYGKVRVAELLLERDAHPNAAGKNGLTPLHVAVHHNNLDIVKLLLPRGGSPHSPAWNGYTPLHIAAKQNQVEVARSLLQYGGSANAESVQGVTPLHLAAQEGHAEMVALLLSKQANGNLGNKSGLTPLHLVAQEGHVPVADVLIKHGVMVDATTRMGYTPLHVASHYGNIKLVKFLLQHQADVNAKTKLGYSPLHQAAQQGHTDIVTLLLKNGASPNEVSSDGTTPLAIAKRLGYISVTDVLKVVTDETSFVLVSDKHRMSFPETVDEILDVSEDEGEELISFKAERRDSRDVDEEKELLDFVPKLDQVVESPAIPRIPCAMPETVVIRSEEQEQASKEYDEDSLIPSSPATETSDNISPVASPVH**TGFLVSFMVDARGGSMRGSRHNGLRVVIPPRTCAAPTRITCRLVKPQKLSTPPPLAEEEGLASRIIALGPTGAQFLSPVIVEIPHFASHGRGDRELVVLRSENGS**VWKEHRSRYGESYLDQILNGMDEELGSLEELEKKRVCRIITTDFPLYFVIMSRLCQDYDTIGPEGGSLKSKLVPLVQATFPENAVTKRVKLALQAQPVPDELVTKLLGNQATFSPIVTVEPRRRKFHRPIGLRIPLPPSWTDNPRDSGEGDTTSLRLLCSVIGGTDQAQWEDITGTTKLVYANECANFTTNVSARFWLSDCPRTAEAVNFATLLYKELTAVPYMAKFVIFAKMNDPREGRLRCYCMTDDKVDKTLEQHENFVEVARSRDIEVLEGMSLFAELSGNLVPVKKAAQQRSFHFQSFRENRLAMPVKVRDSSREPGGSLSFLRKAMKYEDTQHILCHLNITMPPCAKGSGAEDRRRTPTPLALRYSILSESTPGSLSGT**EQAEMKMAVISEHLGLSWAELARELQFSVEDINRIRVENPNSLLEQSVALLNLWVIREGQNANMENLYTALQSIDRGEIVNMLE**GSGRQSRNLKPDRRHTDRDYSLSPSQMNGYSSLQDELLSPASLGCALSSPLRADQYWNEVAVLDAIPLAATEHDTMLEMSDMQVWSAGLTPSLVTAEDSSLECSKAEDSDATGHEWKLEGALSEEPRGPELGSLELVEDDTVDSDATNGLIDLLEQEEGQRSEEKLPGSKRQDDATGAGQDSENEVSLVSGHQRGQARITHSPTVSQVTERSQDRLQDWDADGSIVSYLQDAAQGSWQEEVTQGPHSFQGTSTMTEGLEPGGSQEYEKVLVSVSEHTWTEQPEAESSQADRDRRQQGQEEQVQEAKNTFTQVVQGNEFQNIPGEQVTEEQFTDEQGNIVTKKIIRKVVRQIDLSSADAAQEHEEVELRGSGLQPDLIEGRKGAQIVKRASLKRGKQ*

>ANK2_ENSP00000378044 8.0 42.0 75.0 108.0 141.0 203.0 211.0 244.0 277.0 310.0 376.0 409.0 442.0 475.0 541.0 574.0 607.0 673.0 706.0 739.0 772.0 805.0 829.1 877.2 912.0 946.2 958.2 1033.2 1066.2 1118.1 1189.0 1257.1 1289.2 1366.0 1408.0 1449.0 1460.0 1467.1 1492.1 1536.1 1584.1 1679.2 1777.0 1805.0 1860.0

MTTMLQKSDSNASFLRAARAGNLDKVVEYLKGGIDINTCNQNGLNALHLAAKEGHVGLVQELLGRGSSVDSATKKGNTALHIASLAGQAEVVKVLVKEGANINAQSQNGFTPLYMAAQENHIDVVKYLLENGANQSTATEDGFTPLAVALQQGHNQAVAILLENDTKGKVRLPALHIAARKDDTKSAALLLQNDHNADVQSKMMVNRTTESGFTPLHIAAHYGNVNVATLLLNRGAAVDFTARNGITPLHVASKRGNTNMVKLLLDRGGQIDAKTRDGLTPLHCAARSGHDQVVELLLERGAPLLARTKNGLSPLHMAAQGDHVECVKHLLQHKAPVDDVTLDYLTALHVAAHCGHYRVTKLLLDKRANPNARALNGFTPLHIACKKNRIKVMELLVKYGASIQAITESGLTPIHVAAFMGHLNIVLLLLQNGASPDVTNIRGETALHMAARAGQVEVVRCLLRNGALVDARAREEQTPLHIASRLGKTEIVQLLLQHMAHPDAATTNGYTPLHISAREGQVDVASVLLEAGAAHSLATKKGFTPLHVAAKYGSLDVAKLLLQRRAAADSAGKNGLTPLHVAAHYDNQKVALLLLEKGASPHATAKNGYTPLHIAAKKNQMQIASTLLNYGAETNIVTKQGVTPLHLASQEGHTDMVTLLLDKGANIHMSTKSGLTSLHLAAQEDKVNVADILTKHGADQDAHTKLGYTPLIVACHYGNVKMVNFLLKQGANVNAKTK**NGYTPLHQAAQQGHTHIINVLLQHGAKPNATTA**NGNTALAIAKRLGYISVVDTLKVVTEEVTTTTTTITEKHKLNVPETMTEVLDVSDEEGDDTMTGDGGEYLRPEDLKELGDDSLPSSQFLDGMNYLRYSLEGGRSDSLRSFSSDRSHTLSHASYLRDSAVMDDSVVIPSHQVSTLAKEAERNSYRLSWGTENLDNVALSSSPIHSGRASPCLERDN**SSFLVSFMVDARGGAMRGCRHNGLRIIIPPRKCTAPTRVTCRLVKRHRLATMPPMVEGEGLASRLIEVGPSGAQFLGKLHLPTAPPPLNEGESLVSRILQLGPPGTKFLGPVIVEIPHFAALRGKERELVVLRSENGDSWK**EHFCDYTEDELNEILNGMDEVLDSPEDLEKKRICRIITRDFPQYFAVVSRIKQDSNLIGPEGGVLSSTVVPQVQAVFPEGALTKRIRVGLQAQPMHSELVKKILGNKATFSPIVTLEPRRRKFHKPITMTIPVPKASSDVMLNGFGGDAPTLRLLCSITGGTTPAQWEDITGTTPLTFVNECVSFTTNVSARFWLIDCRQIQESVTFASQVYREIICVPYMAKFVVFAKSHDPIEARLRCFCMTDDKVDKTLEQQENFAEVARSRDVEVLEGKPIYVDCFGNLVPLTKSGQHHIFSFFAFKENRLPLFVKVRDTTQEPCGRLSFMKEPKSTRGLVHQAICNLNITLPIYTKESESDQEQEEEIDMTSEKNPQDEQER**IEERLAYIADHLGFSWTELARELDFTEEQIHQIRIENPNSLQDQSHALLKYWLERDGKHATDTNLVECLTKINRMDIVHLMETNT**EPLQERISHSYAEIEQTITLDHSEGFSVLQEELCTAQHKQKEEQAVSKESETCDHPPIVSEEDISVGYSTFQDGVPKTEGDSSATALFPQTHKEQVQQDFSGKMQDLPEESSLEYQQEYFVTTPGTETSETQKAMIVPSSPSKTPEEVSTPAEEEKLYLQTPTSSERGGSPIIQEPEEPSEHREESSPRKTSLVIVESADNQPETCERLDEDAAFEKGDDMPEIPPETVTEEEYIDEHGHTVVKKVTRKIIRRYVSSEGTEKEEIMVQGMPQEPVNIEEGDGYSKVIKRVVLKSDTEQSEDNNE*

Sme1: comp1432_c0_seq1f1063A

Hsa1: ANK1_ENSP00000289734

Sme1 MFSCFGKNDHKLPPEDPGNISPISNENSLASPNLKKNSKASNS 0 EKEHIFLRAAHNGYFSKVQEALNSNIDVNSCNSN 0 GLNALHLASKGGHADIVQFLIDNGADIGAKTRK * NNTAIHIASLAGSLEVVKILV Sme1

Hsa1 ---------------------------------MPYSVGFREA 0 DAATSFLRAARSGNLDKALDHLRNGVDINTCNQN 0 GLNGLHLASKEGHVKMVVELLHKEIILETTTKK 0 GNTALHIAALAGQDEVVRELV Hsa1

Sme1 KHGANINEQSQT 0 GFTPLYMAAQENHVGVVKFLLDNGADINLSTTD 0 NFTPLAVAIQQGHEETVSILLNKSNFLNSNDISNDIKLPPLHIATRKDDYHAVLLLLHHQEDVQAALTQASLN 0 GFTALHVAAYYNS Sme1

Hsa1 NYGANVNAQSQK 0 GFTPLYMAAQENHLEVVKFLLENGANQNVATED 0 GFTPLAVALQQGHENVVAHLIN-------YGTKGKVRLPALHIAARNDDTRTAAVLLQNDPNP----DVLSKT 0 GFTPLHIAAHYEN Hsa1

Sme1 DKVGRLLLENGAPVDYTSKN 0 NVTALHVAAKWGRNKMVKVLLEFNANVEMSTRD * GIRPLHCAASQGHRDICALLIEHKANCNTRTKN 0 GLVPLHMVAQGNHSELVEFFINQNSVNVNETSLD 0 FLTPLHIS Sme1

Hsa1 LNVAQLLLNRGASVNFTPQN 0 GITPLHIASRRGNVIMVRLLLDRGAQIETKTKD 0 ELTPLHCAARNGHVRISEILLDHGAPIQAKTKN 0 GLSPIHMAAQGDHLDCVRLLLQYD-AEIDDITLD * HLTPLHVA Hsa1

Sme1 SHCNNVEVAEALIKHGANVNDRALN 0 GFVALHIAAKQNHVEFVEMLMSNLCMIDTQTES 0 GFTALHIASIIGSESIAKKLVENNCEIDIPDQF * DRTSLHMATIFNHFNIVVYLKEHGGSVDKQMKT 0 GETC Sme1

Hsa1 AHCGHHRVAKVLLDKGAKPNSRALN 0 GFTPLHIACKKNHVRVMELLLKTGASIDAVTES 0 GLTPLHVASFMGHLPIVKNLLQRGASPNVSNVK 0 VETPLHMAARAGHTEVAKYLLQNKAKVNAKAKD 0 DQTP Hsa1

Sme1 LMLAIKSEHKAIVEYLINQGASMSVSDSWGLTALHWAT--------LVVRENILNSNCTDIN * -------INEAIEIFKFVLDGCSRFDINRTTKN * GISSLHLAADCGCLEIVHLLLDKGADINIISIN * GTT Sme1

Hsa1 LHCAARIGHTNMVKLLLENNANPNLATTAGHTPLHIAAREGHVETVLALLEKEASQACMTKK 0 GFTPLHVAAKYGKVRVAELLLERDAHPNAAGKN 0 GLTPLHVAVHHNNLDIVKLLLPRGGSPHSPAWN 0 GYT Hsa1

Sme1 PLHIACKNNNSNVLLVLLESLSYDSLTMAYVSCPWSPMHFSSYFGSVDSTILLISHEND------- * ------------QTSVREKLRQR-------TPN * GNTCLHLAVQQLQTDIVRVLLENDADFNLENED 0 Sme1

Hsa1 PLHIAAKQNQVEVARSLLQ---YGGSANAESVQGVTPLHLAAQEGHAEMVALLLSKQANGNLGNKS 0 GLTPLHLVAQEGHVPVADVLIKHGVMVDATTRM 0 GYTPLHVASHYGNIKLVKFLLQHQADVNAKTKL 0 Hsa1

Sme1 GLSAFDMAKIVDDETIINLLGID--TTEMMTTD * DKVYLFQREEFSDEEQLETLYMDSLNELEFF * G 1 IGKN--------------SGEKC * ELQLSIMSLLDYATT 1 IDSIR-RADMSLKINNS * L Sme1

Hsa1 GYSPLHQAAQQGHTDIVTLLLKNGASPNEVSSD 0 GTTPLAIAKRLGYISVTDVLKVVTDETSFVL 0 V * SDKHRMSFPETVDEILDVSEDEG 1 EELISFKAERRDSRD * VDEEKELLDFVPKLDQV 2 V Hsa1

Sme1 NRYRNITLSEIKQ 0 TRELLIAAQPASM * ISTISSTNTEMSPFDVEKWDFHRVTDLPVNSG 2 FLVSFLVDARGGYMQAQRMPQMKIFVPPDAAPAPTRIICHLLRPEGVANLPGMNDCDGFASR 2 ILQIHSSC Sme1

Hsa1 ESPAIPRIPCAMP * ETVVIRSEEQEQA 0 SKEYDEDSLIPSSPATETSDNISPVASPVHTG 2 FLVSFMVDARGGSMRGSRHNGLRVVIPPRTCAAPTRITCRLVKPQKLSTPPPLAEEEGLASR * IIALGPTG Hsa1

Sme1 SRFQK * PILIEMPYIALLKSNDREIVILKKNADSDTWKKHSNTFTEETLKAEFGSLYE 1 S * ITPVDQLESNRICRIITTDVPTYFSLISQFQRDVNMIGPDGGYIESKTDKRIKLTFPQGALMKKIQIGLEV 0 Sme1

Hsa1 AQFLS 2 PVIVEIPHFASHGRGDRELVVLRS-ENGSVWKEHRSRYGESYLDQILNGMDE * E 1 LGSLEELEKKRVCRIITTDFPLYFVIMSRLCQDYDTIGPEGGSLKSKLVPLVQATFPENAVTKRVKLALQA 0 Hsa1

Sme1 QHVDPGIINHVVGDNHKISLSPIITVEPKRRKFRDLIEIQMGLPKGVTKN-------DIQDLKLLFSIS- * -EYITDWEDVTSSTELVLLENTNCMAHSTKISAR 2 VWLIYAPNVPNISELANKIYALHCPTPYV Sme1

Hsa1 QPVPDELVTKLLGN--QATFSPIVTVEPRRRKFHRPIGLRIPLPPSWTDNPRDSGEGDTTSLRLLCSVIG 1 GTDQAQWEDITGTTKLVYAN--ECANFTTNVSAR 2 FWLSDCPRTAEAVNFATLLYKELTAVPYM Hsa1

Sme1 GKFLSYARRHDQEECQIRCLLITDDTPDKTLECQESFELIATGPEVEV 0 INKLQYQIECAGNIVAVTKSDEAILFPVQSFVENRITLPIRV * RNQDEPFQGKIAFIPHFQSLSKQIVKPITTLDIIIPEIII * D Sme1

Hsa1 AKFVIFAKMNDPREGRLRCYCMTDDKVDKTLEQHENFVEVARSRDIEV 0 LEGMSLFAELSGNLVPVKKAAQQRSFHFQSFRENRLAMPVKV 0 RDSSREPGGSLSFLRKAMKYEDTQHILCHLNITMPPCAKG 0 S Hsa1

Sme1 MVGSFRNPDRKHRVKNATLERIKG 1 - * -CQAIAKTELDLKSVADAVGADWRA * LAEELELEADDINLVLNEN-CTDEEKAFMTLINWQTVLGFKATG * NELNRALRQIGREDVVKSCIGDISFVEDEAEVKS Sme1

Hsa1 GAEDRRRTPTPLALRYSILSESTP * G 1 SLSGTEQAEMKMAVISEHLGLSWAE 1 LARELQFSVEDINRIRVENPNSLLEQSVALLNLWVIREGQNANM 1 ENLYTALQSIDRGEIVN----------------- Hsa1

Sme1 AIEQLNNDDKKTHDECWDDIIASKGKPSGYNESVDDSMIVETGDDWSQSHRESKLSDISDDKSLVGESTHD 1 SESEIKPLVDPSKAVTLIDRTSSEKTAAFLLLVNQLDEVLPHPVIHESYNSA 1 DKIEACEESVV Sme1

Hsa1 ---------------------------------------MLEGSGRQSRNLKPDRRHTDRDYSLSPSQMNG 1 YSSLQDELLSPASLGCALS--SPLRADQYWNEVAVLDAIPLAATEHDTMLEM * SDMQVWSAGLT Hsa1

Sme1 KSFKTDKQVANVDQTISLLNLINCQINDDFVHVDTVIDLDDFFPEPQEIDVDTLSQSDLNDYGENRDQNEFVLEVCIPEDKVEGLIKNYEGCEITNTLMNMSRDENPKVNDKILQIDLLKRRRYTLDKARLLQHNETFHF Sme1

Hsa1 PSLVTAEDSS-----------LECSKAEDSDATGHEWKLEGALSE--------------EPRGPELGSLELVEDDTVDSDATNGLIDLLE--------QEEGQRSEEKLPGSKRQDDATGAGQDSENEVSLVSG------ Hsa1

Sme1 HAPVSSLRRCFSVDSRRGRGIFSLCSQVNSLHEASILE * RGDWPSN-EDLKNIQTLQIGVEDSDSPLDTLDNKNDIEISEIIQTDIKTEEENSKIESKSYLKDILPDSPITDLDDLSLCDELEQSN 1 EKKEVLEPR Sme1

Hsa1 --------------HQRGQARITHSPTVSQVTERSQDR 2 LQDWDADGSIVSYLQDAAQGSWQEEVTQGPHSFQGTSTMTEGLEPGGSQEYEKVLVSVSEHTWTEQPEAESSQADRDRRQQGQEEQV * QEAKNTFTQ Hsa1

Sme1 IVAG * PLMSTMPSHLDIDLDTISPVTAGNFVYTQPVLFDDDKIVAENDYDTG 1 MDS * SNTVSSLISDSAMYGPEESESD * SRSTMTIEENLSNLDLTFEEYETADESFESD_ Sme1

Hsa1 VVQG 0 NEFQNIPGEQ----------------------VTEEQFTDEQGNIVT * KKI 0 IRKVVRQIDLSSADAAQEHEEV 0 ELRGSGLQPDLIEGRKGAQIVKRASLKRGKQ_ Hsa1

**Intron alignment not needed for other exons (different protein regions)**
